# Brain-Wide Synaptic Inputs to Aromatase-Expressing Neurons in the Medial Amygdala Suggest Complex Circuitry for Modulating Social Behavior

**DOI:** 10.1523/ENEURO.0329-21.2021

**Published:** 2022-03-11

**Authors:** Joseph Dwyer, Diane A. Kelly, Joseph Bergan

**Affiliations:** 1 Neuroscience and Behavior Program, University of Massachusetts, Amherst, Amherst, Massachusetts 01003; 2 Department of Psychological and Brain Sciences, University of Massachusetts, Amherst, Amherst, Massachusetts 01003

**Keywords:** aromatase, circuits, medial amygdala, rabies, social behavior, synapse

## Abstract

Here, we reveal an unbiased view of the brain regions that provide specific inputs to aromatase-expressing cells in the medial amygdala, neurons that play an outsized role in the production of sex-specific social behaviors, using rabies tracing and light sheet microscopy. While the downstream projections from these cells are known, the specific inputs to the aromatase-expressing cells in the medial amygdala remained unknown. We observed established connections to the medial amygdala (e.g., bed nucleus of the stria terminalis and accessory olfactory bulb) indicating that aromatase neurons are a major target cell type for efferent input including from regions associated with parenting and aggression. We also identified novel and unexpected inputs from areas involved in metabolism, fear and anxiety, and memory and cognition. These results confirm the central role of the medial amygdala in sex-specific social recognition and social behavior, and point to an expanded role for its aromatase-expressing neurons in the integration of multiple sensory and homeostatic factors, which are likely used to modulate many other social behaviors.

## Significance Statement

Aromatase-expressing neurons in the medial amygdala play a significant role in producing critically important social behaviors, including parenting, aggression, and reproduction. We used rabies tracing and light sheet microscopy to reveal an unbiased view of many brain regions that provide direct synaptic input to aromatase neurons, and to observe both well established and previously unknown inputs from areas involved in parenting and aggression, metabolism, fear and anxiety, and memory and cognition. These results confirm the central role of the medial amygdala in social recognition behavior and point to an expanded role for its aromatase-expressing neurons in the integration of multiple sensory and homeostatic factors, which can be used to modulate many other social behaviors.

## Introduction

Social behaviors are necessary for communication, survival, and reproduction in species throughout the animal kingdom (Darwin et al., 1871; Green and Marler, 1979; Ueda et al., 2020), and depend on the integration of external and internal sensory cues. Nearly all sensory systems have been linked to social behavior (Pomerantz et al., 1983; Strasser and Dixon, 1986; Contreras and Agmo, 1993; Keller et al., 2006a, b). For example, the diverse, and often beautiful, vocalizations of songbirds signal their species, individual identity, territorial ownership, and readiness to mate (Marler, 1961). In humans, visual cues produced in the face and hands provide information about another’s identity and emotional state (Haxby et al., 2002). For many species, chemosensory cues are particularly important to detect, identify, and respond to social partners (Schwanzel-Fukuda and Pfaff, 1991; Dulac and Axel, 1995; Bradley et al., 2002; Keller et al., 2006b; Gonzalez et al., 2017). In mice, chemosensory information, transduced by the main olfactory epithelium and the vomeronasal organ (VNO), provides the primary sensory input to a conserved network of brain regions dedicated to executing social behaviors (Newman, 1999).

The core brain regions that make up the social behavior network (SBN) are well established and conserved for all vertebrate species examined thus far (Goodson, 2005). However, identifying the specific neural circuits that generate social behaviors has been challenging because of both the heterogeneity of cell populations in these regions and the interwoven and reciprocal nature of both the behaviors themselves and the underlying circuits. Thus, a current goal in neuroscience is to define specific contributions of well defined populations of neurons to circuit function and, ultimately, to behaviors. Genetic and viral tools allow precise characterization of the connections made by individual populations of neurons and have been instrumental in defining cell type-specific wiring diagrams that mediate specific behaviors (Wickersham et al., 2007a; Watabe-Uchida et al., 2012; Menegas et al., 2015; Kohl et al., 2018).

The medial amygdala (MeA), a central node in the SBN, receives sensory input directly from the accessory olfactory bulb (AOB) and the main olfactory bulb (MOB). The MeA integrates chemosensory signals with modulatory input from other brain regions (Ferguson et al., 2001; Yao et al., 2017) and circulating hormones (Cooke et al., 1999; Cooke and Woolley, 2005) to guide behavioral responses through its projections to efferent behavioral centers (Wu et al., 2009). MeA neurons in mice display sex differences in sensory responses to social stimuli (Samuelsen and Meredith, 2009; Bergan et al., 2014; Kim et al., 2017) and robust anatomic sex differences (Cooke et al., 1999; Wu et al., 2009; Billing et al., 2020). Social behaviors that are tightly associated with the MeA, including parenting, mate choice, and aggression, also display clear sex differences (Vochteloo and Koolhaas, 1987; Yao et al., 2017; Chen and Hong, 2018). Each of these behaviors are critical for survival and reproduction. Their dependence on the sex, age, and neuroendocrine status of an animal offers an opportunity to understand how the function of a common neural circuit can be modified to meet the specific behavioral requirements of an individual animal.

A large population of MeA neurons express the enzyme aromatase, which converts testosterone to estradiol, is critical for aggression in both sexes, and is known to shape the pattern of sensory responses in the MeA (Bergan et al., 2014; Unger et al., 2015). To fully understand the role of aromatase neurons in social behavior, however, we must first identify the inputs they receive and the circuits they form. Past experiments have addressed the electrophysiology of MeA neurons (Meredith and Westberry, 2004; Binns and Brennan, 2005; Bergan et al., 2014; Yao et al., 2017; Li and Dulac, 2018) as well as the efferent targets of the MeA aromatase population (Wu et al., 2009). Recently, a sex-specific connection from AOB mitral and tufted neurons to aromatase neurons of the MeA was identified using rabies-based viral tracing from aromatase neurons (Billing et al., 2020). However, the MeA integrates information from a large network of brain regions. Here we use rabies-based circuit mapping in conjunction with whole-brain cleared tissue imaging to exhaustively characterize the sources of synaptic input to aromatase neurons in the MeA, and to identify the information channels that shape aromatase-dependent social behaviors.

## Materials and Methods

### Animal use

All experiments were performed in strict compliance with the National Institutes of Health. All animals were handled according to a protocol approved by the University of Massachusetts, Amherst, Institutional Animal Care and Use Committee (protocol #2018–0014 and #2017–0060).

Fifteen adult mice (male, *n* = 9; female, *n* = 6; age, 2–8 months) from an existing transgenic mouse line (Cyp19a1-Cre; The Jackson Laboratory) were housed in a temperature-controlled (22°C) and light-controlled (12 h light/dark cycle) facility, with *ad libitum* access to food and water. The Cyp19a1-Cre transgenic line was generated by BAC (bacterial artificial chromosome) recombination (Yao et al., 2017); its expression pattern faithfully recapitulates endogenous aromatase expression (Yao et al., 2017) and displays no known behavioral deficits in either heterozygous or homozygous animals.

### Viral injections

We used a retrograde tracing system based on the modified rabies virus (EnvA-SADΔG-EGFP; Wickersham et al., 2007b) that uses two consecutive stereotaxic injections to visualize →MeA^arom+^. In the first injection, 500 nl of AAV8-FLEX-TVA and AAV8-FLEX-RG-mCherry (mixed 1:1; Watabe-Uchida et al., 2012) were injected into either the left or right MeA (distance from bregma, −1.9 mm; lateral, 1.9 mm; depth, 4.5–5.5 mm). After 14 d, 500 nl of SADΔG-EGFP(EnvA) virus (from Viral Vector Core, Salk Institute, La Jolla, CA) was injected into the MeA at the same stereotaxic coordinates (see Fig. 2*B*; Wickersham et al., 2007a, b). All stereotaxic injections used a cold capillary (20-μm-diameter tip) coupled to a hydraulic manipulator (Narishige; Wu et al., 2014). All adeno-associated viruses (AAVs) were produced by the UNC Vector Core Facility (University of North Carolina, Chapel Hill, NC).

The rabies virus requires the cell surface expression of TVA to enter a cell and is therefore limited to infecting Cre-expressing neurons (see Fig. 2*A*). Once inside a neuron, the modified rabies virus requires coexpression of the envelope rabies glycoprotein (RG) to be packaged for retrograde trans-synaptic spread. The efficiency of viral infection was determined by injecting the same viruses into a double-transgenic line that labels all Cre-expressing neurons with the R26-lsl-tdTomato reporter (see Fig. 2*C*; Madisen et al., 2010). This control experiment allowed us to estimate the infection rate (percentage of the target population labeled by rabies; see Fig. 2*D*).

### Tissue processing

Ten days after the final injection, animals were deeply anesthetized with isoflurane and perfused with 50 ml of cold PBS followed by 25 ml of cold PFA (4% in PBS). The brain was extracted and postfixed in 25 ml hydrogel (4% acrylamide, 4% PFA, 0.05% bis-acrylamide, and 0.25% VA-044 initiator suspended in 0.01 m PBS; Isogai et al., 2017) at 4°C overnight.

After 12–24 h of incubation in hydrogel, oxygen was flushed from the hydrogel by bubbling the liquid hydrogel solution with nitrogen. The tissue container was resealed and transferred to a 37°C water bath until polymerization was complete (at least 2 h). Excess hydrogel was removed from the brain manually, and the tissue sample was incubated to SDS-clearing solution (10 mm SDS in 0.1 m borate buffer, pH 8.5) for 2 d at 37°C before magnetohydrodynamic (MHD)-accelerated clearing (Dwyer et al., 2021).

Active clearing was performed in a 5 gallon container containing SDS-clearing solution using a custom MHD clearing device (Dwyer et al., 2021) that rapidly removed unbound lipids from the tissue sample at 37°C. Brains were cleared until bright white and translucent, which typically took 24–48 h. Brains were then transferred to 0.01 m PBS for 24 h. Brains were transferred from PBS to an OptiView imaging solution (Isogai et al., 2017) with a refractive index 1.45 and incubated at 37°C for 2 d before imaging.

### Image acquisition and processing

Images were acquired with a Z.1 Lightsheet microscope (Carl Zeiss). Rabies-labeled EGFP-expressing neurons were excited with a 488 nm laser. A 561 nm and/or 647 nm laser was used to produce an autofluorescence image for subsequent background subtraction and isolation of the GFP signal. Images were collected with a 5× magnification objective lens with pco.edge scientific CMOS cameras (PCO). The entire brain was imaged in the horizontal orientation from both the dorsal and ventral surfaces. This produced a series of slightly overlapping 3D image stacks for each brain. 3D image stacks were saved at 1–5 μm resolution and reconstructed to form a 3D image of the entire brain using custom MATLAB scripts (MathWorks).

### Cell counting

Rabies-labeled cells were identified using a human-trained computer vision algorithm (Ilastik, Heidelberg University, Heidelberg, Germany). Briefly, a training subset of images was annotated as “not tissue,” “unlabeled tissue,” and “rabies-labeled neuron” by a trained user to prepare a machine learning kernel to automatically identify rabies-labeled neurons. The resulting kernel was refined over several iterations until it accurately identified rabies-labeled cells. Ilastik then automatically classified each pixel in from each image based on its probability of being a member of each category. The resulting probability map for “rabies-labeled neuron” were segmented with “regionprops” in MATLAB, and the centroids of each identified neuron were identified. Automatically identified cells (Ilastik, Heidelberg University) were reviewed and confirmed by a human observer for quality control.

Each identified “input cell” was assigned to a brain region based on its anatomic location. Thus, each imaged brain was transformed into a list containing the numbers and positions of identified neurons in each brain region and for each experimental animal. The identified populations were interpreted as the afferent synaptic input to the target population. Our past successes with this strategy likely result from the following three factors: (1) thousands of input neurons are typically labeled in each brain providing broad coverage of social circuits; (2) the genetic specificity, “starter neurons,” of our approach reduces variability introduced by cell-type heterogeneity and by fibers of passage at the injection site; and (3) the automated analysis pipeline allows the user to annotate a sample dataset, which is then used to efficiently and accurately segment all subsequent datasets into input cells, starter cells, and unlabeled tissue in an unbiased manner (Menegas et al., 2015). Because of the intensely bright fluorescence of EGFP driven by the EnvA-SADΔG-EGFP virus, this algorithm can identify rabies-labeled neurons with >99% accuracy and with very few false positives.

### Brain alignment

Each reconstructed 3D brain was aligned to the common framework Allen Brain Atlas (ccfv3; Wang et al., 2020b) using elastix (Klein et al., 2010; Shamonin et al., 2014). Custom MATLAB scripts (MathWorks) were used to integrate user input and computer processing and produce the alignment. Transformix (Klein et al., 2010; Shamonin et al., 2014) was then used to migrate the identified cells in each brain to the position of the accurate brain region in the standard Allen Brain Atlas framework. This allowed us to identify the number of EGFP-expressing cells in each region of the brain in each animal.

### Statistical analysis

Statistical analyses were performed using MATLAB. Means are reported with SEM. Regressions were performed in MATLAB using the “fitlm” and “predict” functions. Regions identified as providing input to MeA arom^+^ neurons were determined using a single-sample *t* test on the observed cell counts in each region with Bonferroni’s correction for multiple comparisons when appropriate. A second comparison was made using a single-sample *t* test to determine whether the input from a given region exceeded the expected input based on chance using the region volume and an even distribution of cells as the chance rates (Extended Data Table 3-1). Sex differences in synaptic input were first characterized using a repeated-measures ANOVA with interactions conducted in MATLAB. *Post hoc* analyses for sex differences were conducted using an exact permutation test comparing the cell counts observed in males to those in females for each region (Efron and Tibshirani, 1986). The *d*′ value was calculated as a sensitivity index for distributions with different SEM values by normalizing the difference in cell count means by the SD of the overall distribution. All scripts required to replicate these analyses have been provided as supplementary materials.

### Data availability

These data have been uploaded to ebrains online repository (reference #17110259) for future access and to allow reproducibility.

## Results

**Figure 1.**
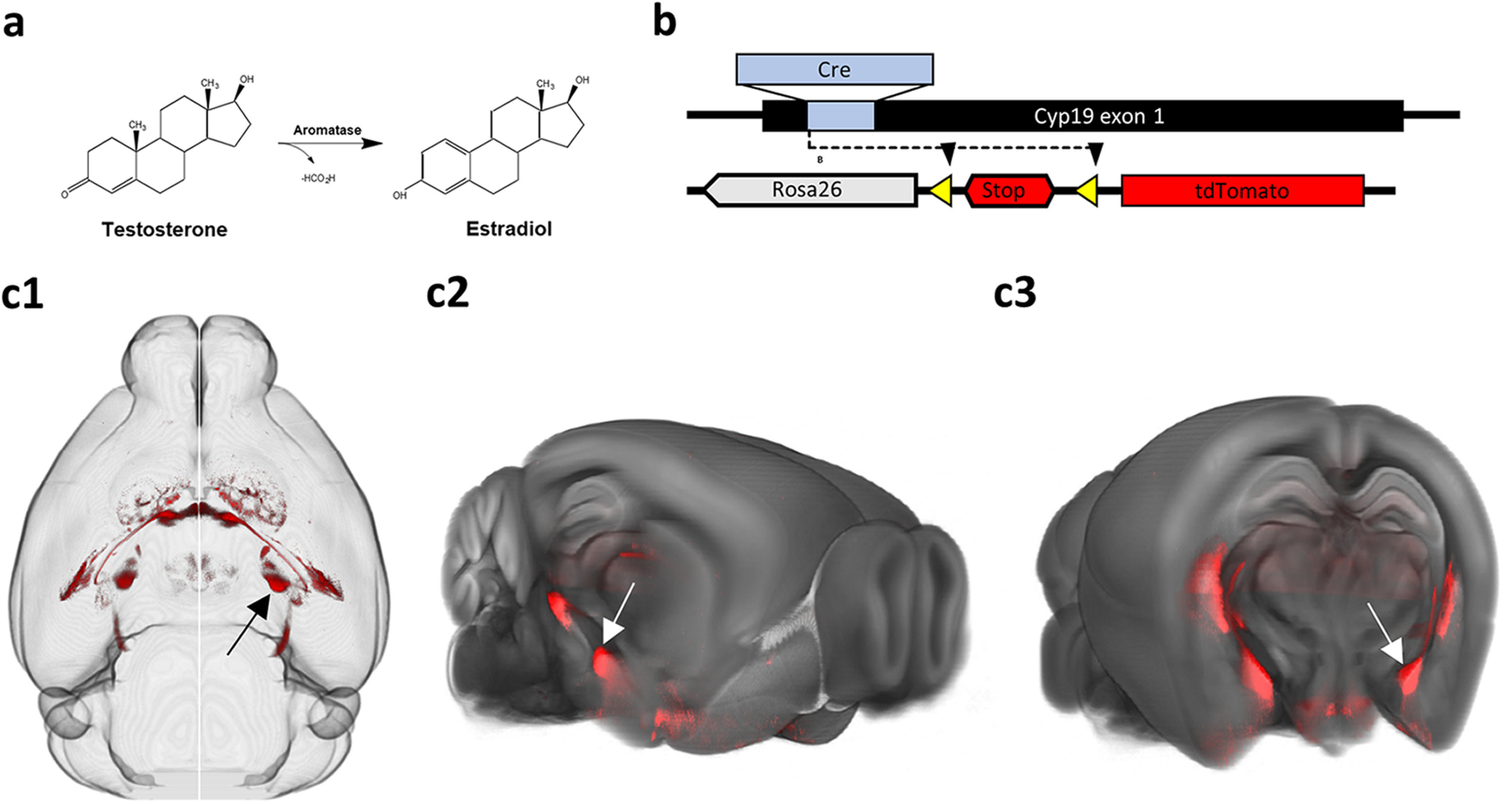
Identifying aromatase-expressing (arom^+^) neurons in the mouse brain. ***a***, The aromatase enzyme mediates the conversion of testosterone into estradiol. ***b***, Crossing aromatase-cre and Ai9 tdTomato reporter mice ensures that the tdTomato fluorescent protein is coexpressed with aromatase. ***c***, A large population of aromatase-expressing cells is present in the posterodorsal MeA [arrow in (1) horizontal, (2) sagittal, and (3) coronal view].

We used a previously described aromatase-cre transgenic mouse line (Yao et al., 2017) crossed with the AI9 tdTomato reporter line (Madisen et al., 2010) to identify aromatase-expressing neurons throughout the brain (Fig. 1). Consistent with previous reports, aromatase was expressed in a subset of cells largely restricted to the social behavior network with the largest population located in the posterodorsal MeA (MeApd; Fig. 1*C*; Newman, 1999; Morris et al., 2008b; Unger et al., 2015; Yao et al., 2017). We cleared the intact mouse brain with a modified version of CLARITY (Chung et al., 2013; Dwyer et al., 2021), and imaged the whole brain using light sheet microscopy (Menegas et al., 2015; Isogai et al., 2017). The full brain was digitally reconstructed and aligned to the Allen Brain Atlas reference brain to allow automated categorization by region. This approach allowed us to identify labeled neurons throughout the brain and to maintain the macroscopic organization of these cells (Fig. 1*C*).

### Population data: broad-scale brain regions

**Figure 2.**
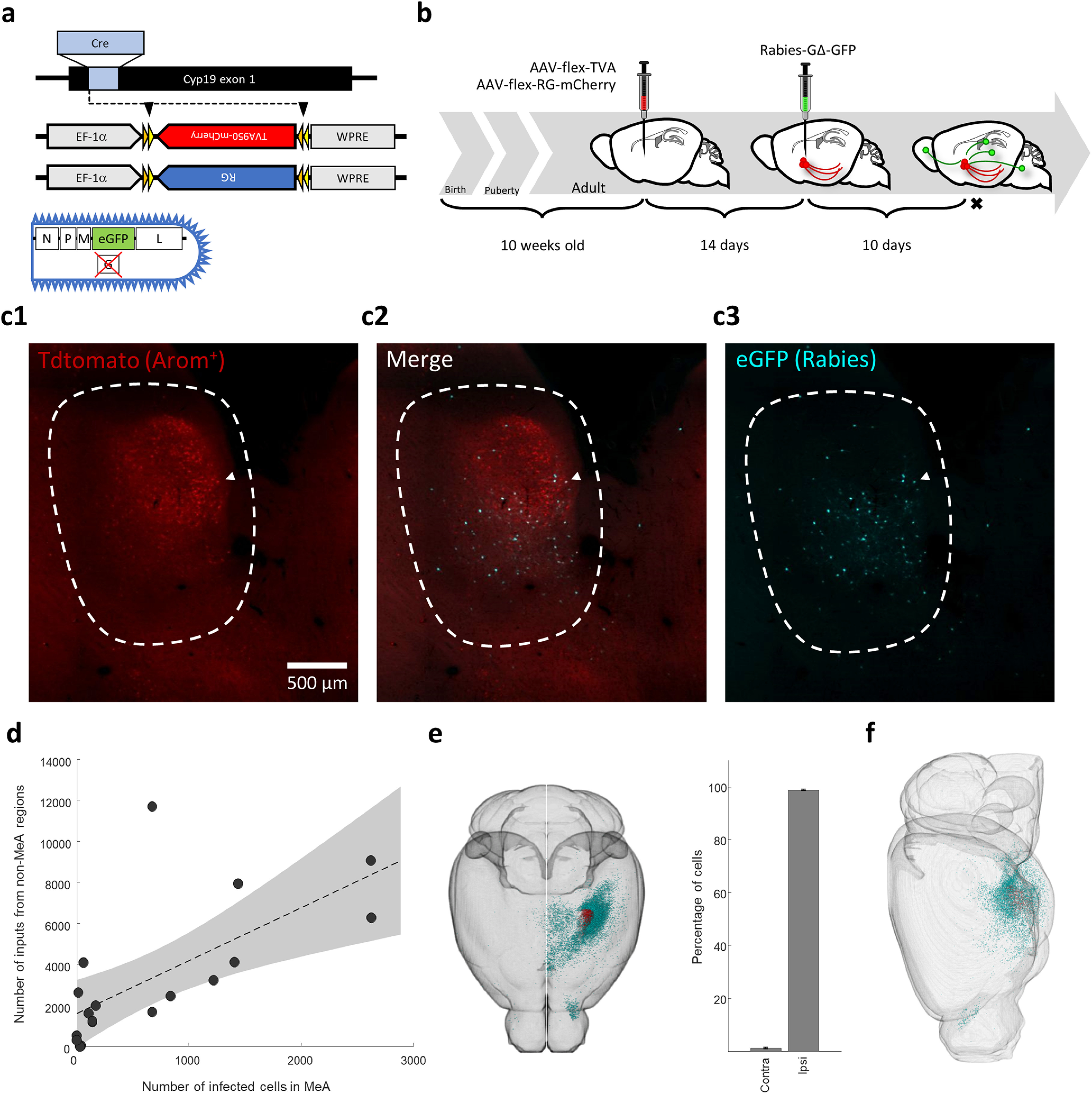
Mapping the inputome of arom^+^ neurons in the MeA. ***a***, Conditional AAV vectors were injected in aromatase-cre mice to express TVA, RG, and mCherry in arom^+^ neurons of the MeA. ***b***, Fourteen days after AAV injection, G-deleted rabies (GFP) was injected at the same stereotaxic coordinates to infect arom^+^ starter neurons; followed by 10 d for retrograde movement of rabies from arom^+^ neurons in the MeA to upstream neurons. ***c***, Infected starter neurons in the MeApd identified by simultaneous tdtomato and EGFP expression. ***d***, The number of neurons labeled with GFP by rabies infection outside the MeA scales linearly with the number of starter neurons infected in the MeApd. ***e***, View through the dorsal aspect of the brain showing location of starter neurons (red) and inputome (green), demonstrating that input cells are almost entirely ipsilateral to the injection site (see graph). ***f***, Lateral view of the same cells shown in panel ***e*** showing that most inputs originate from subcortical regions.

Next, we identified the inputome for aromatase-expressing neurons located in the MeApd. The MeApd of adult male and female aromatase-cre mice was sterotaxically injected with double-floxed inverse orientation AAVs that selectively express the EnvA receptor (TVA) and RG in aromatase-expressing neurons following cre-based recombination (Fig. 2*A*; see Materials and Methods). Two weeks later, an avian G-deleted rabies virus was injected in the same location (Rabies-ΔG-EGFP). Endogenous tdtomato and EGFP-expressing starter neurons were visible in the MeApd (Fig. 2*C*). As above, rabies-infected brains were cleared, imaged with light sheet microscopy, and aligned to the Allen reference atlas. GFP^+^ neurons were automatically detected using a machine vision algorithm and manually curated to ensure accuracy (see Materials and Methods; Menegas et al., 2015).

The number of neurons retrogradely labeled by rabies, which synapse directly on aromatase-expressing neurons in the MeApd (→MeA^arom+^), were counted in all brain regions. The number of neurons labeled outside the MeA scaled linearly with the size of the initially infected starter neuron population in the MeApd (Fig. 2*D*; *B* = 2.6; 95% CI, 1.06, 4.1; *R*^2^ = 0.45; *F* = 12.9, *p* = 0.002). Injections were performed unilaterally in either the right or left hemisphere, and the vast majority of →MeA^arom+^ neurons were ipsilateral, indicating that the circuit that provides input to aromatase-expressing neurons in the MeA is largely contained within a single hemisphere (Fig. 2*E*, Table 1-1; mean ± SEM ipsilateral cells = 98.9 ± 0.24%; *t* test: *p* = 0.0002). Consistent with previous descriptions of inputs to the MeA (Scalia and Winans, 1975), almost the entire identified inputome was restricted to subcortical regions (99.6; Fig. 2*F*). Even when looking at finer regions that span the brain (e.g., hippocampal formation), we find nearly all cells confined to the most ventral areas (see Fig. 7*A*). Together, we found that each starter neuron is associated with >100 presynaptic neurons on average, and the overwhelming majority of these neurons were both ipsilateral and subcortical.

10.1523/ENEURO.0329-21.2021.t1-1 Table 1-1 Ipsilateral bias across brain regions. Download Table 1-1, XLSX file.

### Synaptic input: coarse regions

**Figure 3.**
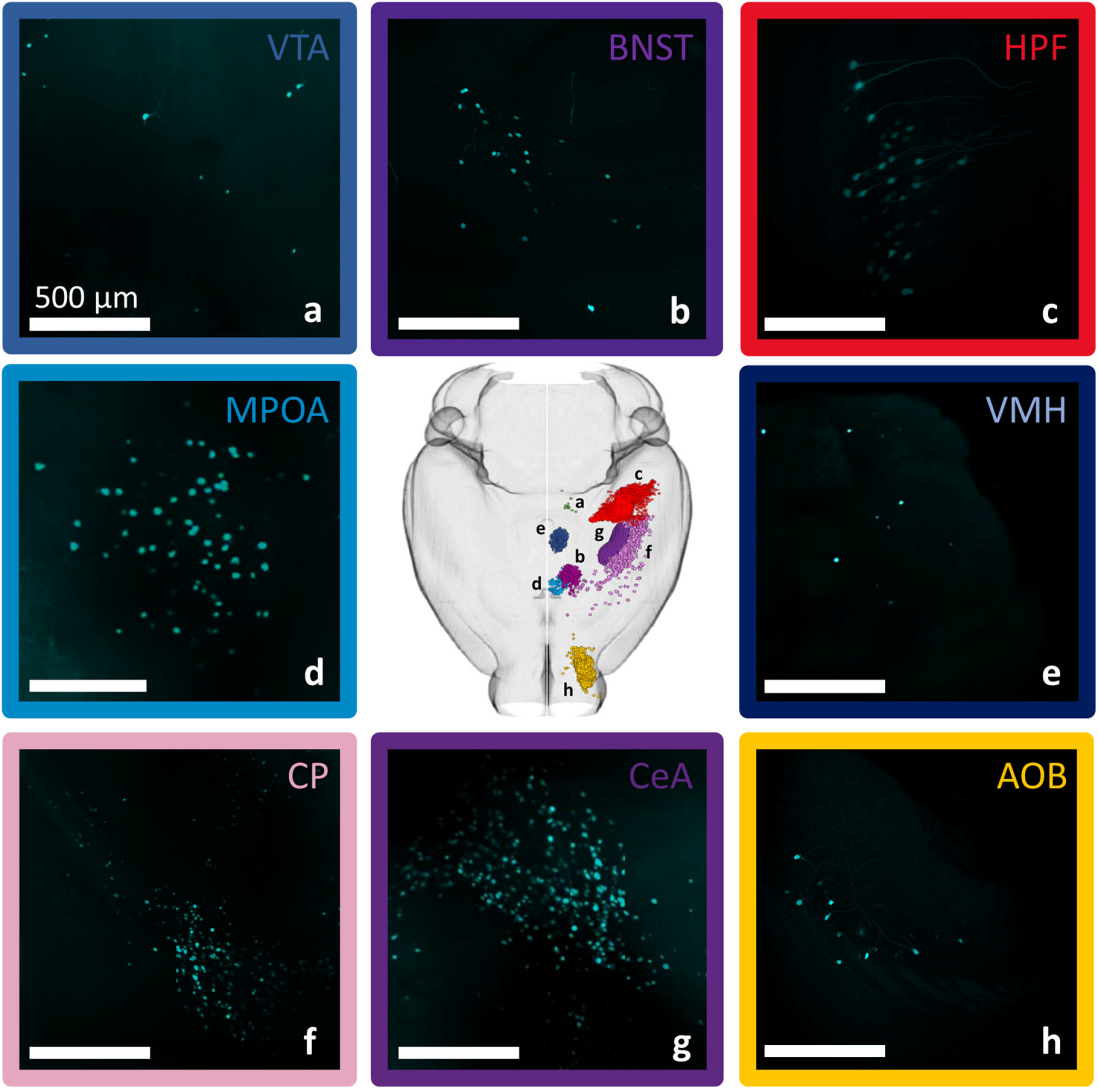
Input cell numbers vary across subcortical brain regions. A sampling of input cells observed in one individual after alignment to the Allen mouse brain common coordinate framework demonstrates that inputs can be localized to subcortical regions brain-wide and that input cell density varies among brain regions. ***a***, ventral tegmental area (VTA). ***b***, bed nucleus of the stria teminalis (BNST). ***c***, hippocampal formation (HPF). ***d***, medial preoptic area (MPOA). ***e***, ventromedial hypothalamus (VMH). ***f***, caudoputamen (CP). ***g***, central amygdala (CeA). ***h***, accessory olfactory bulb (AOB). Scale bars, 500 μm.

**Figure 4.**
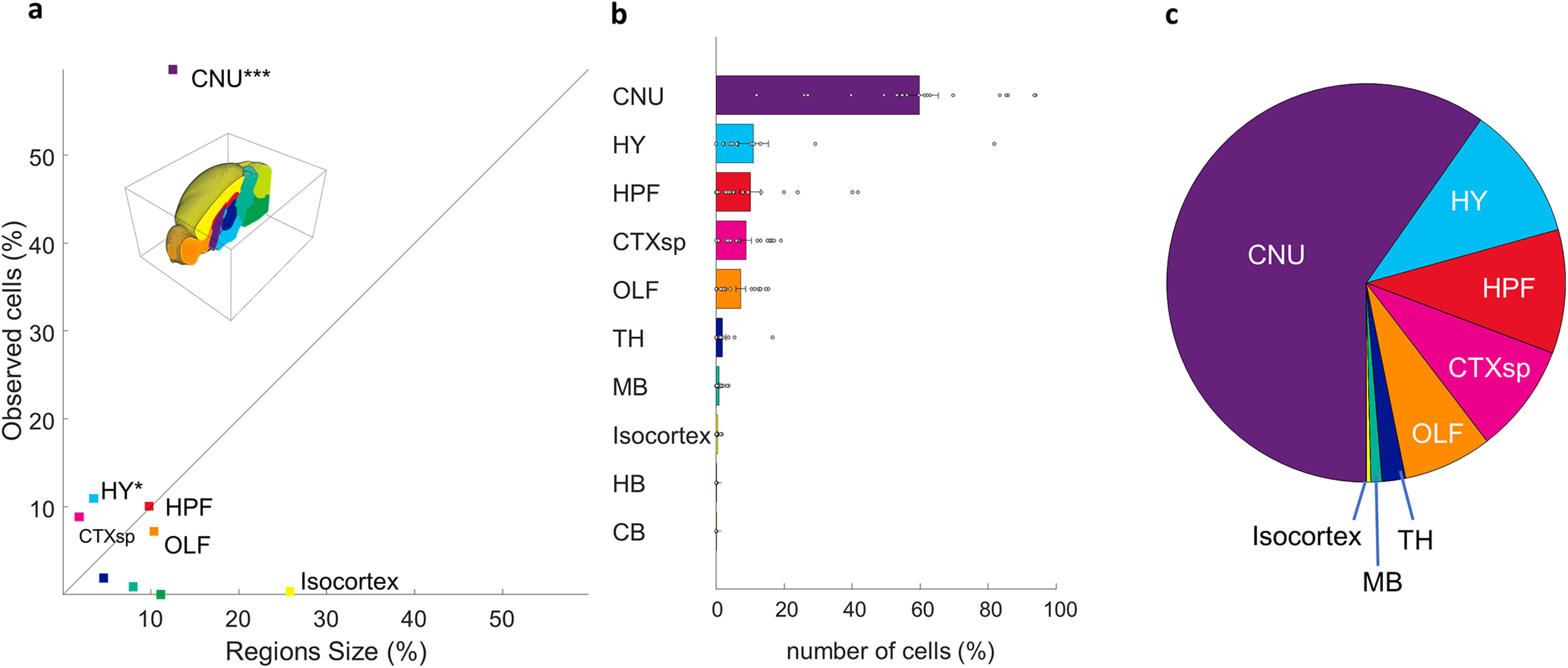
Coarse population count of the MeA arom^+^ inputome. Each input cell was assigned to 1 of 10 nonoverlapping regions spanning the full volume of the reference brain. Defined regions are as follows: cerebral nuclei (CNU), hypothalamus (H), hippocampal formation (HPF), cortical subplate (CTXsp), olfactory areas (OF), thalamus (TH), midbrain (MB), isocortex, hindbrain (HB), and cerebellum (CB). ***a***, Graph showing the percentage of the average total observed input neurons found in each brain region relative to the volume of that brain region shows that some regions have either much higher or much lower density of inputs than would be expected if the cells were evenly distributed across the brain (straight line). ***b***, Percentages of the average total number of input cells found in each defined brain region. ***c***, Pie chart illustrating the proportions of inputome cells found in eight brain regions. Extended Data Figures 4-1, 4-2, 4-3, and 4-4 support Figure 4.

Aligning our whole brain datasets to the Allen reference atlas allowed us to identify the location of each neuron with high specificity. An individual dataset typically had neurons distributed across many subcortical regions. An example of neurons identified in a single animal is seen in Figure 3. We divided the Allen atlas into 10 mutually exclusive brain regions spanning the full volume of the reference brain and used these regions to coarsely classify the inputome for aromatase-expressing MeApd neurons (Fig. 4*A*,*B*; see Materials and Methods). Only statistically significant sources of synaptic input are reported, unless otherwise noted. The majority of →MeA^arom+^ were located in the cerebral nuclei (59.7 ± 1.4; *t*_(17)_ = 10.8, *p* < 0.0001), with additional significant populations of →MeA^arom+^ neurons in the hypothalamus (11 ± 1.1; *t*_(17)_ = 2.5, *p* = 0.01), hippocampus (10 ± 0.8; *t*_(17)_ = 3.26, *p* = 0.0023), cortical subplate (8.9 ± 0.4; *t*_(17)_ = 5.7, *p* < 0.0001), and olfactory regions (7.2 ± 0.35; *t*_(17)_ = 5.2, *p* < 0.0001; Fig. 4*B*,*C*). A small but consistent population of →MeA^arom+^ neurons was identified in the thalamus (1.9 ± 0.2; *t*_(17)_ = 2.05, *p* = 0.03), and midbrain (0.9 ± 0.07; *t*_(17)_ = 3.4, *p* = 0.0016). Very few neurons were identified in the isocortex (0.36 ± 0.03; *t*_(17)_ = 3.3, *p* = 0.002), although the isocortex and cerebellum represent the two largest regions of the brain by volume. Inputs from the hindbrain (0.02 ± 0.003; *t*_(17)_ = 1.5, *p* = 0.076) and cerebellum (0.009 ± 0.002; *t*_(17)_ = 1, *p* = 0.17) did not reach statistical significance. This indicates that the densities of neurons in the isocortex, cerebellum, midbrain, and hindbrain are very low, while the densities of labeled neurons was highest in the cerebral nuclei, cortical subplate, and hypothalamus (Fig. 4*A*, Extended Data Fig. 4-1). Because the midbrain, isocortex, hindbrain, and cerebellum each contained less than a single percent of the →MeA^arom+^, they were excluded from the main dataset. We have included a detailed summary of all inputs from these minor regions in the supplementary materials (Extended Data Figs. 4-2, 4-3, 4-4, Table 2-1). Comparison of the cell counts to cell density is included (Extended Data Figs. 4-1, 5-1, 6-1, 7-1, 8-1).

10.1523/ENEURO.0329-21.2021.f4-1 Figure 4-1 Input cell density in regions brain wide. Percentage of input cells in each coarse region identified in the Allen Brain Atlas normalized to the volume of that region to determine cell densities in each region are presented as graph (***a***) and pie chart (***b***). The densest concentration of input cells is found in the cortical subplate (CTXsp). Download Figure 4-1, TIF file.

10.1523/ENEURO.0329-21.2021.f4-2 Figure 4-2 MeA arom^+^ inputome originating in the midbrain (MB). ***a***, Bar graph depicts the percentages of input cells found in major divisions of the midbrain relative to the whole-brain inputome; and pie chart shows the relative proportion of those cells within those regions. Regions shown: motor-related midbrain regions (MBmot), sensory-related midbrain regions (MBsec), and behavior-related midbrain regions (MBsta). ***b***, Inputs from motor-related midbrain regions: bar graph depicts the percentages of input cells found in major divisions of these regions relative to the whole-brain inputome; and pie chart shows the relative proportion of input cells within those regions. Regions shown: midbrain reticular nucleus (MRN), superior colliculus—motor (SCm), VTA, substantia nigra (SNr), periaqueductal gray (PAG), lateral terminal nucleus of accessory optic tract (LT), cuneiform nucleus (CUN), red nucleus (RN), retrorubal area of midbrain reticular nucleus (RR), paratrochlear nucleus (Pa4), pretectal region (PRT), medial terminal nucleus of accessory optic tract (MT), dorsal terminal nucleus of accessory optic tract (DT), anterior tegmental nucleus (AT), ventral tegmental nucleus (VTN), trochlear nucleus (IV), Edinger-Westphal nucleus (EW), medial accessory oculomotor nucleus (MA3), oculomotor nucleus (III), and paranigral nucleus (PN). ***c***, Inputs from sensory-related midbrain regions: bar graph depicts the percentages of input cells found in major divisions of these regions relative to the whole-brain inputome; and pie chart shows the relative proportion of input cells within those regions. Regions shown: inferior colliculus (IC), brachium of inferior colliculus (NB), subcommissural organ (SCO), midbrain trigeminal nucleus (MEV), parabigeminal nucleus (PBG), nucleus sagulum (SAG), and superior colliculus (sensory; SCs). **Less than 0.01% of MeA arom^+^ inputs originate in behavior-related midbrain area; most of those cells are found in the dorsal raphe nucleus (DR), pedunculopontine nucleus (PPN), and interpeduncular nucleus (IPN). Download Figure 4-2, TIF file.

10.1523/ENEURO.0329-21.2021.f4-3 Figure 4-3 MeA arom^+^ inputome originating in the hindbrain (HB). ***a***, Whole hindbrain: bar graph depicts the percentages of input cells found in major divisions of the hindbrain relative to the whole-brain inputome; and pie chart shows the relative proportion of input cells within those regions. Regions shown: motor-related medulla (MY-mot), sensory-related medulla (MY-sen), behavioral state medulla (MY-sat), motor-related pons (P-mot), sensory-related pons (P-sen), and behavioral state pons (P-sat). ***b***, Only one of the behavior-state portions of the medulla, the nucleus raphe magnus (RM) contains inputs to arom^+^ cells in the MeA. ***c***, Bar graph depicts the percentages of input cells found in major divisions of the motor-related medulla relative to the whole-brain inputome; and pie chart shows the relative proportion of input cells within those regions. Two regions of the motor-related medulla provide inputs: the gigantocellular reticular nucleus (GRN) and the facial motor nucleus (VII). ***d***, The caudal part of the pontine reticular nucleus (PRNc) is the only part of the motor-related pons to provide inputs to arom^+^ MeA cells. ***e***, In the sensory-related pons, inputs originate from the parabrachial nucleus (PB) and the nucleus of the lateral lemniscus (NLL). Download Figure 4-3, TIF file.

10.1523/ENEURO.0329-21.2021.f4-4 Figure 4-4 MeA arom^+^ inputome originating in the isocortex and the cerebellum (CB). ***a***, Bar graph depicts the percentages of input cells found in major divisions of the isocortex relative to the whole-brain inputome; and pie chart shows the relative proportion of input cells within those regions. Regions shown: Secondary motor area (MOs), agranular insular area (AI), orbital area (ORB), temporal association area (Tea), ectorhinal area (ECT), visceral area (VISC), anterior cingulate area (ACA), infralimbic area (ILA), primary motor area (MOp), supplementary somatosensory area (SSs), gustatory areas (GU), auditory areas (AUD), visual areas (VIS), primary somatosensory area (SSp), prelimbic area (PL), perirhinal area (PERI), posterior parietal association areas (PTLps), and retrosplenial area (RSP). ***b***, Inputs from the cerebellum are only found in the cerebellar cortex (CBX). Download Figure 4-4, TIF file.

**Figure 5.**
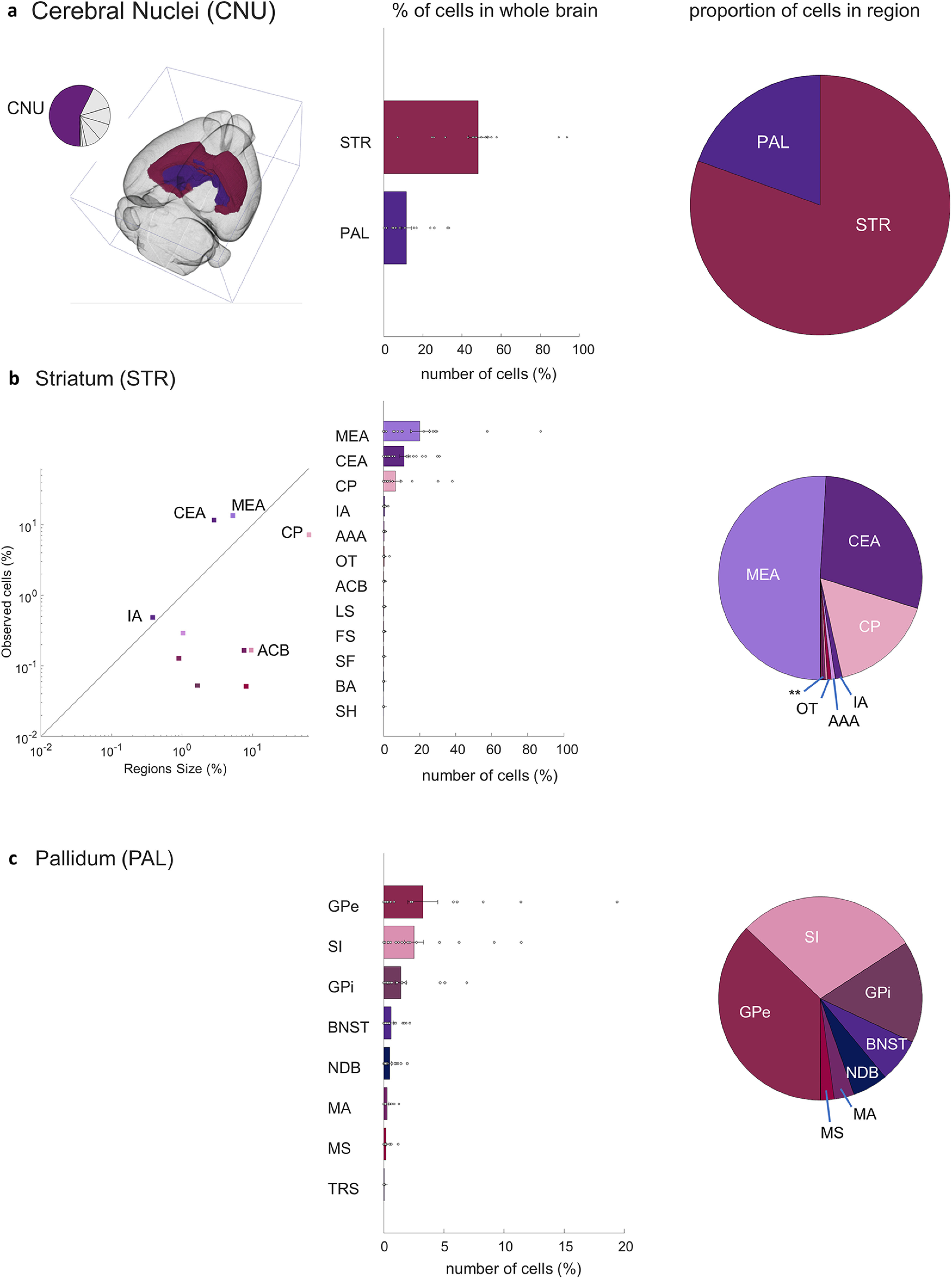
MeA arom^+^ inputome originating in the cerebral nuclei (CNU). ***a***, Left, A pie chart indicates the fraction of all labeled neurons in the CNU and the brain atlas depicts the position of CNU regions. Right, Bar graph depicts the percentages of input cells found in the striatum (STR) and pallidum (PAL) relative to the whole-brain inputome; pie chart shows the relative proportion of those cells within the cerebral nuclei alone. ***b***, Locations of input neurons within the striatum: line graph shows relative density of cells in subregions of the striatum; bar graph shows relative distribution of inputs relative to the whole-brain inputome; pie chart shows the relative proportion of those cells within parts of the striatum. Regions shown: medial amygdala (MEA), central amygdala (CEA), caudoputamen (CP), intercalated amygdalar nucleus (IA), anterior amygdalar area (AAA), nucleus accumbens (ACB), lateral septal nucleus (LS), fundus of striatum (FS), olfactory tubercle (OT), septofimbrial nucleus (SF), bed nucleus of the accessory olfactory tract (BA), and septohippocampal nucleus (SH). ***c***, Locations of input neurons within the pallidum: bar graph shows relative distribution of inputs relative to the whole-brain inputome; pie chart shows the relative proportion of those cells within parts of the pallidum. Regions shown: external globus pallidus (GPe), substantia innominata (SI), internal globus pallidus (GPi), BNST, diagonal band nucleus (NDB), magnocellular nucleus (MA), medial septal nucleus (MS), and triangular nucleus of septum (TRS). Extended Data Figure 5-1 supports Figure 5.

10.1523/ENEURO.0329-21.2021.f5-1 Figure 5-1 Input cell density in the cerebral nuclei (CNU). ***a***, Input cell densities of constituent regions of the cerebral nuclei. ***b***, Input cell densities of constituent regions in the striatum. ***c***, Input cell densities of constituent regions in the pallidum. Results are presented as both bar chart and pie charts. Download Figure 5-1, TIF file.

**Figure 6.**
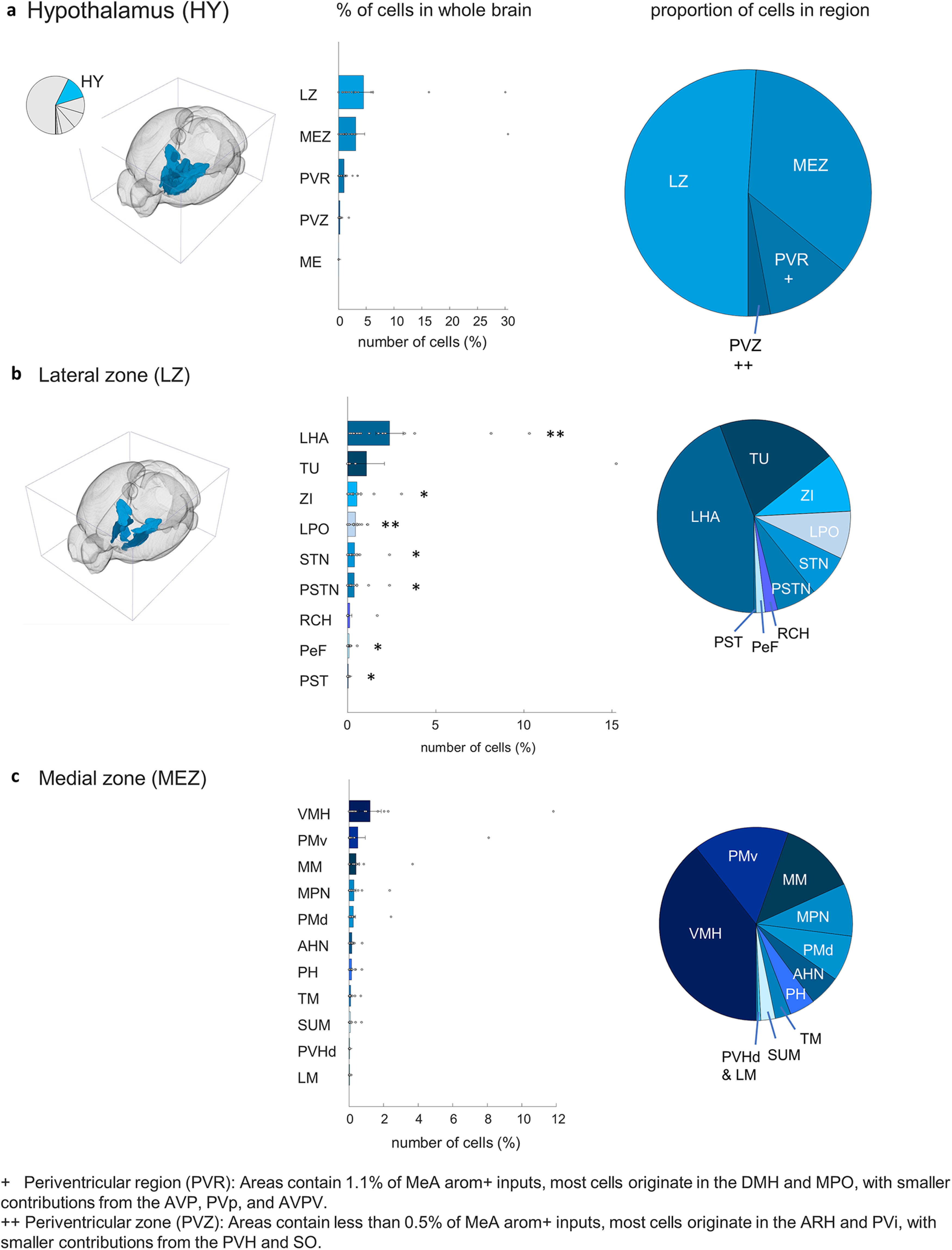
MeA arom^+^ inputome originating in the hypothalamus (HY). ***a***, Left, A pie chart indicates the fraction of all labeled neurons in the hypothalamus, and the brain atlas depicts the position of hypothalamic regions. Right, Bar graph depicts the percentages of input cells found in the five subregions of the hypothalamus relative to the whole-brain inputome; pie chart shows the relative proportion of those cells within those hypothalamic regions. Regions shown: lateral zone (LZ), medial zone (MEZ), periventricular region (PVR), periventricular zone (PVZ), and median eminence (ME). ***b***, Locations of input neurons within the lateral zone of the hypothalamus: bar graph shows relative distribution of inputs relative to the whole-brain inputome; and pie chart shows the relative proportion of those cells within parts of the medial zone. Regions shown: lateral hypothalamic area (LHA), Tuberal nucleus (TU), zona incerta (ZI), lateral preoptic area (LPO), subthalamic nucleus (STN), parasubthalamic nucleus (PSTN), retrochiasmatic area (RCH), perifornical nucleus (PeF), and preparasubthalamic nucleus (PST). ***c***, Locations of input neurons within the medial zone of the hypothalamus: bar graph shows relative distribution of inputs relative to the whole-brain inputome; pie chart shows the relative proportion of those cells within parts of the medial zone. Regions shown: ventromedial hypothalamic nucleus (VMH), PMv, medial mammillary nucleus (MM), medial preoptic nucleus (MPN), dorsal premammillary nucleus (PMd), anterior hypothalamic nucleus (AHN), posterior hypothalamic nucleus (PH), tuberomammillary nucleus (TM), supramammillary nucleus (SUM), paraventricular hypothalamic nucleus (PVHd), and lateral mammillary nucleus (LM). Extended Data Figure 6-1 supports Figure 6.

10.1523/ENEURO.0329-21.2021.f6-1 Figure 6-1 Input cell density in the hypothalamus (HY). ***a***, Input cell densities of constituent regions in the hypothalamus. ***b***, Input cell densities of constituent regions in the lateral zone of the hypothalamus. ***c***, Input cell densities of constituent regions in the medial zone of the hypothalamus. Results are presented as both bar chart and pie charts. Download Figure 6-1, TIF file.

**Figure 7.**
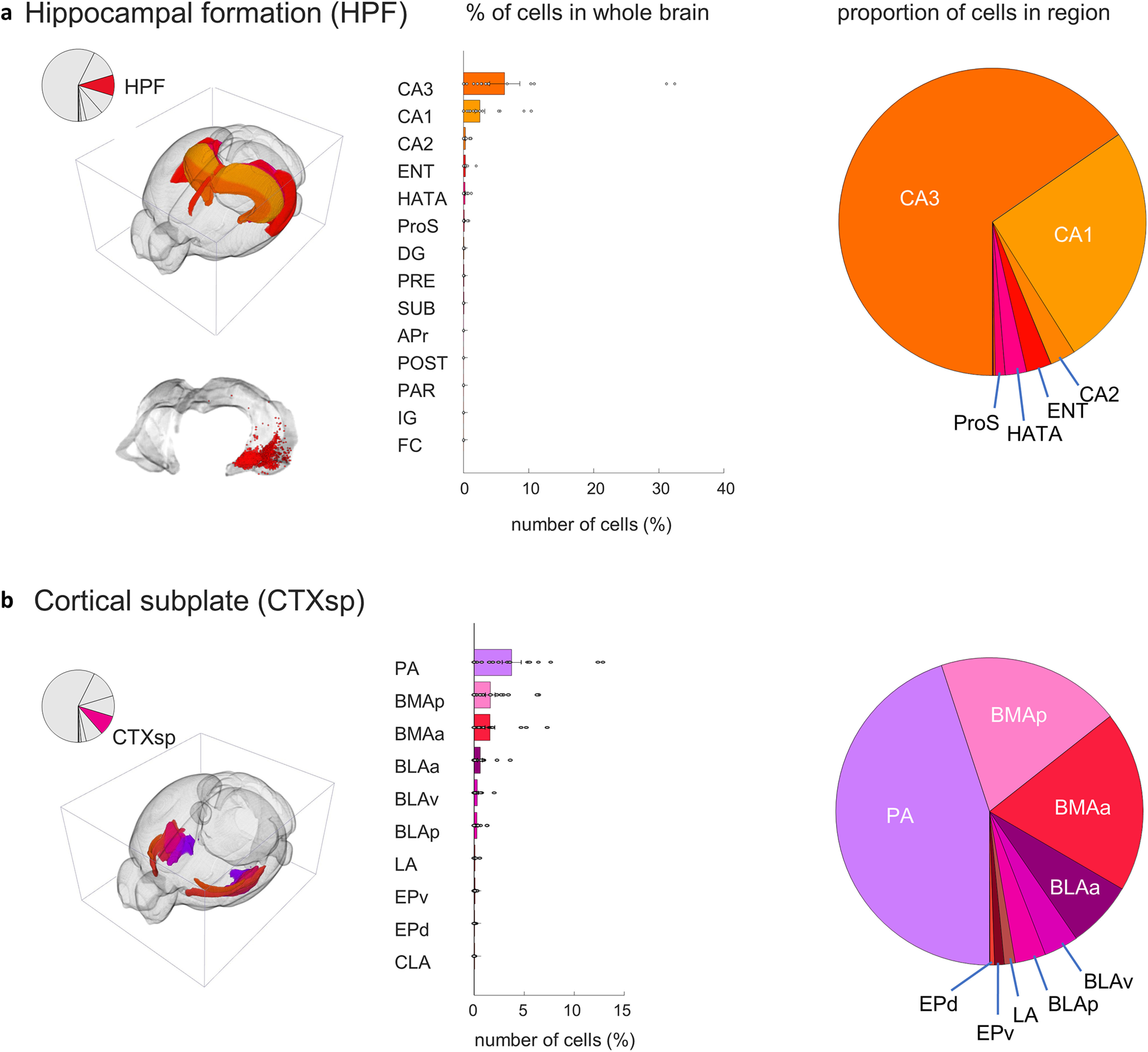
MeA arom^+^ inputome originating in the hippocampal formation (HPF) and the cortical subplate (CTXsp). ***a***, Left, A pie chart indicates the fraction of all labeled neurons in the hippocampus and the brain atlas depicts the position of hippocampal regions. Right, Bar graph depicts the percentages of input cells found in the regions of the hippocampal formation relative to the whole-brain inputome; and pie chart shows the relative proportion of those cells within those regions. The lower inset image illustrates that input cells are located primarily in ventral portions of the HPF. Regions shown: field CA1 (CA1), field CA2 (CA2), field CA3 (CA3), entorhinal area (ENT), hippocampal-amygdalar transition area (HATA), prosubiculum (ProS), dentate gyrus (DG), presubiculum (PRE), subiculum (SUB), area prostriata (APr), postsubiculum (POST), parasubiculum (PAR), induseum griseum (IG), and fasciola cinerea (FC). ***b***, Left, A pie chart indicates the fraction of all labeled neurons in the cortical subplate, and the brain atlas depicts the position of cortical subplate regions. Right, Bar graph depicts the percentages of input cells found in the regions of the cortical subplate relative to the whole-brain inputome; and pie chart shows the relative proportion of those cells within those regions. Regions shown: posterior amygdalar nucleus (PA), anterior BMA (BMAa), posterior BMA (BMAp), anterior BMA (BLAa), ventral basolateral amygdalar nucleus (BLAv), posterior basolateral amygdalar nucleus (BLAp), lateral amygdalar nucleus (LA), ventral endopiriform nucleus (EPv), dorsal endopiriform nucleus (EPd), and claustrum (CLA). Extended Data Figure 7-1 supports Figure 7.

10.1523/ENEURO.0329-21.2021.f7-1 Figure 7-1 Input cell density in the hippocampal formation (HPF) and the cortical subplate (CTXsp). ***a***, Input cell densities of constituent regions in the hippocampal formation. ***b***, Input cell densities of constituent regions in the cortical subplate. Results are presented as both bar chart and pie charts. Download Figure 7-1, TIF file.

**Figure 8.**
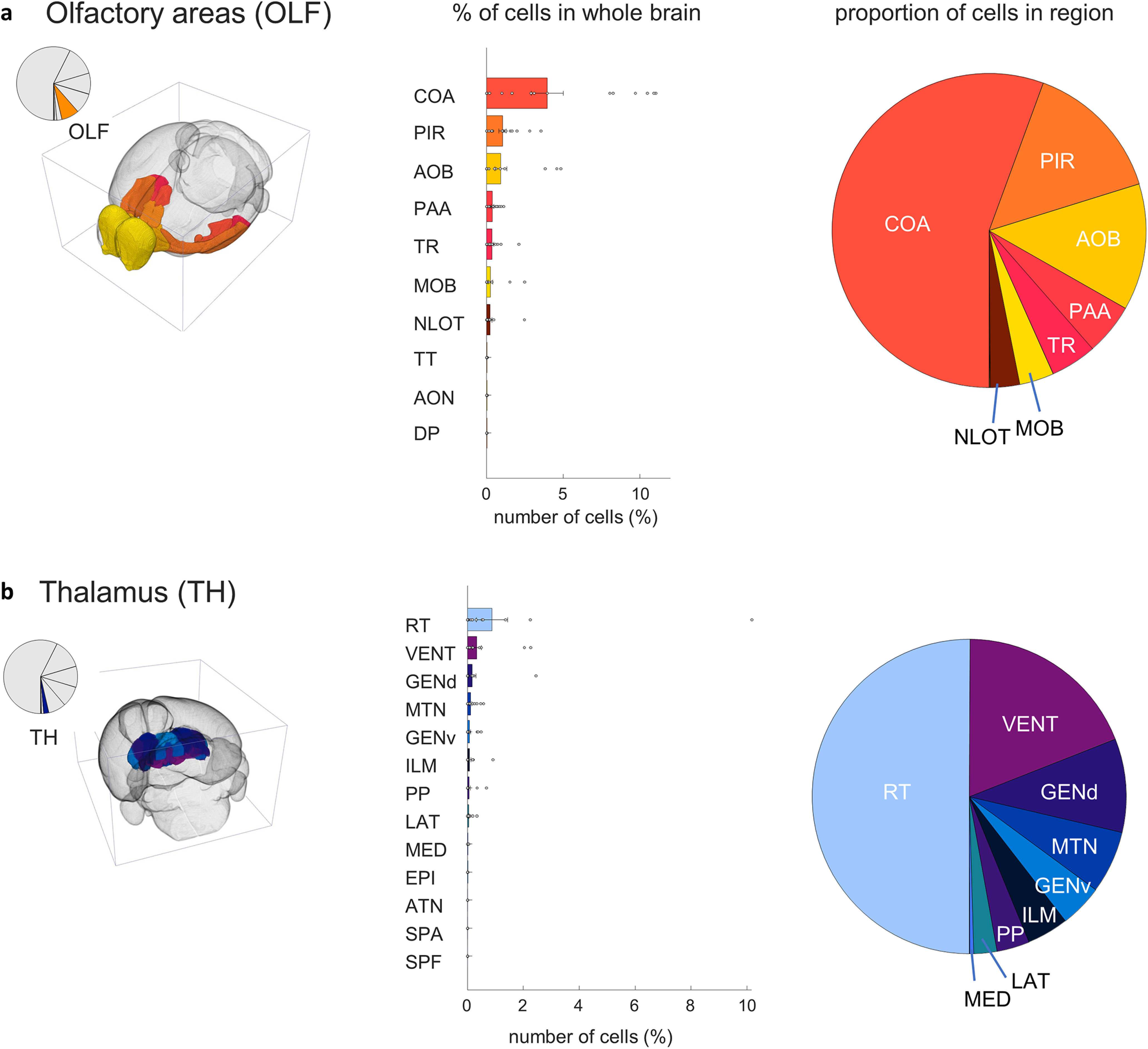
MeA arom^+^ inputome originating in the olfactory areas (OLF) and the thalamus (TH). ***a***, Left, A pie chart indicates the fraction of all labeled neurons in olfactory areas; and the brain atlas depicts the position of olfactory areas. Right, Bar graph depicts the percentages of input cells found in subregions of the olfactory area relative to the whole-brain inputome; and pie chart shows the relative proportion of those cells within those regions. Regions shown: cortical amygdalar area (COA), piriform area (PIR), accessory olfactory bulb (AOB) , piriform-amygdalar area (PAA), postpiriform transition area (TR), nucleus of the lateral olfactory tract (NLOT), main olfactory bulb (MOB), taenia tecta (TT), anterior olfactory nucleus (AON), and dorsal peduncular area (DP). ***b***, Left, A pie chart indicates the fraction of all labeled neurons in the thalamus; and brain atlas depicts the position of thalamic regions. Right, Bar graph depicts the percentages of input cells found in subregions of the thalamus relative to the whole-brain inputome; and pie chart shows the relative proportion of those cells within those regions. Regions shown: reticular nucleus (RT), ventral group of the dorsal thalamus (VENT), dorsal geniculate group (GENd), midline group of the dorsal thalamus (MTN), ventral geniculate group (GENv), intralaminar nuclei of the dorsal thalamus (ILM), peripeduncular nucleus (PP), lateral group of the dorsal thalamus (LAT), medial group of the dorsal thalamus (MED), epithalamus (EPI), anterior group of the dorsal thalamus (ATN), subparafascicular area (SPA), and subparafascicular nucleus (SPF). Extended Data Figure 8-1 supports Figure 8.

10.1523/ENEURO.0329-21.2021.f8-1 Figure 8-1 Input cell density in the olfactory areas (OLF) and the thalamus (TH). ***a***, Input cell densities of constituent regions in the olfactory areas. ***b***, Input cell densities of constituent regions in the thalamus. Results are presented as both bar chart and pie charts. Download Figure 8-1, TIF file.

10.1523/ENEURO.0329-21.2021.t2-1 Table 2-1 Hypothalamic inputs to arom+ cells in the MeA. Download Table 2-1, XLSX file.

### Synaptic input: finer-scale regions

Closer investigation of five primary sources of input to the aromatase-expressing neurons in the MeApd revealed a consistent presynaptic circuit. →MeA^arom+^ neurons in the cerebral nuclei were initially divided into the striatum (48.1 ± 1.2; *t*_(17)_ = 10, *p* < 0.0001) and pallidum (11.6 ± 0.6; *t*_(17)_ = 4.7, *p* < 0.0001; Fig. 5*A*). Within the striatum, the MeA, including the starter neuron population accounts for 20 ± 1.3% of the total →MeA^arom+^ neurons in the brain (Fig. 5*B*; *t*_(17)_ = 3.8, *p* = 0.0007). Regions that border the MeA, including central amygdala (CEA) and caudoputamen (CP), accounted for an additional 11 ± 0.6% (*t*_(17)_ = 4.7, *p* < 0.0001) and 6.6 ± 0.6% (*t*_(17)_ = 2.6, *p* = 0.01) of the overall inputome, respectively (Fig. 5*B*). Smaller populations of →MeA^arom+^ were identified in the intercalated amygdalar nucleus (IA; 0.43 ± 0.04; *t*_(17)_ = 2.7, *p* = 0.0078), nucleus accumbens (0.15 ± 0.014; *t*_(17)_ = 2.6, *p* = 0.009), fundus of the striatum (0.11 ± 0.011; *t*_(17)_ = 2.5, *p* = 0.01), lateral septal nucleus (0.15 ± 0.01; *t*_(17)_ = 3, *p* = 0.0037), and septofimbrial nucleus (0.05 ± 0.006; *t*_(17)_ = 2.0, *p* = 0.034), while the populations of →MeA^arom+^ neurons in the rest of the striatal regions were not significant (Fig. 5*D*). The largest pallidum projections include the globus pallidus, both external (3.25 ± 0.3; *t*_(17)_ = 2.6, *p* = 0.009) and internal (1.5 ± 0.12; *t*_(17)_ = 3.0, *p* = 0.004) segments; the substantia innominata (2.5 ± 0.2; *t*_(17)_ = 3.2, *p* = 0.0025); the bed nuclei of the stria terminalis (0.61 ± 0.04; *t*_(17)_ = 3.5, *p* = 0.001); and the diagonal band nucleus (0.5 ± 0.03; *t*_(17)_ = 3.6, *p* = 0.001; Fig. 5*C*).

The hypothalamus provided the second largest source of →MeA^arom+^ neurons with most inputs located in the lateral (4.5 ± 0.4; *t*_(17)_ = 2.6, *p* = 0.001) and medial (3.1 ± 0.4; *t*_(17)_ = 1.9, *p* = 0.04) zones, with a smaller contribution from the periventricular region (1.0 ± 0.06; *t*_(17)_ = 3.9, *p* = 0.0006) and the periventricular zone (0.25 ± 0.03; *t*_(17)_ = 2.4, *p* = 0.01; Fig. 6*A*). The two hypothalamic regions providing the largest number of →MeA^arom+^ neurons to the inputome are the lateral hypothalamic area (2.0 ± 0.17; *t*_(17)_ = 3.0, *p* = 0.004) and ventromedial hypothalamic nucleus (1.2 ± 0.16; *t*_(17)_ = 1.9, *p* = 0.04; Fig. 6*A*, Extended Data Table 3-1).

10.1523/ENEURO.0329-21.2021.t3-1 Table 3-1 Statistical analysis of observed input cells across brain regions. Download Table 3-1, DOCX file.

Both the amygdala and hippocampus are critical for memory and contain large populations of →MeA^arom+^ neurons. Hippocampal →MeA^arom+^ neurons are primarily located in the CA3 field (6.3 ± 0.6; *t*_(17)_ = 2.7, *p* = 0.007), followed by the CA1 field (2.5 ± 0.2; *t*_(17)_ = 3.3, *p* = 0.002) and the CA2 field (0.26 ± 0.2; *t*_(17)_ = 2.8, *p* = 0.006; Fig. 7*A*). We also observed →MeA^arom+^ cells in the hippocampal–amygdaloid transition area (0.22 ± 0.02; *t*_(17)_ = 2.7, *p* = 0.00). The hippocampus is a large structure that spans nearly the entire dorsal–ventral axis of the brain. The vast majority of →MeA^arom+^ neurons in the hippocampus were located in the posterior ventral hippocampus (Fig. 7*A*).

The largest proportion of inputs from the cortical subplate originate in amygdalar nuclei (Fig. 7*B*), including the posterior amygdalar nucleus (3.8 ± 0.2; *t*_(17)_ = 4.0, *p* = 0.0005) and lateral amygdala (0.1 ± 0.01; *t*_(17)_ = 2.3, *p* = 0.017). The anterior and posterior basomedial (1.6 ± 0.1; *t*_(17)_ = 3.3, *p* = 0.002; 1.6 ± 0.1; *t*_(17)_ = 3.4, *p* = 0.002] and the anterior and posterior basolateral (0.6 ± 0.06; *t*_(17)_ = 2.6, *p* = 0.009; 0.3 ± 0.024; *t*_(17)_ = 2.8, *p* = 0.006) nuclei of the amygdala also each showed →MeA^arom+^ cells.

Within the olfactory areas (Fig. 8*A*), the largest proportion of inputs originate in the cortical amygdalar (4.0 ± 0.3; *t*_(17)_ = 3.8, *p* = 0.0007) and piriform (1.0 ± 0.06; *t*_(17)_ = 4.4, *p* = 0.0002) areas, as well as the accessory olfactory bulb (1.0 ± 0.1; *t*_(17)_ = 2.4, *p* = 0.014). Smaller contributions originate in the piriform amygdalar area (0.38 ± 0.03; *t*_(17)_ = 4.5, *p* = 0.00,017), the postpiriform transition area (0.34 ± 0.03; *t*_(17)_ = 2.8, *p* = 0.0067).

The largest proportion of input cells originating in the thalamus (Fig. 8*B*) come from the recticular nucleus (0.9 ± 0.15; *t*_(17)_ = 1.6, *p* = 0.07) though this population was variable between individuals and not statistically significant. Additional input came from the ventral group of the dorsal thalamus (0.33 ± 0.04; *t*_(17)_ = 2.1, *p* = 0.025), with smaller contributions originating from the geniculate groups of the dorsal thalamus (0.2 ± 0.03; *t*_(17)_ = 1.2, *p* = 0.1), ventral thalamus (0.08 ± 0.01; *t*_(17)_ = 2.1, *p* = 0.023), midline group of the dorsal thalamus (0.11 ± 0.01; *t*_(17)_ = 2.7, *p* = 0.0002), and lateral group of the dorsal thalamus (0.04 ± 0.005; *t*_(17)_ = 2.0, *p* = 0.03).

### Sex differences in distribution of observed input cells

**Figure 9.**
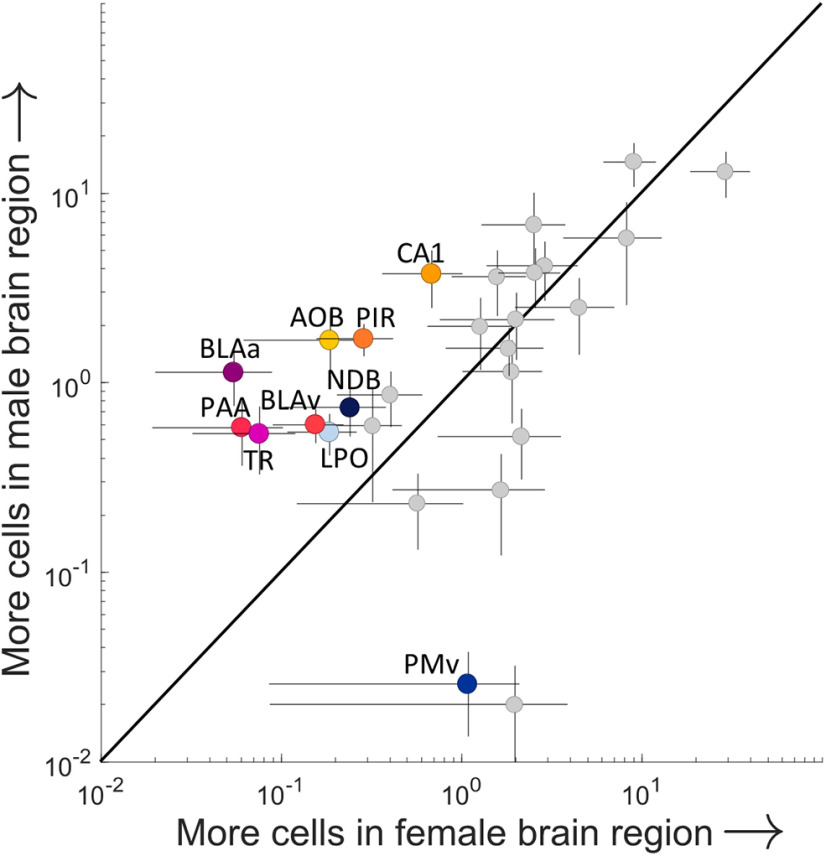
Sex differences in the inputome of aromatase-expressing cells in the MeA. The average percentage of synaptic inputs to MeA arom^+^ neurons compared for each region in males and females. Only regions that provide 0.5% of the overall input are shown, and regions identified as having a statistically significant sex difference are shown in color (*p* < 0.05, permutation *post hoc* test following ANOVA with repeated measures on “sex,” “region,” and “individual”). These regions include field CA1 (CA1), the accessory olfactory bulb (AOB), piriform area (PIR), piriform-amygdalar area (PAA), postpiriform transition area (TA), basolateral amygdalar nucleus-anterior (BLAa), basolateral amygdalar nucleus-ventral (BLAv), diagonal band nucleus (NDB), lateral preoptic area (LPO), and ventral premammillary nucleus (PMv). Regions lying above the line of unity represent a male bias, and neurons lying to the right of the unity line indicate a female bias. Error bars indicate the SEMs (male, vertical; female, horizontal) for each region. Extended Data Figure 9-1 supports Figure 9.

Because sex differences in the MeA, and specifically in MeA^arom+^ neurons, are well established (Cooke and Woolley, 2005; Yao et al., 2017; Billing et al., 2020), we investigated sex differences in synaptic input to MeA^arom+^ neurons. A regression analysis of the cell counts for all male regions against cell counts for all female regions indicated that the major regions providing synaptic input to MeA^arom+^ neurons are largely conserved across sexes (Fig. 9; *R*^2^ = 0.60 252; *F* = 39.4127; *p* < 0.00,001; linear regression). No significant difference was observed for sex when considering all regions (ANOVA: *F* = 0.04, *p* = 0.83); however, there was a significant interaction between region and sex (ANOVA: *F* = 1.81, *p* = 0.01), indicating that a subset of brain regions displayed sex differences in projections to MeA^arom+^ neurons. *Post hoc* analyses revealed nine regions that provided more input in males and one region that provided more input in females (Fig. 9). MeA^arom+^ neurons received more input from the AOB, basolateral amygdala (both anterior and ventral), CA1 region of the hippocampus, piriform areas, diagonal band nucleus, and lateral preoptic area in males and more input from the ventral premammillary nucleus (PMv) in females (*p* < 0.05, permutation test; Table 4-1). Additionally, the fraction-labeled neurons in the MeA, local to the injection site, was higher in females (29.2 ± 10.7) than males, though this trend did not reach significance (13.0 ± 3.5; *p* = 0.08, permutation test; Extended Data Fig. 9-1).

10.1523/ENEURO.0329-21.2021.f9-1 Figure 9-1 Comparison of sex differences in observed inputs to MeA^Arom+^ Cells. ***a***, Graph comparing the fraction of labeled neurons in the MeA, local to the injection site, in females and males. ***b***, graph showing the *d*′ values for sex differences in the regions presented in Figure 9. Download Figure 9-1, TIF file.

10.1523/ENEURO.0329-21.2021.t4-1 Table 4-1 Statistical analysis of sex differences in major input regions. Download Table 4-1, XLS file.

## Discussion

**Figure 10.**
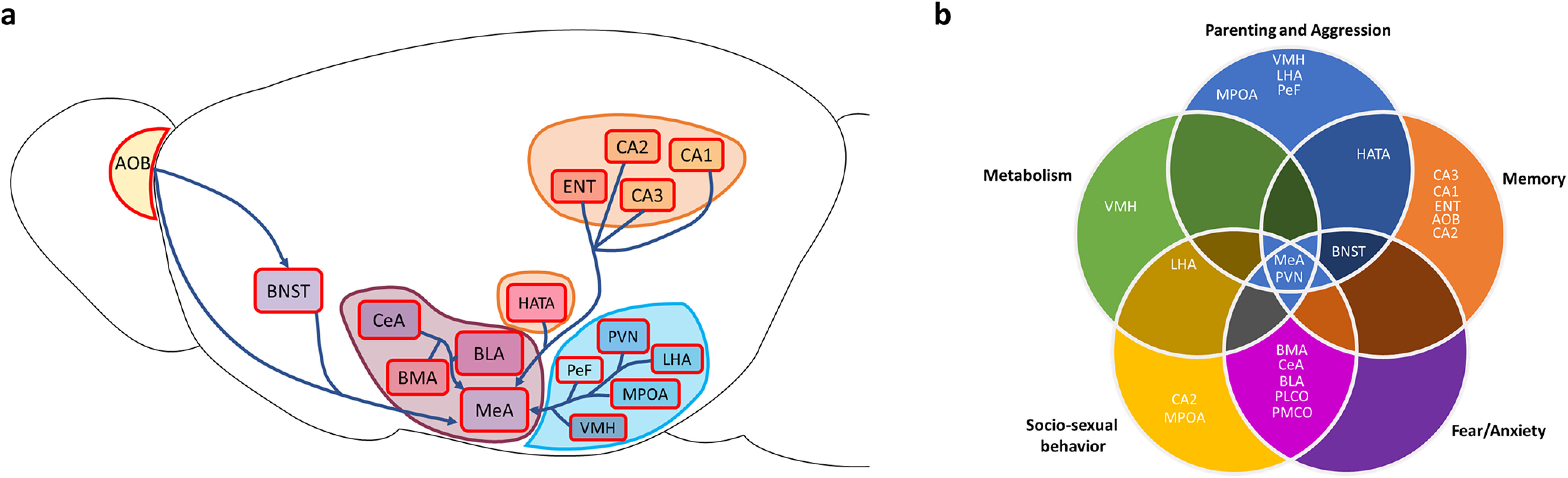
The inputome of MeA arom^+^ neurons. ***a***, Illustration showing the relative locations of the major inputs to the aromatase-expressing MeA cells throughout the brain as defined by our data. Major regions are shaded to demonstrate that the included regions were analyzed in groups and are considered components of a larger analysis region. These inputs were selected based on their relative abundance in the data and behavioral significance as represented in previous literature. ***b***, A Venn diagram showing the relative distribution of the regions displayed in ***a*** sorted by their reported roles in the production or modulation of memory, parenting and aggression, metabolism, sociosexual behaviors, and fear and anxiety behaviors.

Social behaviors require an animal to detect and respond to specific stimuli that signal the identity of a conspecific: rivals, potential mates, and offspring each demand different behavioral responses. As a central component of the social behavior network, the MeA plays an important role in interpreting chemicals used to convey social information and producing appropriate behavioral responses (Newman, 1999; Bakker et al., 2002; Yao et al., 2017). Here, we focus on a population of neurons in the MeA that express the enzyme aromatase (Naftolin et al., 1975), which has been causally linked to social behaviors (Lee et al., 2014; Unger et al., 2015; Ishii et al., 2017). In this study, we identify the broad constellation of synaptic inputs that allow aromatase-expressing MeA neurons to integrate social information. We show that these neurons receive input from an unexpectedly wide range of regions, with strong inputs from regions linked to chemosensation, memory, metabolism, and the networks underlying social behaviors and decision-making (Fig. 10).

Two regions that are classically associated with the MeA are the AOB and bed nucleus of the stria terminalis (BNST; Scalia and Winans, 1975). The MeA is often illustrated as the third node in the vomeronasal pathway: vomeronasal sensory neurons project to the AOB that in turn projects to the MeA (Scalia and Winans, 1975). Similarly, the BNST and MeA are together considered the extended amygdala, a collective region that processes sensory and social information (Di Marino et al., 2016). Using classical retrograde tracing, it has been previously shown that most AOB neurons project to the MeA (De Olmos et al., 1978; Bergan et al., 2014) with aromatase neurons receiving input overwhelmingly from the anterior subdivision of the AOB (Billing et al., 2020). Our tracing results demonstrate a clear input to aromatase-expressing MeA neurons from both the AOB and the BNST. However, the collective inputs from the AOB and BNST account for <2% of the overall →MeA^arom+^ neurons. This result should not be interpreted as evidence that few AOB neurons project to the MeA, but rather, it highlights the larger set of projections coming from areas other than the AOB and BNST. Aromatase-expressing neurons clearly have a wide-ranging role as integrators of information from inputs throughout the brain, illustrating the danger in thinking of the social behavior network as a simple feedforward network. Moreover, our results indicate that many known postsynaptic targets of the MeA are also presynaptic inputs to aromatase-expressing MeA neurons, suggesting that the MeA forms feedback loops with many regions in the social behavior network, even at the level of the population of aromatase-expressing neurons.

Nearly all →MeA^arom+^ neurons were located ipsilateral to the site of injection (Fig. 1). This is consistent with previous findings (Canteras et al., 1995; Bressler and Baum, 1996; Bergan et al., 2014) and indicates that afferent sensory information from the left and right VNO is kept separate at least to the level of the MeA. The nearly complete ipsilateral bias seems surprising given the need to integrate both sides of the body in social behavior. One possibility is that chemical compounds activate each VNO similarly, reducing the need for integration between left and right sides. A second possibility is that, like the auditory system (Schnupp and Carr, 2009; Clemens et al., 2015) or visual system (Krapp et al., 2001), internasal comparisons provide an additional line of sensory information (e.g., spatial location). In the latter case, our data suggest that internasal comparisons are likely done outside the MeA.

Despite representing >12.9% of the brain by volume, the cortex contained <0.5% of the total identified →MeA^arom+^ neurons. The only exception to this rule was the piriform cortex, which processes olfactory information and provided clear input to aromatase neurons in the MeA, indicating an indirect route for arom^+^ neurons to integrate input from the main olfactory system with “direct” vomeronasal input from the AOB. There is also a small input from the thalamus, which may represent an alternative pathway of directing cortical information to the arom^+^ MeA neurons. Indeed, integration between the main olfactory and vomeronasal systems is critical for adaptive social behaviors, and these data indicate that inputs from both systems converge on MeA^arom+^ with direct input from the AOB and multisynaptic input from the MOB. Consistent with the survival value of sex, parenting, and territorial defense, the brain regions that provide input to aromatase-expressing neurons are evolutionarily old and conserved (O’Connell and Hofmann, 2011).

The vast majority of inputs to the aromatase-expressing MeA neurons were found in cerebral nuclei, hypothalamus, hippocampal formation, cortical subplate, and associated olfactory areas. The remaining regions collectively comprise <1.3% of total inputs. Thus, the inputome reported in this study suggests an integration of chemosensory input, emotional processing, memory, and sociosexual behavior regulation by MeA aromatase neurons. A combination of inputs from areas that provide information about the metabolic, reproductive, and attentional status of the animal implies a strong effect of the internal status of the animal on the activity of aromatase-expressing MeA cells, which are likely integrated with input from regions involved in voluntary movement and the production of social behaviors. We will briefly discuss these specific inputs by their known functions in the following sections.

Areas involved in detecting chemosensory cues to drive specific sexual discrimination and sociosexual behaviors provide strong input to aromatase-expressing MeA neurons. Inputs from the ventromedial hypothalamus (VMH), posterolateral cortical amygdala (PLCO), posteromedial cortical amygdala, anterior basomedial amygdala, the nucleus of the lateral olfactory tract (NLOT), and AOB were identified (Moncho-Bogani et al., 2005; Maras and Petrulis, 2008; Melis et al., 2009; Yang et al., 2013; Nomoto and Lima, 2015; Hashikawa et al., 2016; Guo et al., 2018). This result is consistent with previous data from MeA, PLCO, AOB, and LOT, which are involved in the interpretation of chemosensory signals (Sosulski et al., 2011; Root et al., 2014; Vaz et al., 2017).

Research into aggression, parenting, and sex consistently implicate the ventromedial hypothalamus (Wang et al., 2015; Kim and Im, 2019) and medial preoptic area (MPOA; Numan, 1988) as control regions for these behaviors. Recent work highlights the important role of galanin-expressing neurons of the MPOA for parental behavior (Wu et al., 2014; Kohl et al., 2018), and a primary source of sensory input for galanin-expressing neurons is the MeA.The MPOA is necessary to produce sex-specific mating and parenting behaviors (Wei et al., 2018). The MPOA also plays a role in driving positive reinforcement for social interactions (Hu et al., 2021). By identifying an arom^+^ MeA neuron-specific interaction between the MPOA and MeA, we can propose a loop between the MeA and MPOA with the potential to mediate positive and negative sex-specific patterns in parental and direct social interaction behaviors. Additional input to arom^+^ MeA neurons from the perifornical area (PeFA), BNST, and amygdalohippocampal area further strengthen the role of the identified MeA arom^+^ circuitry for parental behavior (Lebow and Chen, 2016; Autry et al., 2021). neurons. Combined with previous data showing afferent inputs from the MeA (Pardo-Bellver et al., 2012), these data demonstrate that aromatase-expressing MeA neurons have direct access to a parenting circuit.

Activation of VMH neurons causes immediate territorial aggression (Lin et al., 2011): this is believed to be, at least in part, because of the role of ESR1-expressing neurons in the VMH (Lee et al., 2014). A distinct aggressive behavior, pup-directed aggression, has been linked to the PeFA (Autry et al., 2021), amygdalohippocampal transition area (HATA), and ventromedial hypothalamus. Each of the aggression-linked regions contain neurons that project to aromatase neurons in the MeA, which is consistent with the observation that chemical silencing of aromatase-expressing neurons in the MeA directly reduces aggression in both male and female mice (Unger et al., 2015).

Social interactions can be strongly influenced by fear and anxiety in both animals and people (Sarnoff and Zimbardo, 1961; Beery and Kaufer, 2015). We found that several nodes of the amygdalar fear network send direct input to MeA aromatase neurons. The CeA, basomedial amygdalar nucleus (BMA), BNST, and zona incerta (ZI) had some of the densest projections to the MeA in the brain; each is tightly linked to regulating anxiety and fear responses (Amano et al., 2011; Duvarci et al., 2011; Adhikari et al., 2015; Chou et al., 2018). Initially, this might suggest that aromatase-expressing neurons are directly involved in the fear response to predators. We think this is not the case as the aromatase-expressing neurons receive sensory information from a class of V1R neurons not associated with predator responses (Isogai et al., 2011; Billing et al., 2020). Instead, we predict that these inputs are more likely sculpting behaviors directed toward conspecifics (mates, competitors, and pups) based on the proximity of predators (Apfelbach et al., 2005).

MeA aromatase neurons receive direct input from the hippocampus, known for its role in memory formation and consolidation (Scoville and Milner, 1957). Hippocampal inputs to the MeA were not evenly distributed throughout the hippocampus, but rather were concentrated in the posterior ventral hippocampus including the HATA, CA1, CA2, and CA3. These are also the hippocampal regions anatomically closest to the MeA. Previous data indicate that region CA2 is particularly involved in memory and spatial processing but is also highly receptive to social state and plays a role in the deduction of social novelty (Dudek et al., 2016). Similarly, the MeA has been linked to social memory (Ferguson et al., 2001), and MeA aromatase neurons have been directly implicated in social discrimination (Yao et al., 2017). We identified direct input from the ventral tegmental area (VTA), and together these sources of input may help to elevate and reinforce responses to emotionally relevant stimuli (Zikopoulos and Barbas, 2012). We also identified inputs from areas involved in the regulation of voluntary movement, suggesting a potential for gating aromatase neuron-dependent behaviors.

A broad set of identified inputs to MeA aromatase neurons is associated with metabolic regulation, including the tuberal nucleus (TN), PMv, lateral hypothalamus, and VMH. However, a hallmark of social behavior is that the pattern of social behaviors observed in young animals is different from those observed in adult animals. The transition from juvenile to adult behaviors depends on metabolic constraints, including body weight and fat content (Castellano and Tena-Sempere, 2016). The observed input from brain regions that regulate the metabolic state (Nisbett, 1972; Berthoud and Munzberg, 2011; Donato et al., 2011; Luo et al., 2018) further supports the role of the MeA as a node responsible for coordinating social behaviors in accord with internal state, reproductive readinesss, and environmental contexts (Padilla et al., 2016). This suggests that information about sexual receptivity and readiness are integrated with other relevant cues about conspecifics in the aromatase-expressing cells in the MeA.

In accord with the role of MeA, and aromatase neurons, in sexually dimorphic social behaviors, the MeA displays structural, molecular, and functional sex differences (Nishizuka and Arai, 1981; De Vries et al., 1984; Cooke et al., 1999; Morris et al., 2008a; Bergan et al., 2014; Billing et al., 2020). In fact, the domestication process of creating isogenic mouse strains may have accentuated sex differences in circuit function (Chalfin et al., 2014; Bansal et al., 2021). We found that the broad pattern of regional inputs to aromatase-expressing MeA neurons was conserved across males and females; however, a number of regions displayed a quantitative difference in the percentage of inputs observed in males compared with females. Of the 10 regions identified as having a quantitative sex difference, 9 had more labeled neurons in males. One possible explanation for this male bias is that the percentage of connections made by aromatase-expressing neurons inside the MeA is higher in females compared with males. This hypothesis is partially supported by our data, which indicate more neurons labeled in the female MeA versus the male MeA; however, this trend did not reach significance. To account for variability in the levels of starter neuron infection, our analyses here rely on normalizing cell counts in each region by the overall number of identified neurons, and, therefore, we cannot distinguish between a reduction in the number of local connections and an enhancement of long-range connections.

The regions displaying sex differences provide new insights into the function of aromatase-expressing MeA circuits. The ventral premammillary nucleus was the only region identified with more neurons labeled in females, and this region has recently been identified as a critical mediator of maternal aggression (Motta et al., 2013). This is consistent with the role of aromatase-expressing MeA neurons in aggression in female mice (Unger et al., 2015). Regions with more neurons in males are linked to fear and anxiety (Yang and Wang, 2017), social and contextual memory (Okuyama et al., 2016), mate identification and olfaction (Cádiz-Moretti et al., 2016; Wang et al., 2020a), regulation of neuropeptide secretion (Brown, 2016), and male-specific sexual behavior (Schmidt et al., 2000). Still, like the social behaviors mediated by MeA arom^+^ neurons in mice, the circuits that mediate these behaviors are sculpted differently in males and females, and these differences in circuit connectivity likely support sex differences in social behavior.

We comprehensively identified the inputome of aromatase-expressing MeA neurons, finding the sources of direct synaptic input to aromatase-expressing MeA neurons through rabies-based circuit mapping in the intact brains from transgenic animals expressing cre-recombinase under the control of the aromatase promoter. Presynaptic inputs were identified through a semiautomated algorithm, which provided an unbiased view of the circuit. Data from each individual are set in a standard reference frame that allows direct comparisons among brains, as well as to future datasets using a similar approach.

Our study confirms that known sources of synaptic input to MeA neurons (e.g., BNST and AOB) synapse specifically on arom^+^ neurons in the MeA. However, we also identified a broad set of subcortical inputs that have not been previously reported, indicating that aromatase neurons in the MeA represent a critical node for integrating an array of internal states for the generation of social behaviors. The broad input to aromatase neurons reveals a framework that allows the aromatase-expressing MeA neurons to occupy a critical position in the social behavior network, with a role in nearly all known social behaviors ranging from parenting and reproduction to aggression and learning. While the overall pattern of inputs was similar in males and females, specific regions displayed sex differences in connectivity with aromatase-expressing MeA neurons further solidifying the role of these neurons as a control center for sex-specific social behaviors that are critical for survival.

## Synthesis

Reviewing Editor: Christopher Colwell, UCLA School of Medicine Decisions are customarily a result of the Reviewing Editor and the peer reviewers coming together and discussing their recommendations until a consensus is reached. When revisions are invited, a fact-based synthesis statement explaining their decision and outlining what is needed to prepare a revision will be listed below. The following reviewer(s) agreed to reveal their identity: Margaret McCarthy.

Dear Drs. Dwyer, Bergan, and Kelly,

Your study “Brain-wide synaptic inputs to aromatase-expressing neurons in the medial amygdala suggest complex circuitry for modulating social behavior” has been review by two experts. Both were impressed by your work with one calling it “an exhaustive and beautifully done descriptive analysis”.

Both brought up constructive concerns that should be addressed. These concerns fall into requests for clarification on the methodology including statistics as well as some issue to be handled in the discussion.

Methodological issues

The methods state that both males and females are used for analysis, but the authors do not address whether the results apply equally to males and females. Considering that the MEA is one of the few regions of the brain that has both structural and molecular sex differences, it would be surprising to me if there were no sex differences (although if there are none, the authors should report this).

There was insufficient detail on how the authors determined whether a specific region did or did not send projections to MEA aromatase neurons. The authors state that an automated pipeline is used (that has been published), but no details are provided. The authors need to provide more details on how inputs were determined “automatically”. It seems that such a process would be dependent on many parameters and these should be reported. As such, it is impossible to determine from this manuscript what is being compared in statistical analyses presented through-out the manuscript.

Similarly, on pg 6 the authors state that “significant inputs” come from the VMH. Do they mean statistically significant or more substantial than other areas?

The authors should state in the results section that cre-dependent viruses are used so that readers do not need to check the methods section to confirm this.

The authors state that all of the images and data will be made available in a repository but provide no details of where. It should be possible to get an accession number and make the data available upon publication.

Throughout the Results, the authors provide t test statistics, but the comparisons being made are not clear. For example, in Figure 4 (and subsequent similar figures) what is the comparison for any given region that the significance indicates (e.g. CNU vs ???, p =***).

Each figure does an excellent job of portraying the percentages of cells and their relative abundance within each major brain region. However, I found myself continually cross referencing these figures as there was no “master” figure to illustrate which regions belonged to which categories. Perhaps the authors should consider adding a hierarchical diagram that visually depicts the data presented in the 1st paragraph under “Population data: finer-scale brain regions”. This way, the reader would have a visual complement that shows the five major regions identified, with subregions listed underneath. Such a figure might fit within Figure 3, where the authors already provide a depiction of the region-associated inputs.

Issues for discussion

The authors state the MEA is portrayed as a third node in the vomeronasal pathway. Thus, it seems a little surprising that less than 2% of inputs into MEA aromatase neurons come from the AOB. Is this because there are so many inputs from other brain regions or because there are few inputs onto aromatase neurons. Is there anyway to know how many neurons there are in AOB? Maybe the 2% of projections into MEA aromatase neurons comprises 20% of AOB neurons?

The discussion of the cerebral nuclei, which comprised the vast majority of inputs to the MEA could be strengthened given that this seemed to be a major result in the paper. As the authors describe, the field often considers MEA to be more a sensory processing node yet the results in this manuscript suggest that this may be an inaccurate view. The authors indicate that these projections could mediate transfer of information about metabolic, reproductive or attentional status. However, I think authors could do more to address the implication that MEA aromatase neurons play a more integrative role.

While the authors refer to the function of the aromatase-expressing MeA cells in the context of sex-specific behaviors, they make no mention of possible sex differences in their analysis. The study appears to include a significant number of male and female mice, but it is not clear whether any analysis of sex as a variable was conducted or whether it is appropriately powered to detect any differences that may exist. Given that there is a sex difference in the aromatase+ population within the MeA, such an analysis (or at least a discussion pertaining to sex differences) seems essential.

Somewhat related to the above, on Pg 6 line 256 - the authors refer to the PVN as a sexually dimorphic nucleus. The reference provided is a review article and notes that the PVN makes oxytocin which has been implicated in parenting. Generally the PVN is not a sight of many sex differences and has the role of oxytocin in maternal behavior is more strongly linked to the SON. Moreover, mice lacking oxytocin receptors are still maternal so this is a tenuous connection at best. The subsequent discussion of the inputs from various regions, including MPOA and VMN, seems much more well-grounded.

## Author Response

Dear reviewers and editor Christopher Colwell,

We appreciate the recognition that our manuscript “Brain-wide synaptic inputs to aromatase-expressing neurons in the medial amygdala suggest complex circuitry for modulating social behavior” represents “an exhaustive and beautifully done descriptive analysis”. The reviewers’ comments were accurate and constructive, and we believe we have now addressed each point raised during the previous round of review. We thank the editor and reviewers for the effort all put towards improving this manuscript and believe the changes we’ve made to address these comments improved our manuscript. We have added new data and new analyses as requested. Changes to text are highlighted in yellow and additionally indicated with explanation below. We now include tables with descriptive statistics and the attached figures are at 400 DPI. A point-by-point response is included below.

[1] The methods state that both males and females are used for analysis, but the authors do not address whether the results apply equally to males and females. Considering that the MEA is one of the few regions of the brain that has both structural and molecular sex differences, it would be surprising to me if there were no sex differences (although if there are none, the authors should report this).

[Response 1]. We originally were under powered to confidently make claims about sex differences. We have collected new data, performed new analyses, and added Figure 9 and Figure S9 to address this critical point. We have added the following discussion to the results section:

Line 294:

Sex Differences in distribution of observed input cells

Because sex differences in the MeA, and specifically in MeAarom+ neurons, are well established (Cooke, et al., 2005; Yao et al., 2017; Billing et al., 2019) we investigated sex differences in synaptic input to MeAarom+ neurons. A regression analysis of the cell counts for all male regions against cell counts for all female regions indicated that the major regions providing synaptic input to MeAarom+ neurons are largely conserved across sexes (Figure 9; R2 = 0.60252; F = 39.4127; p < 0.00001; linear regression). No significant difference was observed for sex when considering all regions (ANOVA: F = 0.04 p = 0.83); however, there was a significant interaction between region and sex (ANOVA: F = 1.81, p = 0.01) indicating that a subset of brain regions displayed sex differences in projections to MeAarom+ neurons. Post-hoc analyses revealed nine regions that provided more input in males and one region that provided more input in females (Figure 9). MeAarom+ neurons received more input from the AOB, basolateral amygdala (both anterior and ventral), CA1 region of the hippocampus, piriform areas, diagonal band nucleus, and lateral preoptic area in males and more input from the ventral premammillary nucleus in females (p<0.05: permutation test; Table 4). Additionally, the fraction labeled neurons in the MeA, local to the injection site), was higher in females (29.2 {plus minus} 10.7) than males though this trend did not reach significance (13.0 {plus minus} 3.5; p=0.08: permutation test; Figure S9).

And the following text to the discussion:

Line 447:

In accord with the role of MeA, and aromatase neurons, in sexually dimorphic social behaviors, the MeA displays structural, molecular, and functional sex differences (Nishizuka and Arai, 1981; De Vries et al., 1984; Cooke et al., 1999; Morris et al., 2008; Bergan et al, 2014; Billing et al., 2019). In fact, the domestication process of creating isogenic mouse strains may have accentuated sex-differences in circuit function (Chalfin, et al., 2014; Bansal, et al., 2021). We found that the broad pattern of regional inputs to aromatase-expressing MeA neurons was conserved across males and females; however, a number of regions displayed a quantitative difference in the percentage of inputs observed in males as compared to females. Of the ten regions identified as having a quantitative sex-difference, nine had more labeled neurons in males. One possible explanation for this male bias is that the percentage of connections made by aromatase-expressing neurons inside the MeA is higher in females as compared to males. This hypothesis is partially supported by our data, which indicates more neurons labeled in the female MeA versus the male MeA; however, this trend did not reach significance. To account for variability in the levels of starter neurons infection, our analyses here rely on normalizing cell counts in each region by the overall number of identified neurons, and therefore, we cannot distinguish between a reduction in the number of local connections versus an enhancement of long-range connections.

The regions displaying sex differences provide new insights into the function of MeA arom+ circuits. The ventral premammillary nucleus was the only region identified with more neurons labeled in females and this region has recently been identified as a critical mediator of maternal aggression (Motta, et al., 2013). This is consistent with the role of MeA arom+ neurons in aggression in female mice (Unger et al., 2015). Regions with more neurons in males are linked to fear and anxiety (Yang, Y, et al., 2017), social and contextual memory (Okuyama, et al., 2016), mate identification and olfaction (Cádiz-Morretti, B., 2016; Wang, L, 2020), regulation of neuropeptide secretion (Brown, 2016), and male-specific sexual behavior (Schmidt, et al., 2000). Still, like the social behaviors mediated by MeA arom+ neurons in mice, the circuits that mediate these behaviors are sculpted differently in males and females, and these differences in circuit connectivity likely support sex differences in social behavior.

[2] There was insufficient detail on how the authors determined whether a specific region did or did not send projections to MEA aromatase neurons. The authors state that an automated pipeline is used (that has been published), but no details are provided. The authors need to provide more details on how inputs were determined “automatically”. It seems that such a process would be dependent on many parameters and these should be reported. As such, it is impossible to determine from this manuscript what is being compared in statistical analyses presented through-out the manuscript.

[Response 2] We agree and the following text was added to the methods section.

Line 131: Cell-counting: Rabies-labeled cells were identified using a human-trained computer vision algorithm (Ilastik, Heidelberg University). Briefly, a training subset of images were annotated as ’not tissue’, ’unlabeled tissue’, and ’rabies labeled neuron’ by a trained user to prepare a machine learning kernel to automatically identify rabies labeled neurons. The resulting kernel was refined over several iterations until it accurately identified rabies labeled cells. Ilastik then automatically classified each pixel in from each image based on its probability of being a member of each category. The resulting probability map for ’rabies labeled neuron’ were segmented with ’regionprops’ in MATLAB and the centroids of each identified neuron were identified. Automatically identified cells (ilastik, Heidelberg University) were reviewed and confirmed by a human observer for quality control.

[3] Similarly, on pg 6 the authors state that “significant inputs” come from the VMH. Do they mean statistically significant or more substantial than other areas?

[Response 3] This is a fair point. The results section now reads:

Line 262: ’The two hypothalamic regions providing the largest number of ->MeAarom+ neurons to the inputome are the lateral hypothalamic area (2.1 {plus minus} 0.17; t(17)=3.2,p=0.004) and ventromedial hypothalamic nucleus (1.2 {plus minus} 0.16; t(17)=1.9, p=0.04; Figure 6A; Table 3).

[4] The authors should state in the results section that cre-dependent viruses are used so that readers do not need to check the methods section to confirm this.

[Response 4] The following text has been added to clarify this point:

Line 193: The MeApd of adult male and female aromatase-cre mice was sterotaxically injected with double-floxed inverse orientation adeno-associated viruses that selectively express the EnvA receptor (TVA) and rabies glycoprotein (RG) in aromatase-expressing neurons following cre-based recombination (Figure 2A; see methods).

[5] The authors state that all of the images and data will be made available in a repository but provide no details of where. It should be possible to get an accession number and make the data available upon publication.

[Response 5] The online data repository eBrains (https://ebrains.eu/) has agreed to host our datasets and we are working with them to curate and make these data available. The following text has been added to the methods section.

Line 495: These data have been uploaded to ebrains online repository (Ref. 17110259) for future access and to allow reproducibility.

[6] Throughout the Results, the authors provide t test statistics, but the comparisons being made are not clear. For example, in Figure 4 (and subsequent similar figures) what is the comparison for any given region that the significance indicates (e.g. CNU vs ???, p =***).

[Response 6] This has now been clarified in the methods section and a table of all test results including predictive stats is now included:

Line 165: Regions identified as providing input to MeA arom+ neurons were determined using an single sample t-test on the observed cell counts in each region with Bonferroni correction for multiple comparisons when appropriate. A second comparison was made using a single sample t-test to determine if the input from a given region exceeded the expected input based on chance using the region volume and an even distribution of cells as the chance rates (see Table 3).

[7] Each figure does an excellent job of portraying the percentages of cells and their relative abundance within each major brain region. However, I found myself continually cross referencing these figures as there was no “master” figure to illustrate which regions belonged to which categories. Perhaps the authors should consider adding a hierarchical diagram that visually depicts the data presented in the 1st paragraph under “Population data: finer-scale brain regions”. This way, the reader would have a visual complement that shows the five major regions identified, with subregions listed underneath. Such a figure might fit within Figure 3, where the authors already provide a depiction of the region-associated inputs.

[Response 7] Yes, this is a challenging issue with this dataset given that it explores the entire brain. The manuscript now includes an ’icon’ of the coarse region pie chart with the relevant coarse region highlighted for each figure that explores fine regions. Text such as the following were added to each figure legend for clarity.

Figure 5 legend: Left, a pie chart indicates the fraction of all labeled neurons in the hypothalmus and the brain atlas depicts the position of hypothalamic regions.

[8] The authors state the MEA is portrayed as a third node in the vomeronasal pathway. Thus, it seems a little surprising that less than 2% of inputs into MEA aromatase neurons come from the AOB. Is this because there are so many inputs from other brain regions or because there are few inputs onto aromatase neurons. Is there anyway to know how many neurons there are in AOB? Maybe the 2% of projections into MEA aromatase neurons comprises 20% of AOB neurons?

[Response 8] Our experiments are centered around aromatase-expressing neurons in the MeA. If we label more MeA ’starter neurons’ we will observe more neurons labeled in the AOB and all other regions (see Figure 2d). For this reason, we aren’t able to accurately state what the fraction of AOB neurons that project to the MeAarom+ neurons is. Instead, we can say that an MeAarom+ neuron receives a certain proportion of input from any set of regions. We include supplementary figures that provide cell counts scaled by region size, and this partially addresses the reviewer’s question. That is, it provides the frequency of identified projection neurons independent of region size.

The following text was added to help clarify this point:

Line 327: Two regions that are classically associated with the MeA are the AOB and BNST (Scalia, et al., 1975). The MeA is often illustrated as the third node in the vomeronasal pathway: vomeronasal sensory neurons project to the AOB that in turn projects to the MeA (Scalia et al., 1975). Similarly, the BNST and MeA are together considered the extended amygdala, a collective region that processes sensory and social information (Marino, et al., 2016). Using classical retrograde tracing it has been previously shown that most AOB neurons project to the MeA (De Olmos, et al., 1978; Bergan, et al., 2014, with aromatase neurons receiving input overwhelmingly from the anterior subdivision of the AOB (Billing, et al., 2019). Our tracing results demonstrate a clear input to aromatase-expressing MeA neurons from both the AOB and the BNST. However, the collective inputs from the AOB and BNST account for less than 2 percent of the overall ->MeAarom+ neurons. This result should not be interpreted as evidence that few AOB neurons project to the MeA, but rather, it highlights the larger set of projections coming from areas other than the AOB and BNST. Aromatase-expressing neurons clearly have a wide-ranging role as integrators of information from inputs throughout the brain, illustrating the danger in thinking of the social behavior network as a simple feed-forward network. Moreover, our results indicate that many known postsynaptic targets of the MeA are also presynaptic inputs to aromatase expressing MeA neurons, suggesting that the MeA forms feedback loops with many regions in the social behavior network, even at the level of the population of aromatase-expressing neurons.

[9] The discussion of the cerebral nuclei, which comprised the vast majority of inputs to the MEA could be strengthened given that this seemed to be a major result in the paper. As the authors describe, the field often considers MEA to be more a sensory processing node yet the results in this manuscript suggest that this may be an inaccurate view. The authors indicate that these projections could mediate transfer of information about metabolic, reproductive or attentional status. However, I think authors could do more to address the implication that MEA aromatase neurons play a more integrative role.

[Response 9] We completely agree with this comment and have made many changes in the text to, hopefully, highlight this point. In particular, the following text was added:

Line 434: A broad set of identified inputs to MeA aromatase neurons are associated with metabolic regulation, including the tuberal nucleus (TN), ventral premammillary nucleus (VPM), lateral hypothalamus (LH), and ventromedial hypothalamus (VMH). However, a hallmark of social behavior is that the pattern of social behaviors observed in young animals is different from those observed in adult animals. The transition from juvenile to adult behaviors depends on metabolic constraints including body weight and fat content (Castellano, et al., 2016). The observed input from brain regions that regulate metabolic state (Luo, et al., 2018; Donato, et al., 2010; Nisbett 1972; Berthoud and Munzberg 2011) further supports the role of the MeA as a node responsible for coordinating social behaviors in accord with internal state, reproductive readinesss, and environmental contexts (Padilla et al., 2016). This suggests that information about sexual receptivity and readiness are integrated with other relevant cues about conspecifics in the aromatase-expressing cells in the MeA.

[10] While the authors refer to the function of the aromatase-expressing MeA cells in the context of sex-specific behaviors, they make no mention of possible sex differences in their analysis. The study appears to include a significant number of male and female mice, but it is not clear whether any analysis of sex as a variable was conducted or whether it is appropriately powered to detect any differences that may exist. Given that there is a sex difference in the aromatase+ population within the MeA, such an analysis (or at least a discussion pertaining to sex differences) seems essential.

[Response 10] We agree, see response to point 1 above.

[11] Somewhat related to the above, on Pg 6 line 256 - the authors refer to the PVN as a sexually dimorphic nucleus. The reference provided is a review article and notes that the PVN makes oxytocin which has been implicated in parenting. Generally the PVN is not a sight of many sex differences and has the role of oxytocin in maternal behavior is more strongly linked to the SON. Moreover, mice lacking oxytocin receptors are still maternal so this is a tenuous connection at best. The subsequent discussion of the inputs from various regions, including MPOA and VMN, seems much more well-grounded.

[Response 11] We agree and have cut this discussion as it was not well supported and not central to the goals of this manuscrip
