# Supplementary figures and images for: Brain-Wide Synaptic Inputs to Aromatase-Expressing Neurons in the Medial Amygdala Suggest Complex Circuitry for Modulating Social Behavior
Source: eNeuro. 2022 Mar 11;9(2):ENEURO.0329-21.2021. doi: 10.1523/ENEURO.0329-21.2021 (PMC8925724; doi:10.1523/ENEURO.0329-21.2021)

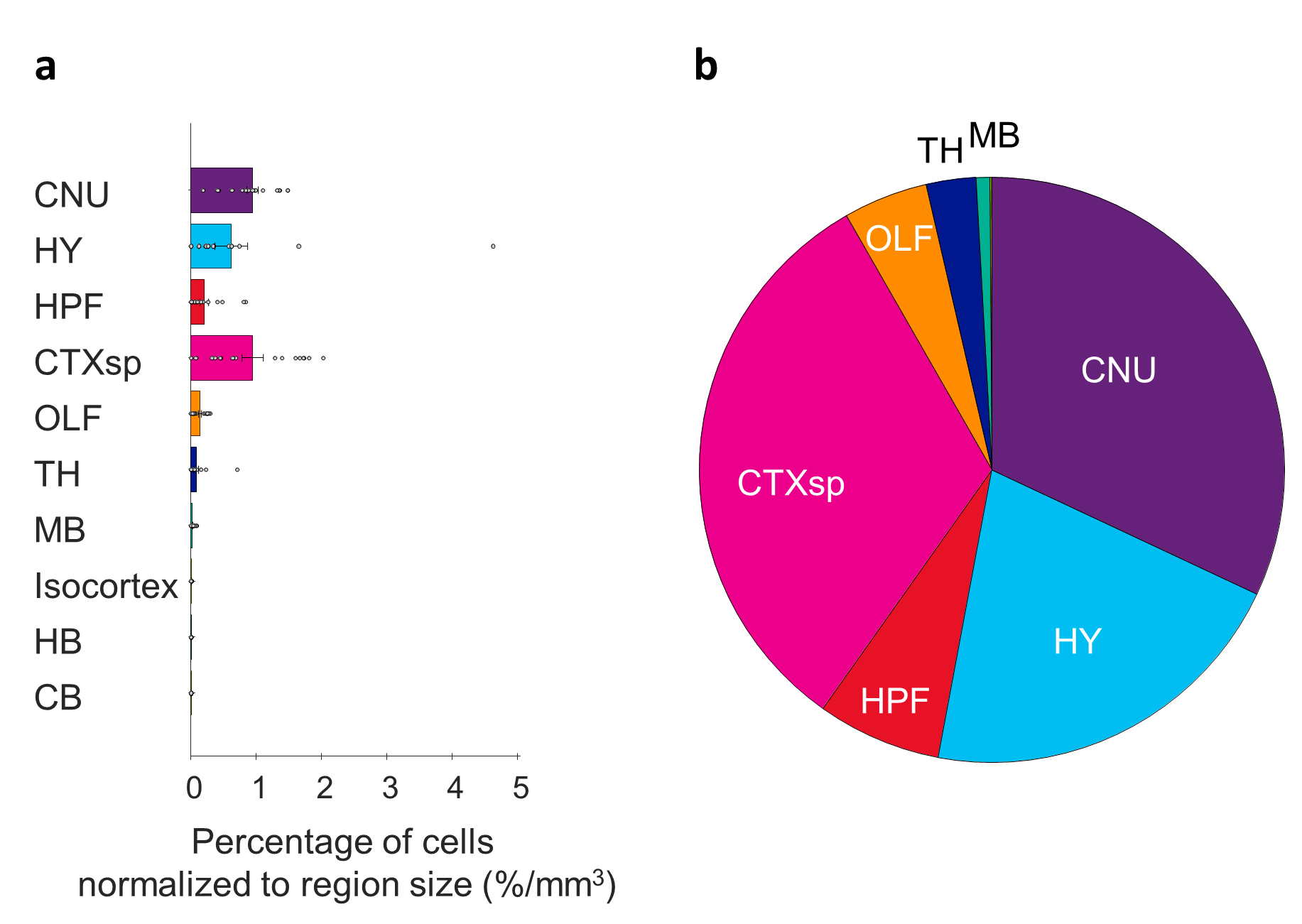

Supplement: Figure 4-1 — Input cell density in regions brain wide. Percentage of input cells in each coarse region identified in the Allen Brain Atlas normalized to the volume of that region to determine cell densities in each region are presented as graph (a) and pie chart (b). The densest concentration of input cells is found in the cortical subplate (CTXsp). Download Figure 4-1, TIF file. [file enu-eN-NWR-0329-21-s01.tif]

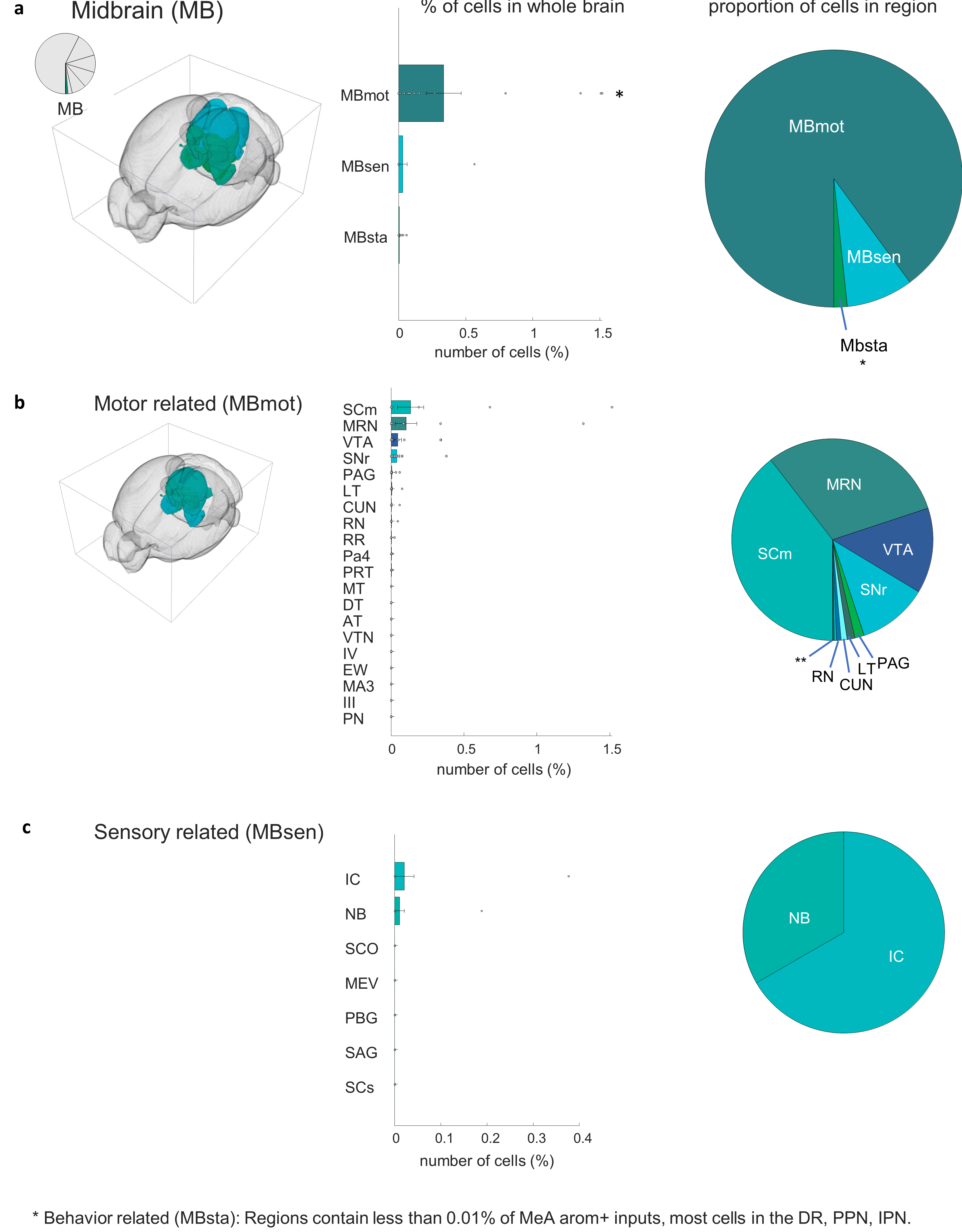

Supplement: Figure 4-2 — MeA arom+ inputome originating in the midbrain (MB). a, Bar graph depicts the percentages of input cells found in major divisions of the midbrain relative to the whole-brain inputome; and pie chart shows the relative proportion of those cells within those regions. Regions shown: motor-related midbrain regions (MBmot), sensory-related midbrain regions (MBsec), and behavior-related midbrain regions (MBsta). b, Inputs from motor-related midbrain regions: bar graph depicts the percentages of input cells found in major divisions of these regions relative to the whole-brain inputome; and pie chart shows the relative proportion of input cells within those regions. Regions shown: midbrain reticular nucleus (MRN), superior colliculus—motor (SCm), VTA, substantia nigra (SNr), periaqueductal gray (PAG), lateral terminal nucleus of accessory optic tract (LT), cuneiform nucleus (CUN), red nucleus (RN), retrorubal area of midbrain reticular nucleus (RR), paratrochlear nucleus (Pa4), pretectal region (PRT), medial terminal nucleus of accessory optic tract (MT), dorsal terminal nucleus of accessory optic tract (DT), anterior tegmental nucleus (AT), ventral tegmental nucleus (VTN), trochlear nucleus (IV), Edinger-Westphal nucleus (EW), medial accessory oculomotor nucleus (MA3), oculomotor nucleus (III), and paranigral nucleus (PN). c, Inputs from sensory-related midbrain regions: bar graph depicts the percentages of input cells found in major divisions of these regions relative to the whole-brain inputome; and pie chart shows the relative proportion of input cells within those regions. Regions shown: inferior colliculus (IC), brachium of inferior colliculus (NB), subcommissural organ (SCO), midbrain trigeminal nucleus (MEV), parabigeminal nucleus (PBG), nucleus sagulum (SAG), and superior colliculus (sensory; SCs). **Less than 0.01% of MeA arom+ inputs originate in behavior-related midbrain area; most of those cells are found in the dorsal raphe nucleus (DR), pedunculopontine nucleu [file enu-eN-NWR-0329-21-s02.tif]

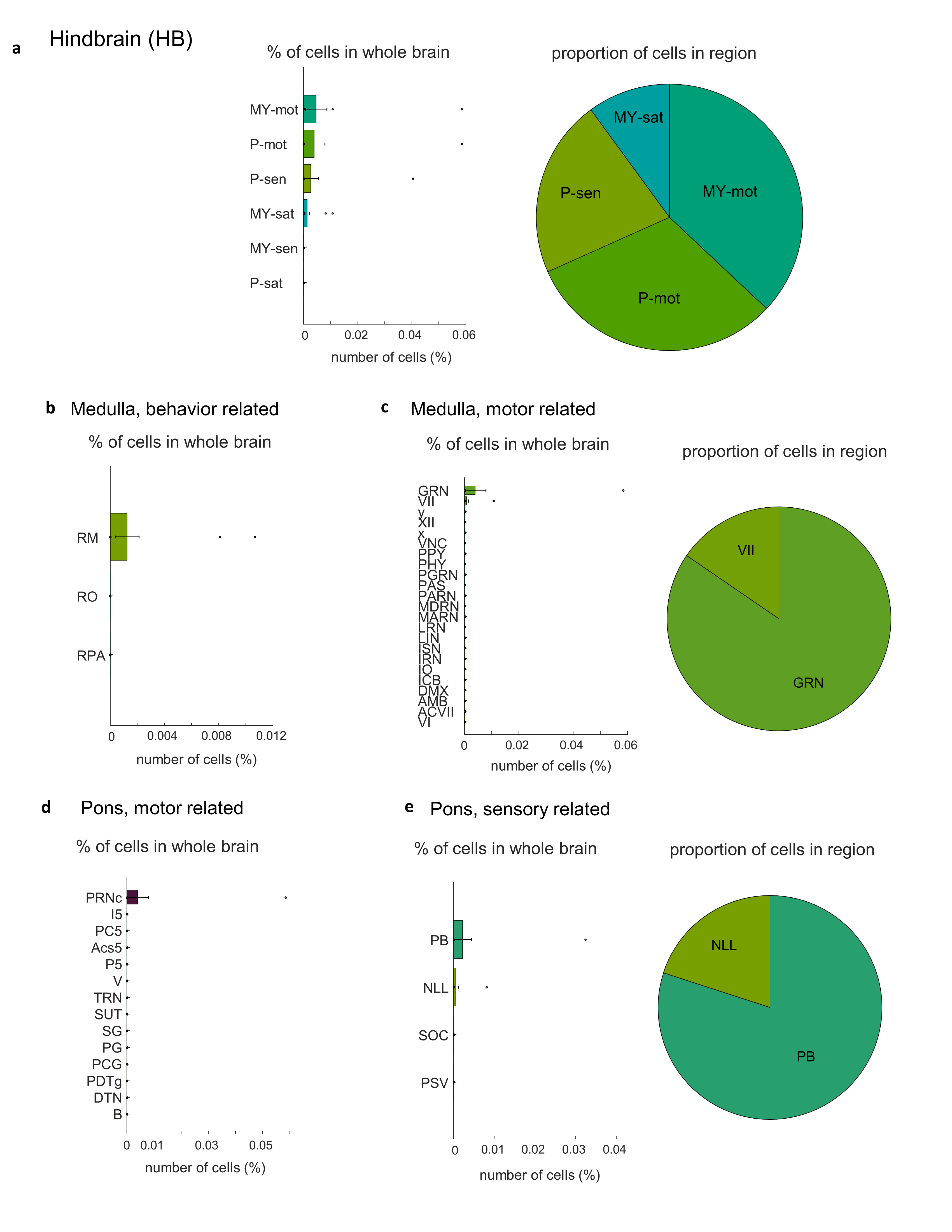

Supplement: Figure 4-3 — MeA arom+ inputome originating in the hindbrain (HB). a, Whole hindbrain: bar graph depicts the percentages of input cells found in major divisions of the hindbrain relative to the whole-brain inputome; and pie chart shows the relative proportion of input cells within those regions. Regions shown: motor-related medulla (MY-mot), sensory-related medulla (MY-sen), behavioral state medulla (MY-sat), motor-related pons (P-mot), sensory-related pons (P-sen), and behavioral state pons (P-sat). b, Only one of the behavior-state portions of the medulla, the nucleus raphe magnus (RM) contains inputs to arom+ cells in the MeA. c, Bar graph depicts the percentages of input cells found in major divisions of the motor-related medulla relative to the whole-brain inputome; and pie chart shows the relative proportion of input cells within those regions. Two regions of the motor-related medulla provide inputs: the gigantocellular reticular nucleus (GRN) and the facial motor nucleus (VII). d, The caudal part of the pontine reticular nucleus (PRNc) is the only part of the motor-related pons to provide inputs to arom+ MeA cells. e, In the sensory-related pons, inputs originate from the parabrachial nucleus (PB) and the nucleus of the lateral lemniscus (NLL). Download Figure 4-3, TIF file. [file enu-eN-NWR-0329-21-s03.tif]

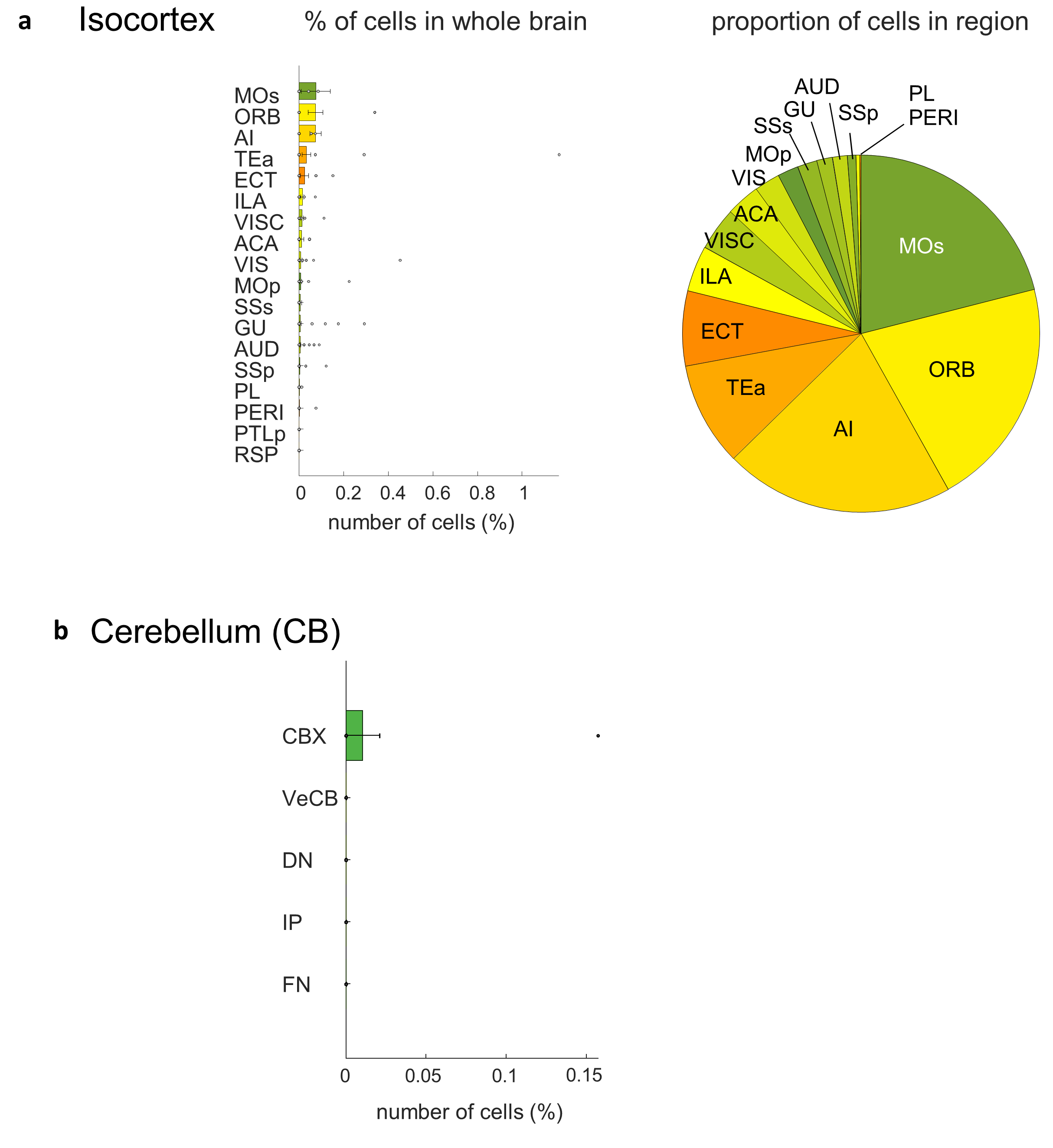

Supplement: Figure 4-4 — MeA arom+ inputome originating in the isocortex and the cerebellum (CB). a, Bar graph depicts the percentages of input cells found in major divisions of the isocortex relative to the whole-brain inputome; and pie chart shows the relative proportion of input cells within those regions. Regions shown: Secondary motor area (MOs), agranular insular area (AI), orbital area (ORB), temporal association area (Tea), ectorhinal area (ECT), visceral area (VISC), anterior cingulate area (ACA), infralimbic area (ILA), primary motor area (MOp), supplementary somatosensory area (SSs), gustatory areas (GU), auditory areas (AUD), visual areas (VIS), primary somatosensory area (SSp), prelimbic area (PL), perirhinal area (PERI), posterior parietal association areas (PTLps), and retrosplenial area (RSP). b, Inputs from the cerebellum are only found in the cerebellar cortex (CBX). Download Figure 4-4, TIF file. [file enu-eN-NWR-0329-21-s04.tif]

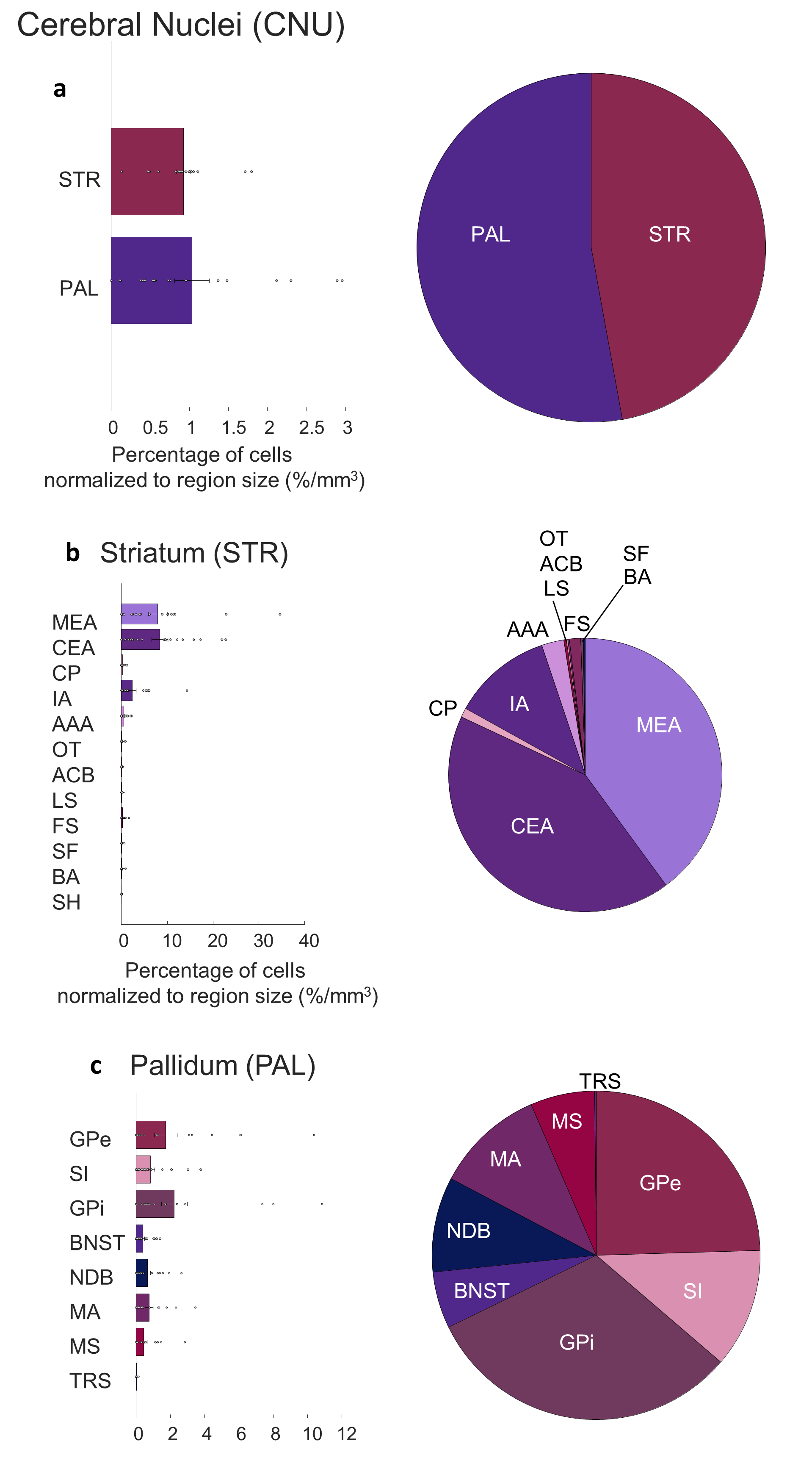

Supplement: Figure 5-1 — Input cell density in the cerebral nuclei (CNU). a, Input cell densities of constituent regions of the cerebral nuclei. b, Input cell densities of constituent regions in the striatum. c, Input cell densities of constituent regions in the pallidum. Results are presented as both bar chart and pie charts. Download Figure 5-1, TIF file. [file enu-eN-NWR-0329-21-s05.tif]

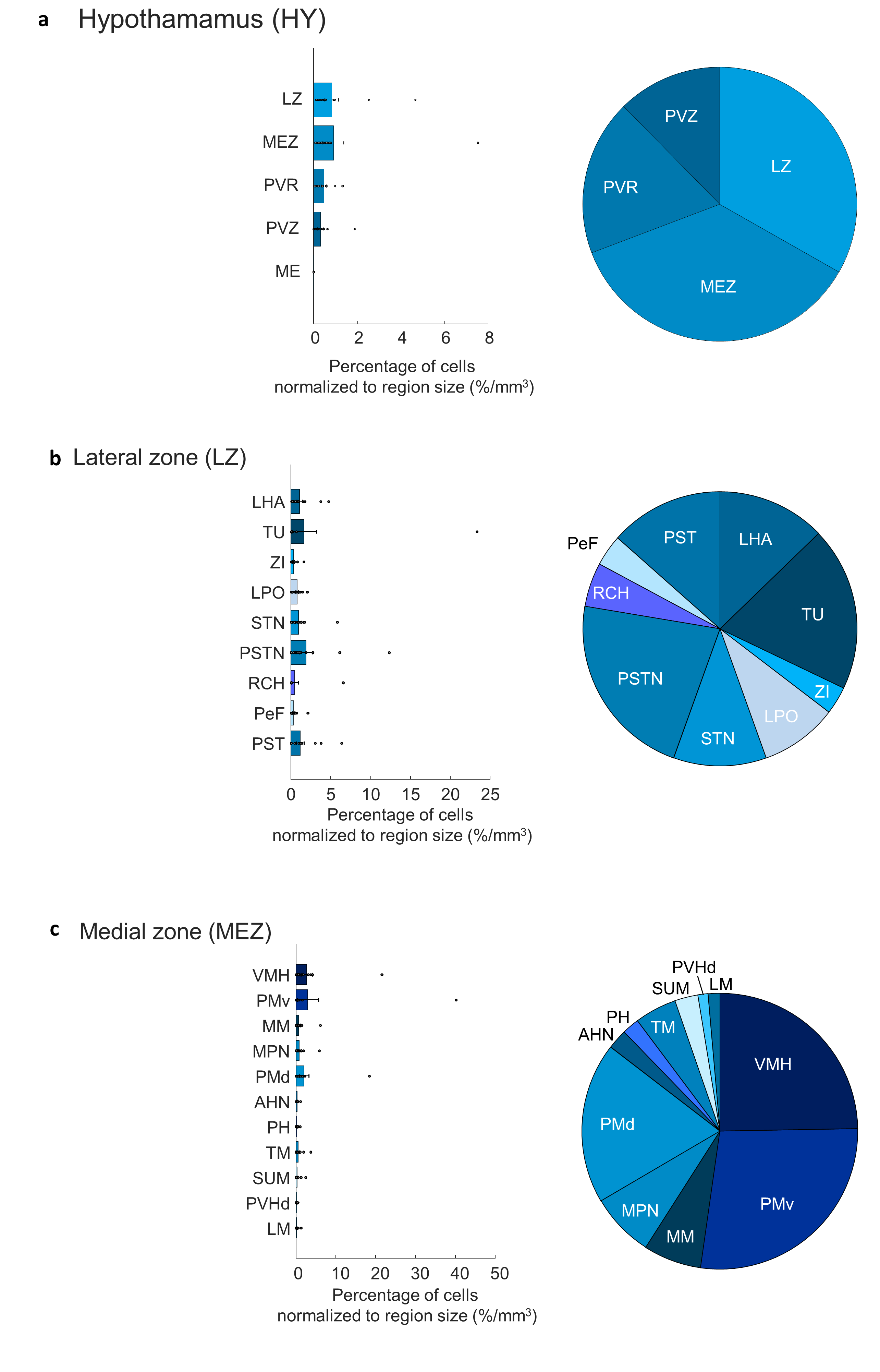

Supplement: Figure 6-1 — Input cell density in the hypothalamus (HY). a, Input cell densities of constituent regions in the hypothalamus. b, Input cell densities of constituent regions in the lateral zone of the hypothalamus. c, Input cell densities of constituent regions in the medial zone of the hypothalamus. Results are presented as both bar chart and pie charts. Download Figure 6-1, TIF file. [file enu-eN-NWR-0329-21-s06.tif]

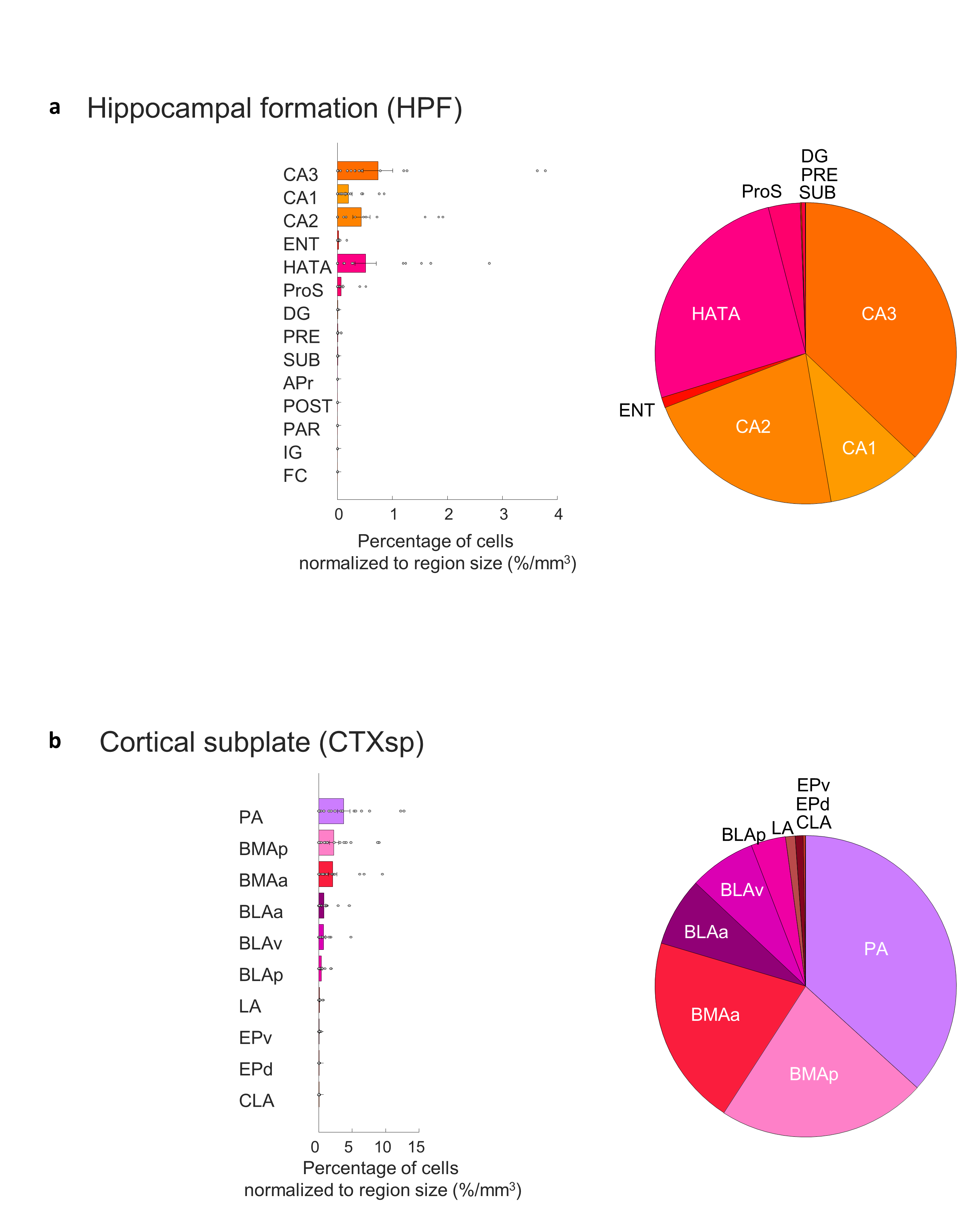

Supplement: Figure 7-1 — Input cell density in the hippocampal formation (HPF) and the cortical subplate (CTXsp). a, Input cell densities of constituent regions in the hippocampal formation. b, Input cell densities of constituent regions in the cortical subplate. Results are presented as both bar chart and pie charts. Download Figure 7-1, TIF file. [file enu-eN-NWR-0329-21-s07.tif]

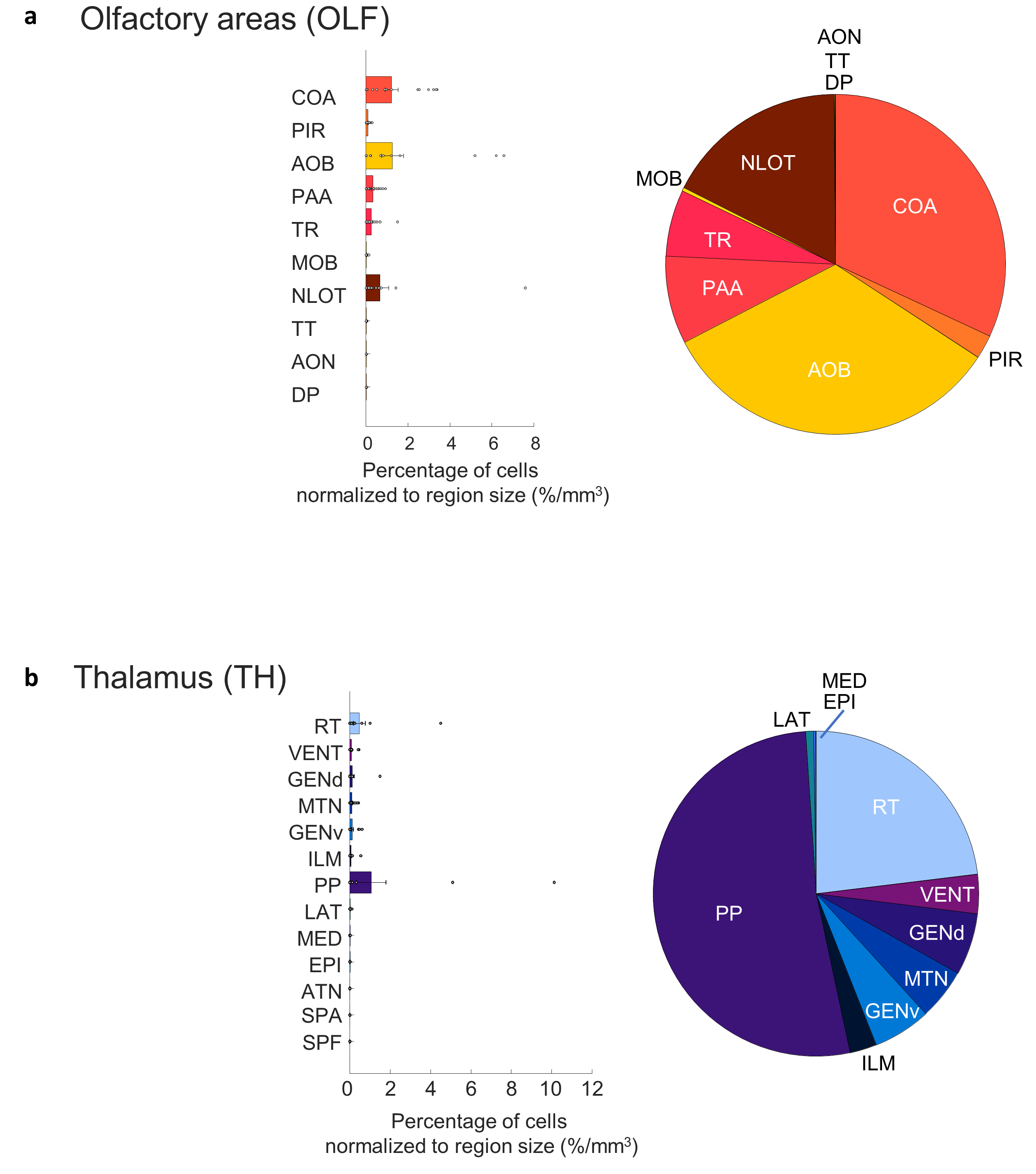

Supplement: Figure 8-1 — Input cell density in the olfactory areas (OLF) and the thalamus (TH). a, Input cell densities of constituent regions in the olfactory areas. b, Input cell densities of constituent regions in the thalamus. Results are presented as both bar chart and pie charts. Download Figure 8-1, TIF file. [file enu-eN-NWR-0329-21-s08.tif]

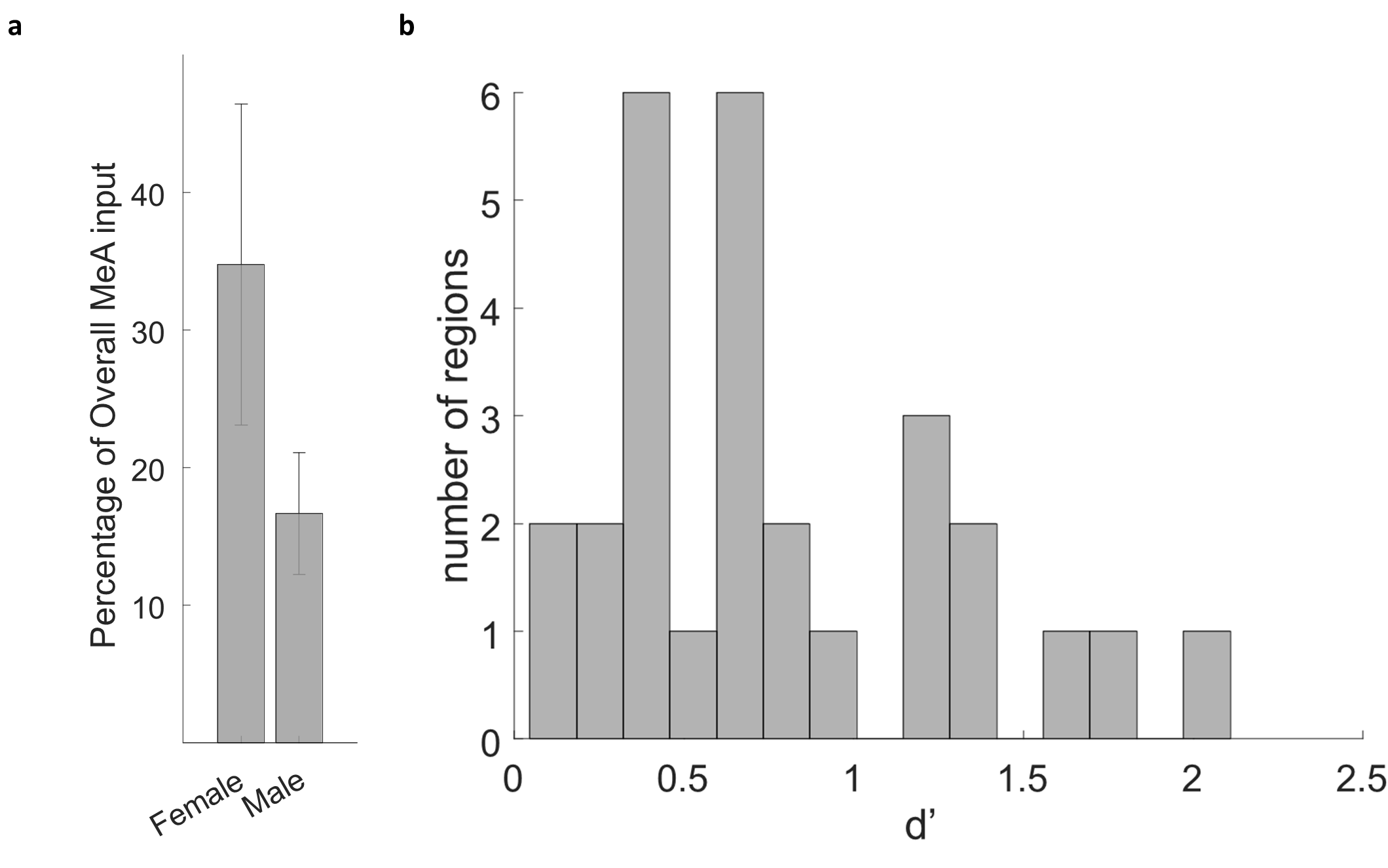

Supplement: Figure 9-1 — Comparison of sex differences in observed inputs to MeAArom+ Cells. a, Graph comparing the fraction of labeled neurons in the MeA, local to the injection site, in females and males. b, graph showing the d′ values for sex differences in the regions presented in Figure 9. Download Figure 9-1, TIF file. [file enu-eN-NWR-0329-21-s09.tif]
