# Supplementary material for: Brain-Wide Synaptic Inputs to Aromatase-Expressing Neurons in the Medial Amygdala Suggest Complex Circuitry for Modulating Social Behavior
Source: eNeuro. 2022 Mar 11;9(2):ENEURO.0329-21.2021. doi: 10.1523/ENEURO.0329-21.2021 (PMC8925724; doi:10.1523/ENEURO.0329-21.2021)
Supplement: Table 3-1 — Statistical analysis of observed input cells across brain regions. Download Table 3-1, DOCX file. [file enu-eN-NWR-0329-21-s12.docx]

Extended data, Table 3-1: **Statistical analysis of observed input cells across brain regions.** The average percentage of input cells observed in each brain region and the results of two t-tests performed on percentage of cells in each region. 1) T-tests performed to determine if there is any significant input from each brain region. 2) T-tests performed to determine if the input from a given region is larger than expected assuming inputs directly proportional to region volume. The percentage of brain area that each brain region encompasses is also presented for this analysis. Degrees of freedom and standard deviation for each test are also provided.

Superior central nucleus raphe: Mean: 0 +/- 0 SEM

Test against a mean of 0

Superior central nucleus raphe: ttest pval: NaN; tstat : NaN; df : 17; sd : 0

Test against a Expected # of cells based on region size 0.13627

Superior central nucleus raphe: ttest pval (scaled--expected based on size): 1; tstat : -Inf; df : 17; sd : 0

--------------------------

Locus ceruleus: Mean: 0 +/- 0 SEM

Test against a mean of 0

Locus ceruleus: ttest pval: NaN; tstat : NaN; df : 17; sd : 0

Test against a Expected # of cells based on region size 0.0031612

Locus ceruleus: ttest pval (scaled--expected based on size): 1; tstat : -Inf; df : 17; sd : 0

--------------------------

Laterodorsal tegmental nucleus: Mean: 0 +/- 0 SEM

Test against a mean of 0

Laterodorsal tegmental nucleus: ttest pval: NaN; tstat : NaN; df : 17; sd : 0

Test against a Expected # of cells based on region size 0.044372

Laterodorsal tegmental nucleus: ttest pval (scaled--expected based on size): 1; tstat : -Inf; df : 17; sd : 0

--------------------------

Nucleus incertus: Mean: 0 +/- 0 SEM

Test against a mean of 0

Nucleus incertus: ttest pval: NaN; tstat : NaN; df : 17; sd : 0

Test against a Expected # of cells based on region size 0.023008

Nucleus incertus: ttest pval (scaled--expected based on size): 1; tstat : -Inf; df : 17; sd : 0

--------------------------

Pontine reticular nucleus: Mean: 0 +/- 0 SEM

Test against a mean of 0

Pontine reticular nucleus: ttest pval: NaN; tstat : NaN; df : 17; sd : 0

Test against a Expected # of cells based on region size 0.54093

Pontine reticular nucleus: ttest pval (scaled--expected based on size): 1; tstat : -Inf; df : 17; sd : 0

--------------------------

Nucleus raphe pontis: Mean: 0 +/- 0 SEM

Test against a mean of 0

Nucleus raphe pontis: ttest pval: NaN; tstat : NaN; df : 17; sd : 0

Test against a Expected # of cells based on region size 0.020984

Nucleus raphe pontis: ttest pval (scaled--expected based on size): 1; tstat : -Inf; df : 17; sd : 0

--------------------------

Subceruleus nucleus: Mean: 0 +/- 0 SEM

Test against a mean of 0

Subceruleus nucleus: ttest pval: NaN; tstat : NaN; df : 17; sd : 0

Test against a Expected # of cells based on region size 0.0057519

Subceruleus nucleus: ttest pval (scaled--expected based on size): 1; tstat : -Inf; df : 17; sd : 0

--------------------------

Sublaterodorsal nucleus: Mean: 0 +/- 0 SEM

Test against a mean of 0

Sublaterodorsal nucleus: ttest pval: NaN; tstat : NaN; df : 17; sd : 0

Test against a Expected # of cells based on region size 0.010241

Sublaterodorsal nucleus: ttest pval (scaled--expected based on size): 1; tstat : -Inf; df : 17; sd : 0

--------------------------

Nucleus of the lateral lemniscus: Mean: 0.00044941 +/- 0.00011216 SEM

Test against a mean of 0

Nucleus of the lateral lemniscus: ttest pval: 0.16567; tstat : 1; df : 17; sd : 0.0019067

Test against a Expected # of cells based on region size 0.25004

Nucleus of the lateral lemniscus: ttest pval (scaled--expected based on size): 1; tstat : -555.3697; df : 17; sd : 0.0019067

--------------------------

Principal sensory nucleus of the trigeminal: Mean: 0 +/- 0 SEM

Test against a mean of 0

Principal sensory nucleus of the trigeminal: ttest pval: NaN; tstat : NaN; df : 17; sd : 0

Test against a Expected # of cells based on region size 0.24141

Principal sensory nucleus of the trigeminal: ttest pval (scaled--expected based on size): 1; tstat : -Inf; df : 17; sd : 0

--------------------------

parabrachial nucleus: Mean: 0.0017976 +/- 0.00044863 SEM

Test against a mean of 0

parabrachial nucleus: ttest pval: 0.16567; tstat : 1; df : 17; sd : 0.0076267

Test against a Expected # of cells based on region size 0.29638

parabrachial nucleus: ttest pval (scaled--expected based on size): 1; tstat : -163.8736; df : 17; sd : 0.0076267

--------------------------

Superior olivary complex: Mean: 0 +/- 0 SEM

Test against a mean of 0

Superior olivary complex: ttest pval: NaN; tstat : NaN; df : 17; sd : 0

Test against a Expected # of cells based on region size 0.20263

Superior olivary complex: ttest pval (scaled--expected based on size): 1; tstat : -Inf; df : 17; sd : 0

--------------------------

Abducens nucleus: Mean: 0 +/- 0 SEM

Test against a mean of 0

Abducens nucleus: ttest pval: NaN; tstat : NaN; df : 17; sd : 0

Test against a Expected # of cells based on region size 0.0080405

Abducens nucleus: ttest pval (scaled--expected based on size): 1; tstat : -Inf; df : 17; sd : 0

--------------------------

Facial motor nucleus: Mean: 0.00059285 +/- 0.00014795 SEM

Test against a mean of 0

Facial motor nucleus: ttest pval: 0.16567; tstat : 1; df : 17; sd : 0.0025152

Test against a Expected # of cells based on region size 0.20784

Facial motor nucleus: ttest pval (scaled--expected based on size): 1; tstat : -349.5866; df : 17; sd : 0.0025152

--------------------------

Accessory facial motor nucleus: Mean: 0 +/- 0 SEM

Test against a mean of 0

Accessory facial motor nucleus: ttest pval: NaN; tstat : NaN; df : 17; sd : 0

Test against a Expected # of cells based on region size 0.0011986

Accessory facial motor nucleus: ttest pval (scaled--expected based on size): 1; tstat : -Inf; df : 17; sd : 0

--------------------------

Nucleus ambiguus: Mean: 0 +/- 0 SEM

Test against a mean of 0

Nucleus ambiguus: ttest pval: NaN; tstat : NaN; df : 17; sd : 0

Test against a Expected # of cells based on region size 0.0093817

Nucleus ambiguus: ttest pval (scaled--expected based on size): 1; tstat : -Inf; df : 17; sd : 0

--------------------------

Dorsal motor nucleus of the vagus nerve: Mean: 0 +/- 0 SEM

Test against a mean of 0

Dorsal motor nucleus of the vagus nerve: ttest pval: NaN; tstat : NaN; df : 17; sd : 0

Test against a Expected # of cells based on region size 0.037822

Dorsal motor nucleus of the vagus nerve: ttest pval (scaled--expected based on size): 1; tstat : -Inf; df : 17; sd : 0

--------------------------

Gigantocellular reticular nucleus: Mean: 0.003247 +/- 0.00081034 SEM

Test against a mean of 0

Gigantocellular reticular nucleus: ttest pval: 0.16567; tstat : 1; df : 17; sd : 0.013776

Test against a Expected # of cells based on region size 0.60393

Gigantocellular reticular nucleus: ttest pval (scaled--expected based on size): 1; tstat : -184.9991; df : 17; sd : 0.013776

--------------------------

Infracerebellar nucleus: Mean: 0 +/- 0 SEM

Test against a mean of 0

Infracerebellar nucleus: ttest pval: NaN; tstat : NaN; df : 17; sd : 0

Test against a Expected # of cells based on region size 0.011742

Infracerebellar nucleus: ttest pval (scaled--expected based on size): 1; tstat : -Inf; df : 17; sd : 0

--------------------------

Inferior olivary complex: Mean: 0 +/- 0 SEM

Test against a mean of 0

Inferior olivary complex: ttest pval: NaN; tstat : NaN; df : 17; sd : 0

Test against a Expected # of cells based on region size 0.11344

Inferior olivary complex: ttest pval (scaled--expected based on size): 1; tstat : -Inf; df : 17; sd : 0

--------------------------

Intermediate reticular nucleus: Mean: 0 +/- 0 SEM

Test against a mean of 0

Intermediate reticular nucleus: ttest pval: NaN; tstat : NaN; df : 17; sd : 0

Test against a Expected # of cells based on region size 0.60413

Intermediate reticular nucleus: ttest pval (scaled--expected based on size): 1; tstat : -Inf; df : 17; sd : 0

--------------------------

Inferior salivatory nucleus: Mean: 0 +/- 0 SEM

Test against a mean of 0

Inferior salivatory nucleus: ttest pval: NaN; tstat : NaN; df : 17; sd : 0

Test against a Expected # of cells based on region size 0.0015619

Inferior salivatory nucleus: ttest pval (scaled--expected based on size): 1; tstat : -Inf; df : 17; sd : 0

--------------------------

Linear nucleus of the medulla: Mean: 0 +/- 0 SEM

Test against a mean of 0

Linear nucleus of the medulla: ttest pval: NaN; tstat : NaN; df : 17; sd : 0

Test against a Expected # of cells based on region size 0.013405

Linear nucleus of the medulla: ttest pval (scaled--expected based on size): 1; tstat : -Inf; df : 17; sd : 0

--------------------------

Lateral reticular nucleus: Mean: 0 +/- 0 SEM

Test against a mean of 0

Lateral reticular nucleus: ttest pval: NaN; tstat : NaN; df : 17; sd : 0

Test against a Expected # of cells based on region size 0.13335

Lateral reticular nucleus: ttest pval (scaled--expected based on size): 1; tstat : -Inf; df : 17; sd : 0

--------------------------

Magnocellular reticular nucleus: Mean: 0 +/- 0 SEM

Test against a mean of 0

Magnocellular reticular nucleus: ttest pval: NaN; tstat : NaN; df : 17; sd : 0

Test against a Expected # of cells based on region size 0.11764

Magnocellular reticular nucleus: ttest pval (scaled--expected based on size): 1; tstat : -Inf; df : 17; sd : 0

--------------------------

Medullary reticular nucleus: Mean: 0 +/- 0 SEM

Test against a mean of 0

Medullary reticular nucleus: ttest pval: NaN; tstat : NaN; df : 17; sd : 0

Test against a Expected # of cells based on region size 0.44487

Medullary reticular nucleus: ttest pval (scaled--expected based on size): 1; tstat : -Inf; df : 17; sd : 0

--------------------------

Parvicellular reticular nucleus: Mean: 0 +/- 0 SEM

Test against a mean of 0

Parvicellular reticular nucleus: ttest pval: NaN; tstat : NaN; df : 17; sd : 0

Test against a Expected # of cells based on region size 0.49066

Parvicellular reticular nucleus: ttest pval (scaled--expected based on size): 1; tstat : -Inf; df : 17; sd : 0

--------------------------

Parasolitary nucleus: Mean: 0 +/- 0 SEM

Test against a mean of 0

Parasolitary nucleus: ttest pval: NaN; tstat : NaN; df : 17; sd : 0

Test against a Expected # of cells based on region size 0.006139

Parasolitary nucleus: ttest pval (scaled--expected based on size): 1; tstat : -Inf; df : 17; sd : 0

--------------------------

Paragigantocellular reticular nucleus: Mean: 0 +/- 0 SEM

Test against a mean of 0

Paragigantocellular reticular nucleus: ttest pval: NaN; tstat : NaN; df : 17; sd : 0

Test against a Expected # of cells based on region size 0.21407

Paragigantocellular reticular nucleus: ttest pval (scaled--expected based on size): 1; tstat : -Inf; df : 17; sd : 0

--------------------------

Perihypoglossal nuclei: Mean: 0 +/- 0 SEM

Test against a mean of 0

Perihypoglossal nuclei: ttest pval: NaN; tstat : NaN; df : 17; sd : 0

Test against a Expected # of cells based on region size 0.07651

Perihypoglossal nuclei: ttest pval (scaled--expected based on size): 1; tstat : -Inf; df : 17; sd : 0

--------------------------

Parapyramidal nucleus: Mean: 0 +/- 0 SEM

Test against a mean of 0

Parapyramidal nucleus: ttest pval: NaN; tstat : NaN; df : 17; sd : 0

Test against a Expected # of cells based on region size 0.02057

Parapyramidal nucleus: ttest pval (scaled--expected based on size): 1; tstat : -Inf; df : 17; sd : 0

--------------------------

Vestibular nuclei: Mean: 0 +/- 0 SEM

Test against a mean of 0

Vestibular nuclei: ttest pval: NaN; tstat : NaN; df : 17; sd : 0

Test against a Expected # of cells based on region size 0.74321

Vestibular nuclei: ttest pval (scaled--expected based on size): 1; tstat : -Inf; df : 17; sd : 0

--------------------------

Nucleus x: Mean: 0 +/- 0 SEM

Test against a mean of 0

Nucleus x: ttest pval: NaN; tstat : NaN; df : 17; sd : 0

Test against a Expected # of cells based on region size 0.01238

Nucleus x: ttest pval (scaled--expected based on size): 1; tstat : -Inf; df : 17; sd : 0

--------------------------

Hypoglossal nucleus: Mean: 0 +/- 0 SEM

Test against a mean of 0

Hypoglossal nucleus: ttest pval: NaN; tstat : NaN; df : 17; sd : 0

Test against a Expected # of cells based on region size 0.061142

Hypoglossal nucleus: ttest pval (scaled--expected based on size): 1; tstat : -Inf; df : 17; sd : 0

--------------------------

Nucleus y: Mean: 0 +/- 0 SEM

Test against a mean of 0

Nucleus y: ttest pval: NaN; tstat : NaN; df : 17; sd : 0

Test against a Expected # of cells based on region size 0.0050966

Nucleus y: ttest pval (scaled--expected based on size): 1; tstat : -Inf; df : 17; sd : 0

--------------------------

Nucleus raphe magnus: Mean: 0.0010423 +/- 0.00018033 SEM

Test against a mean of 0

Nucleus raphe magnus: ttest pval: 0.083673; tstat : 1.4425; df : 17; sd : 0.0030655

Test against a Expected # of cells based on region size 0.031972

Nucleus raphe magnus: ttest pval (scaled--expected based on size): 1; tstat : -42.8057; df : 17; sd : 0.0030655

--------------------------

Nucleus raphe pallidus: Mean: 0 +/- 0 SEM

Test against a mean of 0

Nucleus raphe pallidus: ttest pval: NaN; tstat : NaN; df : 17; sd : 0

Test against a Expected # of cells based on region size 0.01641

Nucleus raphe pallidus: ttest pval (scaled--expected based on size): 1; tstat : -Inf; df : 17; sd : 0

--------------------------

Nucleus raphe obscurus: Mean: 0 +/- 0 SEM

Test against a mean of 0

Nucleus raphe obscurus: ttest pval: NaN; tstat : NaN; df : 17; sd : 0

Test against a Expected # of cells based on region size 0.013928

Nucleus raphe obscurus: ttest pval (scaled--expected based on size): 1; tstat : -Inf; df : 17; sd : 0

--------------------------

Area postrema: Mean: 0 +/- 0 SEM

Test against a mean of 0

Area postrema: ttest pval: NaN; tstat : NaN; df : 17; sd : 0

Test against a Expected # of cells based on region size 0.012733

Area postrema: ttest pval (scaled--expected based on size): 1; tstat : -Inf; df : 17; sd : 0

--------------------------

Cochlear nuclei: Mean: 0 +/- 0 SEM

Test against a mean of 0

Cochlear nuclei: ttest pval: NaN; tstat : NaN; df : 17; sd : 0

Test against a Expected # of cells based on region size 0.41049

Cochlear nuclei: ttest pval (scaled--expected based on size): 1; tstat : -Inf; df : 17; sd : 0

--------------------------

Dorsal column nuclei: Mean: 0 +/- 0 SEM

Test against a mean of 0

Dorsal column nuclei: ttest pval: NaN; tstat : NaN; df : 17; sd : 0

Test against a Expected # of cells based on region size 0.09435

Dorsal column nuclei: ttest pval (scaled--expected based on size): 1; tstat : -Inf; df : 17; sd : 0

--------------------------

External cuneate nucleus: Mean: 0 +/- 0 SEM

Test against a mean of 0

External cuneate nucleus: ttest pval: NaN; tstat : NaN; df : 17; sd : 0

Test against a Expected # of cells based on region size 0.052097

External cuneate nucleus: ttest pval (scaled--expected based on size): 1; tstat : -Inf; df : 17; sd : 0

--------------------------

Nucleus of the trapezoid body: Mean: 0 +/- 0 SEM

Test against a mean of 0

Nucleus of the trapezoid body: ttest pval: NaN; tstat : NaN; df : 17; sd : 0

Test against a Expected # of cells based on region size 0.0397

Nucleus of the trapezoid body: ttest pval (scaled--expected based on size): 1; tstat : -Inf; df : 17; sd : 0

--------------------------

Nucleus of the solitary tract: Mean: 0 +/- 0 SEM

Test against a mean of 0

Nucleus of the solitary tract: ttest pval: NaN; tstat : NaN; df : 17; sd : 0

Test against a Expected # of cells based on region size 0.19355

Nucleus of the solitary tract: ttest pval (scaled--expected based on size): 1; tstat : -Inf; df : 17; sd : 0

--------------------------

Spinal nucleus of the trigeminal, caudal part: Mean: 0 +/- 0 SEM

Test against a mean of 0

Spinal nucleus of the trigeminal, caudal part: ttest pval: NaN; tstat : NaN; df : 17; sd : 0

Test against a Expected # of cells based on region size 0.3686

Spinal nucleus of the trigeminal, caudal part: ttest pval (scaled--expected based on size): 1; tstat : -Inf; df : 17; sd : 0

--------------------------

Spinal nucleus of the trigeminal, interpolar part: Mean: 0 +/- 0 SEM

Test against a mean of 0

Spinal nucleus of the trigeminal, interpolar part: ttest pval: NaN; tstat : NaN; df : 17; sd : 0

Test against a Expected # of cells based on region size 0.4143

Spinal nucleus of the trigeminal, interpolar part: ttest pval (scaled--expected based on size): 1; tstat : -Inf; df : 17; sd : 0

--------------------------

Spinal nucleus of the trigeminal, oral part: Mean: 0 +/- 0 SEM

Test against a mean of 0

Spinal nucleus of the trigeminal, oral part: ttest pval: NaN; tstat : NaN; df : 17; sd : 0

Test against a Expected # of cells based on region size 0.21959

Spinal nucleus of the trigeminal, oral part: ttest pval (scaled--expected based on size): 1; tstat : -Inf; df : 17; sd : 0

--------------------------

Paratrigeminal nucleus: Mean: 0 +/- 0 SEM

Test against a mean of 0

Paratrigeminal nucleus: ttest pval: NaN; tstat : NaN; df : 17; sd : 0

Test against a Expected # of cells based on region size 0.029041

Paratrigeminal nucleus: ttest pval (scaled--expected based on size): 1; tstat : -Inf; df : 17; sd : 0

--------------------------

Barrington's nucleus: Mean: 0 +/- 0 SEM

Test against a mean of 0

Barrington's nucleus: ttest pval: NaN; tstat : NaN; df : 17; sd : 0

Test against a Expected # of cells based on region size 0.0033751

Barrington's nucleus: ttest pval (scaled--expected based on size): 1; tstat : -Inf; df : 17; sd : 0

--------------------------

Dorsal tegmental nucleus: Mean: 0 +/- 0 SEM

Test against a mean of 0

Dorsal tegmental nucleus: ttest pval: NaN; tstat : NaN; df : 17; sd : 0

Test against a Expected # of cells based on region size 0.022536

Dorsal tegmental nucleus: ttest pval (scaled--expected based on size): 1; tstat : -Inf; df : 17; sd : 0

--------------------------

Posterodorsal tegmental nucleus: Mean: 0 +/- 0 SEM

Test against a mean of 0

Posterodorsal tegmental nucleus: ttest pval: NaN; tstat : NaN; df : 17; sd : 0

Test against a Expected # of cells based on region size 0.010397

Posterodorsal tegmental nucleus: ttest pval (scaled--expected based on size): 1; tstat : -Inf; df : 17; sd : 0

--------------------------

Pontine central gray: Mean: 0 +/- 0 SEM

Test against a mean of 0

Pontine central gray: ttest pval: NaN; tstat : NaN; df : 17; sd : 0

Test against a Expected # of cells based on region size 0.14153

Pontine central gray: ttest pval (scaled--expected based on size): 1; tstat : -Inf; df : 17; sd : 0

--------------------------

Pontine gray: Mean: 0 +/- 0 SEM

Test against a mean of 0

Pontine gray: ttest pval: NaN; tstat : NaN; df : 17; sd : 0

Test against a Expected # of cells based on region size 0.21287

Pontine gray: ttest pval (scaled--expected based on size): 1; tstat : -Inf; df : 17; sd : 0

--------------------------

Pontine reticular nucleus, caudal part: Mean: 0.003247 +/- 0.00081034 SEM

Test against a mean of 0

Pontine reticular nucleus, caudal part: ttest pval: 0.16567; tstat : 1; df : 17; sd : 0.013776

Test against a Expected # of cells based on region size 0.54369

Pontine reticular nucleus, caudal part: ttest pval (scaled--expected based on size): 1; tstat : -166.4446; df : 17; sd : 0.013776

--------------------------

Supragenual nucleus: Mean: 0 +/- 0 SEM

Test against a mean of 0

Supragenual nucleus: ttest pval: NaN; tstat : NaN; df : 17; sd : 0

Test against a Expected # of cells based on region size 0.0037724

Supragenual nucleus: ttest pval (scaled--expected based on size): 1; tstat : -Inf; df : 17; sd : 0

--------------------------

Supratrigeminal nucleus: Mean: 0 +/- 0 SEM

Test against a mean of 0

Supratrigeminal nucleus: ttest pval: NaN; tstat : NaN; df : 17; sd : 0

Test against a Expected # of cells based on region size 0.056501

Supratrigeminal nucleus: ttest pval (scaled--expected based on size): 1; tstat : -Inf; df : 17; sd : 0

--------------------------

Tegmental reticular nucleus: Mean: 0 +/- 0 SEM

Test against a mean of 0

Tegmental reticular nucleus: ttest pval: NaN; tstat : NaN; df : 17; sd : 0

Test against a Expected # of cells based on region size 0.18094

Tegmental reticular nucleus: ttest pval (scaled--expected based on size): 1; tstat : -Inf; df : 17; sd : 0

--------------------------

Motor nucleus of trigeminal: Mean: 0 +/- 0 SEM

Test against a mean of 0

Motor nucleus of trigeminal: ttest pval: NaN; tstat : NaN; df : 17; sd : 0

Test against a Expected # of cells based on region size 0.07705

Motor nucleus of trigeminal: ttest pval (scaled--expected based on size): 1; tstat : -Inf; df : 17; sd : 0

--------------------------

Peritrigeminal zone: Mean: 0 +/- 0 SEM

Test against a mean of 0

Peritrigeminal zone: ttest pval: NaN; tstat : NaN; df : 17; sd : 0

Test against a Expected # of cells based on region size 0.069662

Peritrigeminal zone: ttest pval (scaled--expected based on size): 1; tstat : -Inf; df : 17; sd : 0

--------------------------

Accessory trigeminal nucleus: Mean: 0 +/- 0 SEM

Test against a mean of 0

Accessory trigeminal nucleus: ttest pval: NaN; tstat : NaN; df : 17; sd : 0

Test against a Expected # of cells based on region size 0.0031035

Accessory trigeminal nucleus: ttest pval (scaled--expected based on size): 1; tstat : -Inf; df : 17; sd : 0

--------------------------

Parvicellular motor 5 nucleus: Mean: 0 +/- 0 SEM

Test against a mean of 0

Parvicellular motor 5 nucleus: ttest pval: NaN; tstat : NaN; df : 17; sd : 0

Test against a Expected # of cells based on region size 0.018285

Parvicellular motor 5 nucleus: ttest pval (scaled--expected based on size): 1; tstat : -Inf; df : 17; sd : 0

--------------------------

Intertrigeminal nucleus: Mean: 0 +/- 0 SEM

Test against a mean of 0

Intertrigeminal nucleus: ttest pval: NaN; tstat : NaN; df : 17; sd : 0

Test against a Expected # of cells based on region size 0.0095718

Intertrigeminal nucleus: ttest pval (scaled--expected based on size): 1; tstat : -Inf; df : 17; sd : 0

--------------------------

Field CA1: Mean: 2.4885 +/- 0.18631 SEM

Test against a mean of 0

Field CA1: ttest pval: 0.0019677; tstat : 3.3334; df : 17; sd : 3.1672

Test against a Expected # of cells based on region size 2.6586

Field CA1: ttest pval (scaled--expected based on size): 0.58877; tstat : -0.22787; df : 17; sd : 3.1672

--------------------------

Field CA2: Mean: 0.25768 +/- 0.023006 SEM

Test against a mean of 0

Field CA2: ttest pval: 0.0062132; tstat : 2.7953; df : 17; sd : 0.3911

Test against a Expected # of cells based on region size 0.12892

Field CA2: ttest pval (scaled--expected based on size): 0.090217; tstat : 1.3968; df : 17; sd : 0.3911

--------------------------

FieldCa3: Mean: 6.3221 +/- 0.57746 SEM

Test against a mean of 0

FieldCa3: ttest pval: 0.0070927; tstat : 2.7323; df : 17; sd : 9.8169

Test against a Expected # of cells based on region size 1.8646

FieldCa3: ttest pval (scaled--expected based on size): 0.035471; tstat : 1.9264; df : 17; sd : 9.8169

--------------------------

Dentate Gyrus: Mean: 0.018308 +/- 0.0028243 SEM

Test against a mean of 0

Dentate Gyrus: ttest pval: 0.06206; tstat : 1.6177; df : 17; sd : 0.048013

Test against a Expected # of cells based on region size 1.5904

Dentate Gyrus: ttest pval (scaled--expected based on size): 1; tstat : -138.9174; df : 17; sd : 0.048013

--------------------------

Fasciola cinerea: Mean: 0 +/- 0 SEM

Test against a mean of 0

Fasciola cinerea: ttest pval: NaN; tstat : NaN; df : 17; sd : 0

Test against a Expected # of cells based on region size 0.051896

Fasciola cinerea: ttest pval (scaled--expected based on size): 1; tstat : -Inf; df : 17; sd : 0

--------------------------

Induseum griseum: Mean: 0 +/- 0 SEM

Test against a mean of 0

Induseum griseum: ttest pval: NaN; tstat : NaN; df : 17; sd : 0

Test against a Expected # of cells based on region size 0.052127

Induseum griseum: ttest pval (scaled--expected based on size): 1; tstat : -Inf; df : 17; sd : 0

--------------------------

Entorhinal area: Mean: 0.25717 +/- 0.026799 SEM

Test against a mean of 0

Entorhinal area: ttest pval: 0.014209; tstat : 2.3949; df : 17; sd : 0.45559

Test against a Expected # of cells based on region size 2.4996

Entorhinal area: ttest pval (scaled--expected based on size): 1; tstat : -20.8826; df : 17; sd : 0.45559

--------------------------

Parasubiculum: Mean: 0 +/- 0 SEM

Test against a mean of 0

Parasubiculum: ttest pval: NaN; tstat : NaN; df : 17; sd : 0

Test against a Expected # of cells based on region size 0.20365

Parasubiculum: ttest pval (scaled--expected based on size): 1; tstat : -Inf; df : 17; sd : 0

--------------------------

Postsubiculum: Mean: 0 +/- 0 SEM

Test against a mean of 0

Postsubiculum: ttest pval: NaN; tstat : NaN; df : 17; sd : 0

Test against a Expected # of cells based on region size 0.26263

Postsubiculum: ttest pval (scaled--expected based on size): 1; tstat : -Inf; df : 17; sd : 0

--------------------------

Presubiculum: Mean: 0.0087044 +/- 0.0014084 SEM

Test against a mean of 0

Presubiculum: ttest pval: 0.070691; tstat : 1.5424; df : 17; sd : 0.023943

Test against a Expected # of cells based on region size 0.22907

Presubiculum: ttest pval (scaled--expected based on size): 1; tstat : -39.0475; df : 17; sd : 0.023943

--------------------------

Subiculum: Mean: 0.0026964 +/- 0.00067294 SEM

Test against a mean of 0

Subiculum: ttest pval: 0.16567; tstat : 1; df : 17; sd : 0.01144

Test against a Expected # of cells based on region size 0.56318

Subiculum: ttest pval (scaled--expected based on size): 1; tstat : -207.8597; df : 17; sd : 0.01144

--------------------------

Prosubiculum: Mean: 0.09977 +/- 0.012787 SEM

Test against a mean of 0

Prosubiculum: ttest pval: 0.034105; tstat : 1.9473; df : 17; sd : 0.21737

Test against a Expected # of cells based on region size 0.31964

Prosubiculum: ttest pval (scaled--expected based on size): 0.99975; tstat : -4.2914; df : 17; sd : 0.21737

--------------------------

Hippocampo-amygdalar transition area: Mean: 0.21843 +/- 0.020456 SEM

Test against a mean of 0

Hippocampo-amygdalar transition area: ttest pval: 0.0081639; tstat : 2.6649; df : 17; sd : 0.34776

Test against a Expected # of cells based on region size 0.092798

Hippocampo-amygdalar transition area: ttest pval (scaled--expected based on size): 0.07187; tstat : 1.5327; df : 17; sd : 0.34776

--------------------------

Area prostriata: Mean: 0 +/- 0 SEM

Test against a mean of 0

Area prostriata: ttest pval: NaN; tstat : NaN; df : 17; sd : 0

Test against a Expected # of cells based on region size 0.071492

Area prostriata: ttest pval (scaled--expected based on size): 1; tstat : -Inf; df : 17; sd : 0

--------------------------

Median eminence: Mean: 0 +/- 0 SEM

Test against a mean of 0

Median eminence: ttest pval: NaN; tstat : NaN; df : 17; sd : 0

Test against a Expected # of cells based on region size 0.018196

Median eminence: ttest pval (scaled--expected based on size): 1; tstat : -Inf; df : 17; sd : 0

--------------------------

Anterodorsal preoptic nucleus: Mean: 0.015989 +/- 0.0016003 SEM

Test against a mean of 0

Anterodorsal preoptic nucleus: ttest pval: 0.01163; tstat : 2.4934; df : 17; sd : 0.027206

Test against a Expected # of cells based on region size 0.021989

Anterodorsal preoptic nucleus: ttest pval (scaled--expected based on size): 0.81873; tstat : -0.93569; df : 17; sd : 0.027206

--------------------------

Anteroventral preoptic nucleus: Mean: 0.070683 +/- 0.0059333 SEM

Test against a mean of 0

Anteroventral preoptic nucleus: ttest pval: 0.0042651; tstat : 2.9731; df : 17; sd : 0.10087

Test against a Expected # of cells based on region size 0.02003

Anteroventral preoptic nucleus: ttest pval (scaled--expected based on size): 0.024012; tstat : 2.1306; df : 17; sd : 0.10087

--------------------------

Anteroventral periventricular nucleus: Mean: 0.051614 +/- 0.0055466 SEM

Test against a mean of 0

Anteroventral periventricular nucleus: ttest pval: 0.016441; tstat : 2.3224; df : 17; sd : 0.094292

Test against a Expected # of cells based on region size 0.070762

Anteroventral periventricular nucleus: ttest pval (scaled--expected based on size): 0.79953; tstat : -0.86153; df : 17; sd : 0.094292

--------------------------

Dorsomedial nucleus of the hypothalamus: Mean: 0.4397 +/- 0.047233 SEM

Test against a mean of 0

Dorsomedial nucleus of the hypothalamus: ttest pval: 0.016412; tstat : 2.3233; df : 17; sd : 0.80295

Test against a Expected # of cells based on region size 0.081722

Dorsomedial nucleus of the hypothalamus: ttest pval (scaled--expected based on size): 0.037863; tstat : 1.8915; df : 17; sd : 0.80295

--------------------------

Median preoptic nucleus: Mean: 0.001908 +/- 0.00032727 SEM

Test against a mean of 0

Median preoptic nucleus: ttest pval: 0.081943; tstat : 1.455; df : 17; sd : 0.0055635

Test against a Expected # of cells based on region size 0.01748

Median preoptic nucleus: ttest pval (scaled--expected based on size): 1; tstat : -11.8748; df : 17; sd : 0.0055635

--------------------------

Medial preoptic area: Mean: 0.25786 +/- 0.019784 SEM

Test against a mean of 0

Medial preoptic area: ttest pval: 0.0023415; tstat : 3.2528; df : 17; sd : 0.33633

Test against a Expected # of cells based on region size 0.12125

Medial preoptic area: ttest pval (scaled--expected based on size): 0.051482; tstat : 1.7233; df : 17; sd : 0.33633

--------------------------

Vascular organ of the lamina terminalis: Mean: 0 +/- 0 SEM

Test against a mean of 0

Vascular organ of the lamina terminalis: ttest pval: NaN; tstat : NaN; df : 17; sd : 0

Test against a Expected # of cells based on region size 0.0030865

Vascular organ of the lamina terminalis: ttest pval (scaled--expected based on size): 1; tstat : -Inf; df : 17; sd : 0

--------------------------

Posterodorsal preoptic nucleus: Mean: 0.0086461 +/- 0.00093346 SEM

Test against a mean of 0

Posterodorsal preoptic nucleus: ttest pval: 0.0168; tstat : 2.3116; df : 17; sd : 0.015869

Test against a Expected # of cells based on region size 0.0025228

Posterodorsal preoptic nucleus: ttest pval (scaled--expected based on size): 0.059994; tstat : 1.6371; df : 17; sd : 0.015869

--------------------------

Parastrial nucleus: Mean: 0.012037 +/- 0.0015809 SEM

Test against a mean of 0

Parastrial nucleus: ttest pval: 0.037248; tstat : 1.9003; df : 17; sd : 0.026875

Test against a Expected # of cells based on region size 0.02096

Parastrial nucleus: ttest pval (scaled--expected based on size): 0.91151; tstat : -1.4086; df : 17; sd : 0.026875

--------------------------

Periventricular hypothalamic nucleus, posterior part: Mean: 0.069225 +/- 0.0081592 SEM

Test against a mean of 0

Periventricular hypothalamic nucleus, posterior part: ttest pval: 0.024635; tstat : 2.1174; df : 17; sd : 0.13871

Test against a Expected # of cells based on region size 0.031252

Periventricular hypothalamic nucleus, posterior part: ttest pval (scaled--expected based on size): 0.13074; tstat : 1.1615; df : 17; sd : 0.13871

--------------------------

Periventricular hypothalamic nucleus, preoptic part: Mean: 0.035883 +/- 0.0042756 SEM

Test against a mean of 0

Periventricular hypothalamic nucleus, preoptic part: ttest pval: 0.025751; tstat : 2.0945; df : 17; sd : 0.072685

Test against a Expected # of cells based on region size 0.045852

Periventricular hypothalamic nucleus, preoptic part: ttest pval (scaled--expected based on size): 0.71587; tstat : -0.5819; df : 17; sd : 0.072685

--------------------------

Subparaventricular zone: Mean: 0.0041656 +/- 0.00060095 SEM

Test against a mean of 0

Subparaventricular zone: ttest pval: 0.050878; tstat : 1.7299; df : 17; sd : 0.010216

Test against a Expected # of cells based on region size 0.023795

Subparaventricular zone: ttest pval (scaled--expected based on size): 1; tstat : -8.152; df : 17; sd : 0.010216

--------------------------

Suprachiasmatic nucleus: Mean: 0.0046598 +/- 0.0010502 SEM

Test against a mean of 0

Suprachiasmatic nucleus: ttest pval: 0.14178; tstat : 1.1074; df : 17; sd : 0.017853

Test against a Expected # of cells based on region size 0.02074

Suprachiasmatic nucleus: ttest pval (scaled--expected based on size): 0.99932; tstat : -3.8213; df : 17; sd : 0.017853

--------------------------

Subfornical organ: Mean: 0.0011857 +/- 0.00029591 SEM

Test against a mean of 0

Subfornical organ: ttest pval: 0.16567; tstat : 1; df : 17; sd : 0.0050305

Test against a Expected # of cells based on region size 0.025211

Subfornical organ: ttest pval (scaled--expected based on size): 1; tstat : -20.263; df : 17; sd : 0.0050305

--------------------------

Ventromedial preoptic nucleus: Mean: 0.0059082 +/- 0.0010758 SEM

Test against a mean of 0

Ventromedial preoptic nucleus: ttest pval: 0.094157; tstat : 1.3706; df : 17; sd : 0.018288

Test against a Expected # of cells based on region size 0.023541

Ventromedial preoptic nucleus: ttest pval (scaled--expected based on size): 0.99962; tstat : -4.0906; df : 17; sd : 0.018288

--------------------------

Ventrolateral preoptic nucleus: Mean: 0.012442 +/- 0.0026021 SEM

Test against a mean of 0

Ventrolateral preoptic nucleus: ttest pval: 0.12458; tstat : 1.1933; df : 17; sd : 0.044236

Test against a Expected # of cells based on region size 0.02605

Ventrolateral preoptic nucleus: ttest pval (scaled--expected based on size): 0.89539; tstat : -1.3052; df : 17; sd : 0.044236

--------------------------

Supraoptic nucleus: Mean: 0.0081396 +/- 0.0014333 SEM

Test against a mean of 0

Supraoptic nucleus: ttest pval: 0.087242; tstat : 1.4172; df : 17; sd : 0.024367

Test against a Expected # of cells based on region size 0.030766

Supraoptic nucleus: ttest pval (scaled--expected based on size): 0.99947; tstat : -3.9397; df : 17; sd : 0.024367

--------------------------

Accessory supraoptic group: Mean: 0.0012484 +/- 0.00031157 SEM

Test against a mean of 0

Accessory supraoptic group: ttest pval: 0.16567; tstat : 1; df : 17; sd : 0.0052967

Test against a Expected # of cells based on region size 0.0013174

Accessory supraoptic group: ttest pval (scaled--expected based on size): 0.52172; tstat : -0.055272; df : 17; sd : 0.0052967

--------------------------

Paraventricular hypothalamic nucleus: Mean: 0.03159 +/- 0.0059345 SEM

Test against a mean of 0

Paraventricular hypothalamic nucleus: ttest pval: 0.10079; tstat : 1.3285; df : 17; sd : 0.10089

Test against a Expected # of cells based on region size 0.044498

Paraventricular hypothalamic nucleus: ttest pval (scaled--expected based on size): 0.70285; tstat : -0.54283; df : 17; sd : 0.10089

--------------------------

Periventricular hypothalamic nucleus, anterior part: Mean: 0.0051395 +/- 0.00090238 SEM

Test against a mean of 0

Periventricular hypothalamic nucleus, anterior part: ttest pval: 0.086644; tstat : 1.4214; df : 17; sd : 0.015341

Test against a Expected # of cells based on region size 0.014353

Periventricular hypothalamic nucleus, anterior part: ttest pval (scaled--expected based on size): 0.9896; tstat : -2.548; df : 17; sd : 0.015341

--------------------------

Periventricular hypothalamic nucleus, intermediate part: Mean: 0.082574 +/- 0.0049426 SEM

Test against a mean of 0

Periventricular hypothalamic nucleus, intermediate part: ttest pval: 0.00032136; tstat : 4.1694; df : 17; sd : 0.084025

Test against a Expected # of cells based on region size 0.056755

Periventricular hypothalamic nucleus, intermediate part: ttest pval (scaled--expected based on size): 0.10487; tstat : 1.3037; df : 17; sd : 0.084025

--------------------------

Arcuate hypothalamic nucleus: Mean: 0.12561 +/- 0.023991 SEM

Test against a mean of 0

Arcuate hypothalamic nucleus: ttest pval: 0.10435; tstat : 1.3067; df : 17; sd : 0.40784

Test against a Expected # of cells based on region size 0.066881

Arcuate hypothalamic nucleus: ttest pval (scaled--expected based on size): 0.27464; tstat : 0.61099; df : 17; sd : 0.40784

--------------------------

Lateral hypothalamic area: Mean: 2.007 +/- 0.16859 SEM

Test against a mean of 0

Lateral hypothalamic area: ttest pval: 0.0042832; tstat : 2.9711; df : 17; sd : 2.866

Test against a Expected # of cells based on region size 0.47743

Lateral hypothalamic area: ttest pval (scaled--expected based on size): 0.018459; tstat : 2.2643; df : 17; sd : 2.866

--------------------------

Lateral preoptic area: Mean: 0.35671 +/- 0.022095 SEM

Test against a mean of 0

Lateral preoptic area: ttest pval: 0.00043528; tstat : 4.0291; df : 17; sd : 0.37562

Test against a Expected # of cells based on region size 0.12063

Lateral preoptic area: ttest pval (scaled--expected based on size): 0.0081346; tstat : 2.6666; df : 17; sd : 0.37562

--------------------------

Preparasubthalamic nucleus: Mean: 0.017012 +/- 0.0018394 SEM

Test against a mean of 0

Preparasubthalamic nucleus: ttest pval: 0.016913; tstat : 2.3082; df : 17; sd : 0.031269

Test against a Expected # of cells based on region size 0.0039048

Preparasubthalamic nucleus: ttest pval (scaled--expected based on size): 0.04661; tstat : 1.7784; df : 17; sd : 0.031269

--------------------------

Parasubthalamic nucleus: Mean: 0.29958 +/- 0.034813 SEM

Test against a mean of 0

Parasubthalamic nucleus: ttest pval: 0.023226; tstat : 2.1477; df : 17; sd : 0.59182

Test against a Expected # of cells based on region size 0.041815

Parasubthalamic nucleus: ttest pval (scaled--expected based on size): 0.041046; tstat : 1.8479; df : 17; sd : 0.59182

--------------------------

Perifornical nucleus: Mean: 0.065641 +/- 0.0075548 SEM

Test against a mean of 0

Perifornical nucleus: ttest pval: 0.022304; tstat : 2.1684; df : 17; sd : 0.12843

Test against a Expected # of cells based on region size 0.054823

Perifornical nucleus: ttest pval (scaled--expected based on size): 0.36262; tstat : 0.35734; df : 17; sd : 0.12843

--------------------------

Retrochiasmatic area: Mean: 0.093526 +/- 0.023105 SEM

Test against a mean of 0

Retrochiasmatic area: ttest pval: 0.16327; tstat : 1.0102; df : 17; sd : 0.39278

Test against a Expected # of cells based on region size 0.055445

Retrochiasmatic area: ttest pval (scaled--expected based on size): 0.34298; tstat : 0.41134; df : 17; sd : 0.39278

--------------------------

Subthalamic nucleus: Mean: 0.31615 +/- 0.033088 SEM

Test against a mean of 0

Subthalamic nucleus: ttest pval: 0.01451; tstat : 2.3845; df : 17; sd : 0.5625

Test against a Expected # of cells based on region size 0.088897

Subthalamic nucleus: ttest pval (scaled--expected based on size): 0.052349; tstat : 1.714; df : 17; sd : 0.5625

--------------------------

Tuberal nucleus: Mean: 0.88995 +/- 0.21065 SEM

Test against a mean of 0

Tuberal nucleus: ttest pval: 0.15323; tstat : 1.0544; df : 17; sd : 3.581

Test against a Expected # of cells based on region size 0.14193

Tuberal nucleus: ttest pval (scaled--expected based on size): 0.19393; tstat : 0.88623; df : 17; sd : 3.581

--------------------------

Zona incerta: Mean: 0.43925 +/- 0.044104 SEM

Test against a mean of 0

Zona incerta: ttest pval: 0.011818; tstat : 2.4856; df : 17; sd : 0.74977

Test against a Expected # of cells based on region size 0.41362

Zona incerta: ttest pval (scaled--expected based on size): 0.44319; tstat : 0.14505; df : 17; sd : 0.74977

--------------------------

Anterior hypothalamic nucleus: Mean: 0.16055 +/- 0.010375 SEM

Test against a mean of 0

Anterior hypothalamic nucleus: ttest pval: 0.00062553; tstat : 3.8619; df : 17; sd : 0.17638

Test against a Expected # of cells based on region size 0.15438

Anterior hypothalamic nucleus: ttest pval (scaled--expected based on size): 0.44188; tstat : 0.14841; df : 17; sd : 0.17638

--------------------------

Lateral mammillary nucleus: Mean: 0.011553 +/- 0.0016939 SEM

Test against a mean of 0

Lateral mammillary nucleus: ttest pval: 0.053473; tstat : 1.7021; df : 17; sd : 0.028796

Test against a Expected # of cells based on region size 0.020234

Lateral mammillary nucleus: ttest pval (scaled--expected based on size): 0.89096; tstat : -1.279; df : 17; sd : 0.028796

--------------------------

Medial mammillary nucleus: Mean: 0.39412 +/- 0.050426 SEM

Test against a mean of 0

Medial mammillary nucleus: ttest pval: 0.033895; tstat : 1.9506; df : 17; sd : 0.85724

Test against a Expected # of cells based on region size 0.13169

Medial mammillary nucleus: ttest pval (scaled--expected based on size): 0.10567; tstat : 1.2988; df : 17; sd : 0.85724

--------------------------

Supramammillary nucleus: Mean: 0.074357 +/- 0.010899 SEM

Test against a mean of 0

Supramammillary nucleus: ttest pval: 0.053429; tstat : 1.7026; df : 17; sd : 0.18529

Test against a Expected # of cells based on region size 0.066235

Supramammillary nucleus: ttest pval (scaled--expected based on size): 0.42733; tstat : 0.18597; df : 17; sd : 0.18529

--------------------------

Tuberomammillary nucleus: Mean: 0.081479 +/- 0.010209 SEM

Test against a mean of 0

Tuberomammillary nucleus: ttest pval: 0.031348; tstat : 1.9919; df : 17; sd : 0.17355

Test against a Expected # of cells based on region size 0.040192

Tuberomammillary nucleus: ttest pval (scaled--expected based on size): 0.16348; tstat : 1.0093; df : 17; sd : 0.17355

--------------------------

Medial preoptic nucleus: Mean: 0.27183 +/- 0.032809 SEM

Test against a mean of 0

Medial preoptic nucleus: ttest pval: 0.027118; tstat : 2.0677; df : 17; sd : 0.55775

Test against a Expected # of cells based on region size 0.087929

Medial preoptic nucleus: ttest pval (scaled--expected based on size): 0.089916; tstat : 1.3989; df : 17; sd : 0.55775

--------------------------

Dorsal premammillary nucleus: Mean: 0.23528 +/- 0.032657 SEM

Test against a mean of 0

Dorsal premammillary nucleus: ttest pval: 0.044981; tstat : 1.798; df : 17; sd : 0.55517

Test against a Expected # of cells based on region size 0.028634

Dorsal premammillary nucleus: ttest pval (scaled--expected based on size): 0.066362; tstat : 1.5792; df : 17; sd : 0.55517

--------------------------

Ventral premammillary nucleus: Mean: 0.49484 +/- 0.11132 SEM

Test against a mean of 0

Ventral premammillary nucleus: ttest pval: 0.14135; tstat : 1.1094; df : 17; sd : 1.8924

Test against a Expected # of cells based on region size 0.043666

Ventral premammillary nucleus: ttest pval (scaled--expected based on size): 0.16298; tstat : 1.0115; df : 17; sd : 1.8924

--------------------------

Paraventricular hypothalamic nucleus, descending division: Mean: 0.013922 +/- 0.0012852 SEM

Test against a mean of 0

Paraventricular hypothalamic nucleus, descending division: ttest pval: 0.0075325; tstat : 2.7035; df : 17; sd : 0.021848

Test against a Expected # of cells based on region size 0.028634

Paraventricular hypothalamic nucleus, descending division: ttest pval (scaled--expected based on size): 0.99454; tstat : -2.8569; df : 17; sd : 0.021848

--------------------------

Ventromedial hypothalamic nucleus: Mean: 1.2151 +/- 0.16121 SEM

Test against a mean of 0

Ventromedial hypothalamic nucleus: ttest pval: 0.038606; tstat : 1.881; df : 17; sd : 2.7406

Test against a Expected # of cells based on region size 0.11942

Ventromedial hypothalamic nucleus: ttest pval (scaled--expected based on size): 0.054046; tstat : 1.6962; df : 17; sd : 2.7406

--------------------------

Posterior hypothalamic nucleus: Mean: 0.13119 +/- 0.011118 SEM

Test against a mean of 0

Posterior hypothalamic nucleus: ttest pval: 0.0045287; tstat : 2.9449; df : 17; sd : 0.189

Test against a Expected # of cells based on region size 0.15853

Posterior hypothalamic nucleus: ttest pval (scaled--expected based on size): 0.72627; tstat : -0.61383; df : 17; sd : 0.189

--------------------------

Substantia nigra, reticular part: Mean: 0.037884 +/- 0.0052302 SEM

Test against a mean of 0

Substantia nigra, reticular part: ttest pval: 0.044192; tstat : 1.8077; df : 17; sd : 0.088913

Test against a Expected # of cells based on region size 0.43287

Substantia nigra, reticular part: ttest pval (scaled--expected based on size): 1; tstat : -18.8472; df : 17; sd : 0.088913

--------------------------

Ventral tegmental area: Mean: 0.045944 +/- 0.0064395 SEM

Test against a mean of 0

Ventral tegmental area: ttest pval: 0.046427; tstat : 1.7806; df : 17; sd : 0.10947

Test against a Expected # of cells based on region size 0.11082

Ventral tegmental area: ttest pval (scaled--expected based on size): 0.98886; tstat : -2.5143; df : 17; sd : 0.10947

--------------------------

Paranigral nucleus: Mean: 0 +/- 0 SEM

Test against a mean of 0

Paranigral nucleus: ttest pval: NaN; tstat : NaN; df : 17; sd : 0

Test against a Expected # of cells based on region size 0.004774

Paranigral nucleus: ttest pval (scaled--expected based on size): 1; tstat : -Inf; df : 17; sd : 0

--------------------------

Midbrain reticular nucleus, retrorubral area: Mean: 0.0012484 +/- 0.00031157 SEM

Test against a mean of 0

Midbrain reticular nucleus, retrorubral area: ttest pval: 0.16567; tstat : 1; df : 17; sd : 0.0052967

Test against a Expected # of cells based on region size 0.030233

Midbrain reticular nucleus, retrorubral area: ttest pval (scaled--expected based on size): 1; tstat : -23.2169; df : 17; sd : 0.0052967

--------------------------

Midbrain reticular nucleus: Mean: 0.10224 +/- 0.018492 SEM

Test against a mean of 0

Midbrain reticular nucleus: ttest pval: 0.092747; tstat : 1.3799; df : 17; sd : 0.31436

Test against a Expected # of cells based on region size 1.1584

Midbrain reticular nucleus: ttest pval (scaled--expected based on size): 1; tstat : -14.2545; df : 17; sd : 0.31436

--------------------------

Superior colliculus, motor related: Mean: 0.13275 +/- 0.022439 SEM

Test against a mean of 0

Superior colliculus, motor related: ttest pval: 0.079047; tstat : 1.4765; df : 17; sd : 0.38146

Test against a Expected # of cells based on region size 1.2407

Superior colliculus, motor related: ttest pval (scaled--expected based on size): 1; tstat : -12.3225; df : 17; sd : 0.38146

--------------------------

Periaqueductal gray: Mean: 0.0050446 +/- 0.00090285 SEM

Test against a mean of 0

Periaqueductal gray: ttest pval: 0.090573; tstat : 1.3944; df : 17; sd : 0.015348

Test against a Expected # of cells based on region size 1.0825

Periaqueductal gray: ttest pval (scaled--expected based on size): 1; tstat : -297.8342; df : 17; sd : 0.015348

--------------------------

Pretectal region: Mean: 0.00044941 +/- 0.00011216 SEM

Test against a mean of 0

Pretectal region: ttest pval: 0.16567; tstat : 1; df : 17; sd : 0.0019067

Test against a Expected # of cells based on region size 0.47598

Pretectal region: ttest pval (scaled--expected based on size): 1; tstat : -1058.1259; df : 17; sd : 0.0019067

--------------------------

Cuneiform nucleus: Mean: 0.003247 +/- 0.00081034 SEM

Test against a mean of 0

Cuneiform nucleus: ttest pval: 0.16567; tstat : 1; df : 17; sd : 0.013776

Test against a Expected # of cells based on region size 0.1289

Cuneiform nucleus: ttest pval (scaled--expected based on size): 1; tstat : -38.6973; df : 17; sd : 0.013776

--------------------------

Red nucleus: Mean: 0.0024969 +/- 0.00062314 SEM

Test against a mean of 0

Red nucleus: ttest pval: 0.16567; tstat : 1; df : 17; sd : 0.010593

Test against a Expected # of cells based on region size 0.17216

Red nucleus: ttest pval (scaled--expected based on size): 1; tstat : -67.9517; df : 17; sd : 0.010593

--------------------------

Oculomotor nucleus: Mean: 0 +/- 0 SEM

Test against a mean of 0

Oculomotor nucleus: ttest pval: NaN; tstat : NaN; df : 17; sd : 0

Test against a Expected # of cells based on region size 0.0070762

Oculomotor nucleus: ttest pval (scaled--expected based on size): 1; tstat : -Inf; df : 17; sd : 0

--------------------------

Medial accesory oculomotor nucleus: Mean: 0 +/- 0 SEM

Test against a mean of 0

Medial accesory oculomotor nucleus: ttest pval: NaN; tstat : NaN; df : 17; sd : 0

Test against a Expected # of cells based on region size 0.0035313

Medial accesory oculomotor nucleus: ttest pval (scaled--expected based on size): 1; tstat : -Inf; df : 17; sd : 0

--------------------------

Edinger-Westphal nucleus: Mean: 0 +/- 0 SEM

Test against a mean of 0

Edinger-Westphal nucleus: ttest pval: NaN; tstat : NaN; df : 17; sd : 0

Test against a Expected # of cells based on region size 0.0056501

Edinger-Westphal nucleus: ttest pval (scaled--expected based on size): 1; tstat : -Inf; df : 17; sd : 0

--------------------------

Trochlear nucleus: Mean: 0 +/- 0 SEM

Test against a mean of 0

Trochlear nucleus: ttest pval: NaN; tstat : NaN; df : 17; sd : 0

Test against a Expected # of cells based on region size 0.0015415

Trochlear nucleus: ttest pval (scaled--expected based on size): 1; tstat : -Inf; df : 17; sd : 0

--------------------------

Paratrochlear nucleus: Mean: 0.00044941 +/- 0.00011216 SEM

Test against a mean of 0

Paratrochlear nucleus: ttest pval: 0.16567; tstat : 1; df : 17; sd : 0.0019067

Test against a Expected # of cells based on region size 0.0034804

Paratrochlear nucleus: ttest pval (scaled--expected based on size): 1; tstat : -6.7444; df : 17; sd : 0.0019067

--------------------------

Ventral tegmental nucleus: Mean: 0 +/- 0 SEM

Test against a mean of 0

Ventral tegmental nucleus: ttest pval: NaN; tstat : NaN; df : 17; sd : 0

Test against a Expected # of cells based on region size 0.0082103

Ventral tegmental nucleus: ttest pval (scaled--expected based on size): 1; tstat : -Inf; df : 17; sd : 0

--------------------------

Anterior tegmental nucleus: Mean: 0 +/- 0 SEM

Test against a mean of 0

Anterior tegmental nucleus: ttest pval: NaN; tstat : NaN; df : 17; sd : 0

Test against a Expected # of cells based on region size 0.0093647

Anterior tegmental nucleus: ttest pval (scaled--expected based on size): 1; tstat : -Inf; df : 17; sd : 0

--------------------------

Lateral terminal nucleus of the accessory optic tract: Mean: 0.0042103 +/- 0.0010508 SEM

Test against a mean of 0

Lateral terminal nucleus of the accessory optic tract: ttest pval: 0.16567; tstat : 1; df : 17; sd : 0.017863

Test against a Expected # of cells based on region size 0.004044

Lateral terminal nucleus of the accessory optic tract: ttest pval (scaled--expected based on size): 0.48447; tstat : 0.039508; df : 17; sd : 0.017863

--------------------------

Dorsal terminal nucleus of the accessory optic tract: Mean: 0 +/- 0 SEM

Test against a mean of 0

Dorsal terminal nucleus of the accessory optic tract: ttest pval: NaN; tstat : NaN; df : 17; sd : 0

Test against a Expected # of cells based on region size 0.0029235

Dorsal terminal nucleus of the accessory optic tract: ttest pval (scaled--expected based on size): 1; tstat : -Inf; df : 17; sd : 0

--------------------------

Medial terminal nucleus of the accessory optic tract: Mean: 0 +/- 0 SEM

Test against a mean of 0

Medial terminal nucleus of the accessory optic tract: ttest pval: NaN; tstat : NaN; df : 17; sd : 0

Test against a Expected # of cells based on region size 0.0094734

Medial terminal nucleus of the accessory optic tract: ttest pval (scaled--expected based on size): 1; tstat : -Inf; df : 17; sd : 0

--------------------------

Superior colliculus, sensory related: Mean: 0 +/- 0 SEM

Test against a mean of 0

Superior colliculus, sensory related: ttest pval: NaN; tstat : NaN; df : 17; sd : 0

Test against a Expected # of cells based on region size 0.47634

Superior colliculus, sensory related: ttest pval (scaled--expected based on size): 1; tstat : -Inf; df : 17; sd : 0

--------------------------

Inferior colliculus: Mean: 0.020925 +/- 0.0052222 SEM

Test against a mean of 0

Inferior colliculus: ttest pval: 0.16567; tstat : 1; df : 17; sd : 0.088777

Test against a Expected # of cells based on region size 0.96665

Inferior colliculus: ttest pval (scaled--expected based on size): 1; tstat : -45.1962; df : 17; sd : 0.088777

--------------------------

Nucleus of the brachium of the inferior colliculus: Mean: 0.010462 +/- 0.0026111 SEM

Test against a mean of 0

Nucleus of the brachium of the inferior colliculus: ttest pval: 0.16567; tstat : 1; df : 17; sd : 0.044388

Test against a Expected # of cells based on region size 0.029602

Nucleus of the brachium of the inferior colliculus: ttest pval (scaled--expected based on size): 0.95753; tstat : -1.8293; df : 17; sd : 0.044388

--------------------------

Nucleus sagulum: Mean: 0 +/- 0 SEM

Test against a mean of 0

Nucleus sagulum: ttest pval: NaN; tstat : NaN; df : 17; sd : 0

Test against a Expected # of cells based on region size 0.021344

Nucleus sagulum: ttest pval (scaled--expected based on size): 1; tstat : -Inf; df : 17; sd : 0

--------------------------

Parabigeminal nucleus: Mean: 0 +/- 0 SEM

Test against a mean of 0

Parabigeminal nucleus: ttest pval: NaN; tstat : NaN; df : 17; sd : 0

Test against a Expected # of cells based on region size 0.0096839

Parabigeminal nucleus: ttest pval (scaled--expected based on size): 1; tstat : -Inf; df : 17; sd : 0

--------------------------

Midbrain trigeminal nucleus: Mean: 0 +/- 0 SEM

Test against a mean of 0

Midbrain trigeminal nucleus: ttest pval: NaN; tstat : NaN; df : 17; sd : 0

Test against a Expected # of cells based on region size 0.0022003

Midbrain trigeminal nucleus: ttest pval (scaled--expected based on size): 1; tstat : -Inf; df : 17; sd : 0

--------------------------

Subcommissural organ: Mean: 0 +/- 0 SEM

Test against a mean of 0

Subcommissural organ: ttest pval: NaN; tstat : NaN; df : 17; sd : 0

Test against a Expected # of cells based on region size 0.0092832

Subcommissural organ: ttest pval (scaled--expected based on size): 1; tstat : -Inf; df : 17; sd : 0

--------------------------

Substantia nigra, compact part: Mean: 0.00044941 +/- 0.00011216 SEM

Test against a mean of 0

Substantia nigra, compact part: ttest pval: 0.16567; tstat : 1; df : 17; sd : 0.0019067

Test against a Expected # of cells based on region size 0.043051

Substantia nigra, compact part: ttest pval (scaled--expected based on size): 1; tstat : -94.7958; df : 17; sd : 0.0019067

--------------------------

Pedunculopontine nucleus: Mean: 0.0012484 +/- 0.00031157 SEM

Test against a mean of 0

Pedunculopontine nucleus: ttest pval: 0.16567; tstat : 1; df : 17; sd : 0.0052967

Test against a Expected # of cells based on region size 0.2086

Pedunculopontine nucleus: ttest pval (scaled--expected based on size): 1; tstat : -166.0893; df : 17; sd : 0.0052967

--------------------------

Interfascicular nucleus raphe: Mean: 0 +/- 0 SEM

Test against a mean of 0

Interfascicular nucleus raphe: ttest pval: NaN; tstat : NaN; df : 17; sd : 0

Test against a Expected # of cells based on region size 0.020156

Interfascicular nucleus raphe: ttest pval (scaled--expected based on size): 1; tstat : -Inf; df : 17; sd : 0

--------------------------

Interpeduncular nucleus: Mean: 0.00089881 +/- 0.00022431 SEM

Test against a mean of 0

Interpeduncular nucleus: ttest pval: 0.16567; tstat : 1; df : 17; sd : 0.0038133

Test against a Expected # of cells based on region size 0.079699

Interpeduncular nucleus: ttest pval (scaled--expected based on size): 1; tstat : -87.671; df : 17; sd : 0.0038133

--------------------------

Rostral linear nucleus raphe: Mean: 0 +/- 0 SEM

Test against a mean of 0

Rostral linear nucleus raphe: ttest pval: NaN; tstat : NaN; df : 17; sd : 0

Test against a Expected # of cells based on region size 0.016071

Rostral linear nucleus raphe: ttest pval (scaled--expected based on size): 1; tstat : -Inf; df : 17; sd : 0

--------------------------

Central linear nucleus raphe: Mean: 0 +/- 0 SEM

Test against a mean of 0

Central linear nucleus raphe: ttest pval: NaN; tstat : NaN; df : 17; sd : 0

Test against a Expected # of cells based on region size 0.020332

Central linear nucleus raphe: ttest pval (scaled--expected based on size): 1; tstat : -Inf; df : 17; sd : 0

--------------------------

Dorsal nucleus raphe: Mean: 0.0036964 +/- 0.0008115 SEM

Test against a mean of 0

Dorsal nucleus raphe: ttest pval: 0.1357; tstat : 1.1368; df : 17; sd : 0.013795

Test against a Expected # of cells based on region size 0.033422

Dorsal nucleus raphe: ttest pval (scaled--expected based on size): 1; tstat : -9.1417; df : 17; sd : 0.013795

--------------------------

Primary motor area: Mean: 0.006686 +/- 0.0012921 SEM

Test against a mean of 0

Primary motor area: ttest pval: 0.10693; tstat : 1.2913; df : 17; sd : 0.021966

Test against a Expected # of cells based on region size 2.6186

Primary motor area: ttest pval (scaled--expected based on size): 1; tstat : -504.4714; df : 17; sd : 0.021966

--------------------------

Secondary motor area: Mean: 0.074756 +/- 0.016114 SEM

Test against a mean of 0

Secondary motor area: ttest pval: 0.13147; tstat : 1.1578; df : 17; sd : 0.27393

Test against a Expected # of cells based on region size 2.9481

Secondary motor area: ttest pval (scaled--expected based on size): 1; tstat : -44.5026; df : 17; sd : 0.27393

--------------------------

primary somatosensory area: Mean: 0.0027917 +/- 0.0004482 SEM

Test against a mean of 0

primary somatosensory area: ttest pval: 0.069247; tstat : 1.5545; df : 17; sd : 0.0076194

Test against a Expected # of cells based on region size 5.5681

primary somatosensory area: ttest pval (scaled--expected based on size): 1; tstat : -3098.8479; df : 17; sd : 0.0076194

--------------------------

Supplemental somatosensory area: Mean: 0.0063329 +/- 0.0011044 SEM

Test against a mean of 0

Supplemental somatosensory area: ttest pval: 0.085266; tstat : 1.4311; df : 17; sd : 0.018775

Test against a Expected # of cells based on region size 2.0429

Supplemental somatosensory area: ttest pval (scaled--expected based on size): 1; tstat : -460.2201; df : 17; sd : 0.018775

--------------------------

Gustatory areas: Mean: 0.0050837 +/- 0.00097392 SEM

Test against a mean of 0

Gustatory areas: ttest pval: 0.10502; tstat : 1.3027; df : 17; sd : 0.016557

Test against a Expected # of cells based on region size 0.38663

Gustatory areas: ttest pval (scaled--expected based on size): 1; tstat : -97.7722; df : 17; sd : 0.016557

--------------------------

Visceral area: Mean: 0.013856 +/- 0.0013483 SEM

Test against a mean of 0

Visceral area: ttest pval: 0.010045; tstat : 2.5647; df : 17; sd : 0.022921

Test against a Expected # of cells based on region size 0.52504

Visceral area: ttest pval (scaled--expected based on size): 1; tstat : -94.6193; df : 17; sd : 0.022921

--------------------------

Auditory areas: Mean: 0.0047348 +/- 0.00071797 SEM

Test against a mean of 0

Auditory areas: ttest pval: 0.05908; tstat : 1.6458; df : 17; sd : 0.012206

Test against a Expected # of cells based on region size 1.3473

Auditory areas: ttest pval (scaled--expected based on size): 1; tstat : -466.6904; df : 17; sd : 0.012206

--------------------------

Visual areas: Mean: 0.008255 +/- 0.0014136 SEM

Test against a mean of 0

Visual areas: ttest pval: 0.081613; tstat : 1.4574; df : 17; sd : 0.024031

Test against a Expected # of cells based on region size 3.1429

Visual areas: ttest pval (scaled--expected based on size): 1; tstat : -553.4224; df : 17; sd : 0.024031

--------------------------

Anterior cingulate area: Mean: 0.010989 +/- 0.0024326 SEM

Test against a mean of 0

Anterior cingulate area: ttest pval: 0.13762; tstat : 1.1274; df : 17; sd : 0.041355

Test against a Expected # of cells based on region size 1.3819

Anterior cingulate area: ttest pval (scaled--expected based on size): 1; tstat : -140.646; df : 17; sd : 0.041355

--------------------------

Prelimbic area: Mean: 0.0012484 +/- 0.00031157 SEM

Test against a mean of 0

Prelimbic area: ttest pval: 0.16567; tstat : 1; df : 17; sd : 0.0052967

Test against a Expected # of cells based on region size 0.54511

Prelimbic area: ttest pval (scaled--expected based on size): 1; tstat : -435.6325; df : 17; sd : 0.0052967

--------------------------

Infralimbic area: Mean: 0.014772 +/- 0.0015668 SEM

Test against a mean of 0

Infralimbic area: ttest pval: 0.01546; tstat : 2.3531; df : 17; sd : 0.026635

Test against a Expected # of cells based on region size 0.20061

Infralimbic area: ttest pval (scaled--expected based on size): 1; tstat : -29.6022; df : 17; sd : 0.026635

--------------------------

Orbital area: Mean: 0.030667 +/- 0.0049647 SEM

Test against a mean of 0

Orbital area: ttest pval: 0.070793; tstat : 1.5416; df : 17; sd : 0.0844

Test against a Expected # of cells based on region size 1.3105

Orbital area: ttest pval (scaled--expected based on size): 1; tstat : -64.3337; df : 17; sd : 0.0844

--------------------------

Agranular insular area: Mean: 0.074015 +/- 0.0062399 SEM

Test against a mean of 0

Agranular insular area: ttest pval: 0.0043832; tstat : 2.9602; df : 17; sd : 0.10608

Test against a Expected # of cells based on region size 1.7341

Agranular insular area: ttest pval (scaled--expected based on size): 1; tstat : -66.3957; df : 17; sd : 0.10608

--------------------------

Retrosplenial area: Mean: 0 +/- 0 SEM

Test against a mean of 0

Retrosplenial area: ttest pval: NaN; tstat : NaN; df : 17; sd : 0

Test against a Expected # of cells based on region size 2.5553

Retrosplenial area: ttest pval (scaled--expected based on size): 1; tstat : -Inf; df : 17; sd : 0

--------------------------

Posterior parietal association areas: Mean: 0 +/- 0 SEM

Test against a mean of 0

Posterior parietal association areas: ttest pval: NaN; tstat : NaN; df : 17; sd : 0

Test against a Expected # of cells based on region size 0.57144

Posterior parietal association areas: ttest pval (scaled--expected based on size): 1; tstat : -Inf; df : 17; sd : 0

--------------------------

Temporal association areas: Mean: 0.033418 +/- 0.0048174 SEM

Test against a mean of 0

Temporal association areas: ttest pval: 0.05076; tstat : 1.7312; df : 17; sd : 0.081896

Test against a Expected # of cells based on region size 0.71655

Temporal association areas: ttest pval (scaled--expected based on size): 1; tstat : -35.3897; df : 17; sd : 0.081896

--------------------------

Perirhinal area: Mean: 0.00044941 +/- 0.00011216 SEM

Test against a mean of 0

Perirhinal area: ttest pval: 0.16567; tstat : 1; df : 17; sd : 0.0019067

Test against a Expected # of cells based on region size 0.17514

Perirhinal area: ttest pval (scaled--expected based on size): 1; tstat : -388.7036; df : 17; sd : 0.0019067

--------------------------

Ectorhinal area: Mean: 0.02416 +/- 0.0046767 SEM

Test against a mean of 0

Ectorhinal area: ttest pval: 0.10728; tstat : 1.2893; df : 17; sd : 0.079503

Test against a Expected # of cells based on region size 0.39531

Ectorhinal area: ttest pval (scaled--expected based on size): 1; tstat : -19.8065; df : 17; sd : 0.079503

--------------------------

cerebellar cortex: Mean: 0.0087391 +/- 0.002181 SEM

Test against a mean of 0

cerebellar cortex: ttest pval: 0.16567; tstat : 1; df : 17; sd : 0.037077

Test against a Expected # of cells based on region size 12.5699

cerebellar cortex: ttest pval (scaled--expected based on size): 1; tstat : -1437.3559; df : 17; sd : 0.037077

--------------------------

Fastigial nucleus: Mean: 0 +/- 0 SEM

Test against a mean of 0

Fastigial nucleus: ttest pval: NaN; tstat : NaN; df : 17; sd : 0

Test against a Expected # of cells based on region size 0.19496

Fastigial nucleus: ttest pval (scaled--expected based on size): 1; tstat : -Inf; df : 17; sd : 0

--------------------------

Interposed nucleus: Mean: 0 +/- 0 SEM

Test against a mean of 0

Interposed nucleus: ttest pval: NaN; tstat : NaN; df : 17; sd : 0

Test against a Expected # of cells based on region size 0.30222

Interposed nucleus: ttest pval (scaled--expected based on size): 1; tstat : -Inf; df : 17; sd : 0

--------------------------

Dentate nucleus: Mean: 0 +/- 0 SEM

Test against a mean of 0

Dentate nucleus: ttest pval: NaN; tstat : NaN; df : 17; sd : 0

Test against a Expected # of cells based on region size 0.13733

Dentate nucleus: ttest pval (scaled--expected based on size): 1; tstat : -Inf; df : 17; sd : 0

--------------------------

Vestibulocerebellar nucleus: Mean: 0 +/- 0 SEM

Test against a mean of 0

Vestibulocerebellar nucleus: ttest pval: NaN; tstat : NaN; df : 17; sd : 0

Test against a Expected # of cells based on region size 0.018814

Vestibulocerebellar nucleus: ttest pval (scaled--expected based on size): 1; tstat : -Inf; df : 17; sd : 0

--------------------------

Ventral group of the dorsal thalamus: Mean: 0.33454 +/- 0.039723 SEM

Test against a mean of 0

Ventral group of the dorsal thalamus: ttest pval: 0.025392; tstat : 2.1018; df : 17; sd : 0.6753

Test against a Expected # of cells based on region size 1.0937

Ventral group of the dorsal thalamus: ttest pval (scaled--expected based on size): 0.99991; tstat : -4.7692; df : 17; sd : 0.6753

--------------------------

Subparafascicular nucleus: Mean: 0 +/- 0 SEM

Test against a mean of 0

Subparafascicular nucleus: ttest pval: NaN; tstat : NaN; df : 17; sd : 0

Test against a Expected # of cells based on region size 0.044905

Subparafascicular nucleus: ttest pval (scaled--expected based on size): 1; tstat : -Inf; df : 17; sd : 0

--------------------------

Subparafascicular area: Mean: 0 +/- 0 SEM

Test against a mean of 0

Subparafascicular area: ttest pval: NaN; tstat : NaN; df : 17; sd : 0

Test against a Expected # of cells based on region size 0.028753

Subparafascicular area: ttest pval (scaled--expected based on size): 1; tstat : -Inf; df : 17; sd : 0

--------------------------

Peripeduncular nucleus: Mean: 0.058784 +/- 0.010232 SEM

Test against a mean of 0

Peripeduncular nucleus: ttest pval: 0.084889; tstat : 1.4338; df : 17; sd : 0.17395

Test against a Expected # of cells based on region size 0.01455

Peripeduncular nucleus: ttest pval (scaled--expected based on size): 0.14785; tstat : 1.0789; df : 17; sd : 0.17395

--------------------------

Geniculate group, dorsal thalamus: Mean: 0.16823 +/- 0.033751 SEM

Test against a mean of 0

Geniculate group, dorsal thalamus: ttest pval: 0.1152; tstat : 1.244; df : 17; sd : 0.57377

Test against a Expected # of cells based on region size 0.35506

Geniculate group, dorsal thalamus: ttest pval (scaled--expected based on size): 0.90749; tstat : -1.3815; df : 17; sd : 0.57377

--------------------------

Lateral group of the dorsal thalamus: Mean: 0.041493 +/- 0.0052111 SEM

Test against a mean of 0

Lateral group of the dorsal thalamus: ttest pval: 0.031631; tstat : 1.9872; df : 17; sd : 0.088588

Test against a Expected # of cells based on region size 0.68209

Lateral group of the dorsal thalamus: ttest pval (scaled--expected based on size): 1; tstat : -30.6791; df : 17; sd : 0.088588

--------------------------

Anterior group of the dorsal thalamus: Mean: 0 +/- 0 SEM

Test against a mean of 0

Anterior group of the dorsal thalamus: ttest pval: NaN; tstat : NaN; df : 17; sd : 0

Test against a Expected # of cells based on region size 0.57039

Anterior group of the dorsal thalamus: ttest pval (scaled--expected based on size): 1; tstat : -Inf; df : 17; sd : 0

--------------------------

Medial group of the dorsal thalamus: Mean: 0.00711 +/- 0.00096338 SEM

Test against a mean of 0

Medial group of the dorsal thalamus: ttest pval: 0.041505; tstat : 1.8419; df : 17; sd : 0.016378

Test against a Expected # of cells based on region size 0.45517

Medial group of the dorsal thalamus: ttest pval (scaled--expected based on size): 1; tstat : -116.072; df : 17; sd : 0.016378

--------------------------

Midline group of the dorsal thalamus: Mean: 0.11222 +/- 0.010624 SEM

Test against a mean of 0

Midline group of the dorsal thalamus: ttest pval: 0.0086697; tstat : 2.6359; df : 17; sd : 0.18062

Test against a Expected # of cells based on region size 0.28566

Midline group of the dorsal thalamus: ttest pval (scaled--expected based on size): 0.99961; tstat : -4.0743; df : 17; sd : 0.18062

--------------------------

Intralaminar nuclei of the dorsal thalamus: Mean: 0.074545 +/- 0.012797 SEM

Test against a mean of 0

Intralaminar nuclei of the dorsal thalamus: ttest pval: 0.082118; tstat : 1.4537; df : 17; sd : 0.21756

Test against a Expected # of cells based on region size 0.36311

Intralaminar nuclei of the dorsal thalamus: ttest pval (scaled--expected based on size): 0.99998; tstat : -5.6275; df : 17; sd : 0.21756

--------------------------

Reticular nucleus of the thalamus: Mean: 0.87295 +/- 0.14077 SEM

Test against a mean of 0

Reticular nucleus of the thalamus: ttest pval: 0.070059; tstat : 1.5477; df : 17; sd : 2.3931

Test against a Expected # of cells based on region size 0.49022

Reticular nucleus of the thalamus: ttest pval (scaled--expected based on size): 0.25328; tstat : 0.67855; df : 17; sd : 2.3931

--------------------------

Geniculate group, ventral thalamus: Mean: 0.076796 +/- 0.0089385 SEM

Test against a mean of 0

Geniculate group, ventral thalamus: ttest pval: 0.023384; tstat : 2.1442; df : 17; sd : 0.15195

Test against a Expected # of cells based on region size 0.17279

Geniculate group, ventral thalamus: ttest pval (scaled--expected based on size): 0.99209; tstat : -2.6803; df : 17; sd : 0.15195

--------------------------

Epithalamus: Mean: 0.00089881 +/- 0.00022431 SEM

Test against a mean of 0

Epithalamus: ttest pval: 0.16567; tstat : 1; df : 17; sd : 0.0038133

Test against a Expected # of cells based on region size 0.22435

Epithalamus: ttest pval (scaled--expected based on size): 1; tstat : -248.6063; df : 17; sd : 0.0038133

--------------------------

Caudoputanum: Mean: 6.5636 +/- 0.63899 SEM

Test against a mean of 0

Caudoputanum: ttest pval: 0.010071; tstat : 2.5635; df : 17; sd : 10.8628

Test against a Expected # of cells based on region size 6.55

Caudoputanum: ttest pval (scaled--expected based on size): 0.49791; tstat : 0.0053252; df : 17; sd : 10.8628

--------------------------

Nucleus accumbens: Mean: 0.14857 +/- 0.014201 SEM

Test against a mean of 0

Nucleus accumbens: ttest pval: 0.0091294; tstat : 2.611; df : 17; sd : 0.24141

Test against a Expected # of cells based on region size 0.99813

Nucleus accumbens: ttest pval (scaled--expected based on size): 1; tstat : -14.9302; df : 17; sd : 0.24141

--------------------------

Fundus of striatum: Mean: 0.11367 +/- 0.011312 SEM

Test against a mean of 0

Fundus of striatum: ttest pval: 0.011294; tstat : 2.5077; df : 17; sd : 0.19231

Test against a Expected # of cells based on region size 0.093834

Fundus of striatum: ttest pval (scaled--expected based on size): 0.33359; tstat : 0.43761; df : 17; sd : 0.19231

--------------------------

Olfactory Tubercle: Mean: 0.22479 +/- 0.044624 SEM

Test against a mean of 0

Olfactory Tubercle: ttest pval: 0.11284; tstat : 1.2572; df : 17; sd : 0.75861

Test against a Expected # of cells based on region size 0.84157

Olfactory Tubercle: ttest pval (scaled--expected based on size): 0.99847; tstat : -3.4494; df : 17; sd : 0.75861

--------------------------

Lateral septal nucleus: Mean: 0.14769 +/- 0.012135 SEM

Test against a mean of 0

Lateral septal nucleus: ttest pval: 0.0037178; tstat : 3.0375; df : 17; sd : 0.20629

Test against a Expected # of cells based on region size 0.79092

Lateral septal nucleus: ttest pval (scaled--expected based on size): 1; tstat : -13.2287; df : 17; sd : 0.20629

--------------------------

Septofimbrial nucleus: Mean: 0.046789 +/- 0.0060092 SEM

Test against a mean of 0

Septofimbrial nucleus: ttest pval: 0.034372; tstat : 1.9432; df : 17; sd : 0.10216

Test against a Expected # of cells based on region size 0.17297

Septofimbrial nucleus: ttest pval (scaled--expected based on size): 0.99997; tstat : -5.2403; df : 17; sd : 0.10216

--------------------------

Septohippocampal nucleus: Mean: 0 +/- 0 SEM

Test against a mean of 0

Septohippocampal nucleus: ttest pval: NaN; tstat : NaN; df : 17; sd : 0

Test against a Expected # of cells based on region size 0.0097178

Septohippocampal nucleus: ttest pval (scaled--expected based on size): 1; tstat : -Inf; df : 17; sd : 0

--------------------------

Anterior amygdalar area: Mean: 0.25848 +/- 0.021208 SEM

Test against a mean of 0

Anterior amygdalar area: ttest pval: 0.003685; tstat : 3.0416; df : 17; sd : 0.36054

Test against a Expected # of cells based on region size 0.10747

Anterior amygdalar area: ttest pval (scaled--expected based on size): 0.046736; tstat : 1.7769; df : 17; sd : 0.36054

--------------------------

Bed nucleus of the accessory olfactory tract: Mean: 0.0012484 +/- 0.00031157 SEM

Test against a mean of 0

Bed nucleus of the accessory olfactory tract: ttest pval: 0.16567; tstat : 1; df : 17; sd : 0.0052967

Test against a Expected # of cells based on region size 0.0054463

Bed nucleus of the accessory olfactory tract: ttest pval (scaled--expected based on size): 0.99815; tstat : -3.3625; df : 17; sd : 0.0052967

--------------------------

Central amygdalar nucleus: Mean: 11.3864 +/- 0.59917 SEM

Test against a mean of 0

Central amygdalar nucleus: ttest pval: 9.4177e-05; tstat : 4.7427; df : 17; sd : 10.1858

Test against a Expected # of cells based on region size 0.2958

Central amygdalar nucleus: ttest pval (scaled--expected based on size): 0.00012234; tstat : 4.6195; df : 17; sd : 10.1858

--------------------------

Intercalated amygdalar nucleus: Mean: 0.43087 +/- 0.039931 SEM

Test against a mean of 0

Intercalated amygdalar nucleus: ttest pval: 0.0077006; tstat : 2.6929; df : 17; sd : 0.67883

Test against a Expected # of cells based on region size 0.039897

Intercalated amygdalar nucleus: ttest pval (scaled--expected based on size): 0.012874; tstat : 2.4436; df : 17; sd : 0.67883

--------------------------

Medial amygdalar nucleus: Mean: 20.0323 +/- 1.3142 SEM

Test against a mean of 0

Medial amygdalar nucleus: ttest pval: 0.00070912; tstat : 3.8041; df : 17; sd : 22.3418

Test against a Expected # of cells based on region size 0.5476

Medial amygdalar nucleus: ttest pval (scaled--expected based on size): 0.00088875; tstat : 3.7001; df : 17; sd : 22.3418

--------------------------

Globus pallidus, external segment: Mean: 3.2419 +/- 0.30863 SEM

Test against a mean of 0

Globus pallidus, external segment: ttest pval: 0.0089331; tstat : 2.6215; df : 17; sd : 5.2467

Test against a Expected # of cells based on region size 0.40648

Globus pallidus, external segment: ttest pval (scaled--expected based on size): 0.017442; tstat : 2.2928; df : 17; sd : 5.2467

--------------------------

Globus pallidus, internal segment: Mean: 1.4163 +/- 0.11891 SEM

Test against a mean of 0

Globus pallidus, internal segment: ttest pval: 0.004272; tstat : 2.9723; df : 17; sd : 2.0215

Test against a Expected # of cells based on region size 0.1381

Globus pallidus, internal segment: ttest pval (scaled--expected based on size): 0.00787; tstat : 2.6825; df : 17; sd : 2.0215

--------------------------

Substantia innominata: Mean: 2.5134 +/- 0.19436 SEM

Test against a mean of 0

Substantia innominata: ttest pval: 0.0024738; tstat : 3.2273; df : 17; sd : 3.3041

Test against a Expected # of cells based on region size 0.65833

Substantia innominata: ttest pval (scaled--expected based on size): 0.014584; tstat : 2.382; df : 17; sd : 3.3041

--------------------------

Magnocellular nucleus: Mean: 0.27731 +/- 0.021054 SEM

Test against a mean of 0

Magnocellular nucleus: ttest pval: 0.0021749; tstat : 3.2871; df : 17; sd : 0.35792

Test against a Expected # of cells based on region size 0.079227

Magnocellular nucleus: ttest pval (scaled--expected based on size): 0.015619; tstat : 2.348; df : 17; sd : 0.35792

--------------------------

Medial septal nucleus: Mean: 0.18787 +/- 0.018921 SEM

Test against a mean of 0

Medial septal nucleus: ttest pval: 0.012004; tstat : 2.4779; df : 17; sd : 0.32167

Test against a Expected # of cells based on region size 0.09107

Medial septal nucleus: ttest pval (scaled--expected based on size): 0.10943; tstat : 1.2767; df : 17; sd : 0.32167

--------------------------

Diagonal band nucleus: Mean: 0.48947 +/- 0.033737 SEM

Test against a mean of 0

Diagonal band nucleus: ttest pval: 0.0010556; tstat : 3.6208; df : 17; sd : 0.57353

Test against a Expected # of cells based on region size 0.16087

Diagonal band nucleus: ttest pval (scaled--expected based on size): 0.013211; tstat : 2.4308; df : 17; sd : 0.57353

--------------------------

Triangular nucleus of septum: Mean: 0.0064051 +/- 0.00088501 SEM

Test against a mean of 0

Triangular nucleus of septum: ttest pval: 0.044312; tstat : 1.8062; df : 17; sd : 0.015045

Test against a Expected # of cells based on region size 0.11501

Triangular nucleus of septum: ttest pval (scaled--expected based on size): 1; tstat : -30.6272; df : 17; sd : 0.015045

--------------------------

BNST: Mean: 0.61112 +/- 0.043287 SEM

Test against a mean of 0

BNST: ttest pval: 0.0013042; tstat : 3.5234; df : 17; sd : 0.73588

Test against a Expected # of cells based on region size 0.33723

BNST: ttest pval (scaled--expected based on size): 0.066372; tstat : 1.5791; df : 17; sd : 0.73588

--------------------------

MainOlfactoryBulb: Mean: 0.25171 +/- 0.038879 SEM

Test against a mean of 0

MainOlfactoryBulb: ttest pval: 0.062276; tstat : 1.6158; df : 17; sd : 0.66094

Test against a Expected # of cells based on region size 4.2909

MainOlfactoryBulb: ttest pval (scaled--expected based on size): 1; tstat : -25.9276; df : 17; sd : 0.66094

--------------------------

AccessoryOlfactoryBulb: Mean: 0.92978 +/- 0.097154 SEM

Test against a mean of 0

AccessoryOlfactoryBulb: ttest pval: 0.014397; tstat : 2.3884; df : 17; sd : 1.6516

Test against a Expected # of cells based on region size 0.16047

AccessoryOlfactoryBulb: ttest pval (scaled--expected based on size): 0.032296; tstat : 1.9762; df : 17; sd : 1.6516

--------------------------

AnteriorOlfactoryNucleus: Mean: 0.0037733 +/- 0.00069434 SEM

Test against a mean of 0

AnteriorOlfactoryNucleus: ttest pval: 0.096383; tstat : 1.3562; df : 17; sd : 0.011804

Test against a Expected # of cells based on region size 1.168

AnteriorOlfactoryNucleus: ttest pval (scaled--expected based on size): 1; tstat : -418.465; df : 17; sd : 0.011804

--------------------------

Taenia tecta: Mean: 0.003908 +/- 0.00062019 SEM

Test against a mean of 0

Taenia tecta: ttest pval: 0.06712; tstat : 1.5726; df : 17; sd : 0.010543

Test against a Expected # of cells based on region size 0.31398

Taenia tecta: ttest pval (scaled--expected based on size): 1; tstat : -124.7746; df : 17; sd : 0.010543

--------------------------

Dorsal peduncular area: Mean: 0.001602 +/- 0.0002845 SEM

Test against a mean of 0

Dorsal peduncular area: ttest pval: 0.088968; tstat : 1.4053; df : 17; sd : 0.0048365

Test against a Expected # of cells based on region size 0.12319

Dorsal peduncular area: ttest pval (scaled--expected based on size): 1; tstat : -106.657; df : 17; sd : 0.0048365

--------------------------

Piriform area: Mean: 1.0382 +/- 0.05887 SEM

Test against a mean of 0

Piriform area: ttest pval: 0.00019501; tstat : 4.4014; df : 17; sd : 1.0008

Test against a Expected # of cells based on region size 2.6015

Piriform area: ttest pval (scaled--expected based on size): 1; tstat : -6.6274; df : 17; sd : 1.0008

--------------------------

Nucleus of the lateral olfactory tract: Mean: 0.21477 +/- 0.033776 SEM

Test against a mean of 0

Nucleus of the lateral olfactory tract: ttest pval: 0.06548; tstat : 1.5869; df : 17; sd : 0.5742

Test against a Expected # of cells based on region size 0.070684

Nucleus of the lateral olfactory tract: ttest pval (scaled--expected based on size): 0.15097; tstat : 1.0646; df : 17; sd : 0.5742

--------------------------

Cortical amygdalar area: Mean: 3.9576 +/- 0.26051 SEM

Test against a mean of 0

Cortical amygdalar area: ttest pval: 0.0007289; tstat : 3.7914; df : 17; sd : 4.4286

Test against a Expected # of cells based on region size 0.70976

Cortical amygdalar area: ttest pval (scaled--expected based on size): 0.0031734; tstat : 3.1114; df : 17; sd : 4.4286

--------------------------

Piriform-amygdalar area: Mean: 0.37813 +/- 0.021107 SEM

Test against a mean of 0

Piriform-amygdalar area: ttest pval: 0.00016796; tstat : 4.4711; df : 17; sd : 0.35881

Test against a Expected # of cells based on region size 0.25921

Piriform-amygdalar area: ttest pval (scaled--expected based on size): 0.088843; tstat : 1.4062; df : 17; sd : 0.35881

--------------------------

Postpiriform transition area: Mean: 0.33813 +/- 0.030562 SEM

Test against a mean of 0

Postpiriform transition area: ttest pval: 0.0066758; tstat : 2.7612; df : 17; sd : 0.51955

Test against a Expected # of cells based on region size 0.30535

Postpiriform transition area: ttest pval (scaled--expected based on size): 0.3961; tstat : 0.26765; df : 17; sd : 0.51955

--------------------------

Claustrum: Mean: 0.0042561 +/- 0.00083431 SEM

Test against a mean of 0

Claustrum: ttest pval: 0.11005; tstat : 1.2731; df : 17; sd : 0.014183

Test against a Expected # of cells based on region size 0.12164

Claustrum: ttest pval (scaled--expected based on size): 1; tstat : -35.113; df : 17; sd : 0.014183

--------------------------

Endopiriform nucleus-dorsal: Mean: 0.039201 +/- 0.0035084 SEM

Test against a mean of 0

Endopiriform nucleus-dorsal: ttest pval: 0.0063029; tstat : 2.7885; df : 17; sd : 0.059643

Test against a Expected # of cells based on region size 0.43775

Endopiriform nucleus-dorsal: ttest pval (scaled--expected based on size): 1; tstat : -28.3509; df : 17; sd : 0.059643

--------------------------

Endopiriform nucleus-ventral: Mean: 0.085026 +/- 0.0061553 SEM

Test against a mean of 0

Endopiriform nucleus-ventral: ttest pval: 0.0015377; tstat : 3.4474; df : 17; sd : 0.10464

Test against a Expected # of cells based on region size 0.21637

Endopiriform nucleus-ventral: ttest pval (scaled--expected based on size): 0.99997; tstat : -5.3255; df : 17; sd : 0.10464

--------------------------

Lateral amygdalar nucleus: Mean: 0.096562 +/- 0.010483 SEM

Test against a mean of 0

Lateral amygdalar nucleus: ttest pval: 0.017234; tstat : 2.2988; df : 17; sd : 0.17821

Test against a Expected # of cells based on region size 0.20089

Lateral amygdalar nucleus: ttest pval (scaled--expected based on size): 0.98814; tstat : -2.4838; df : 17; sd : 0.17821

--------------------------

Basolateral amygdalar nucleus-anterior: Mean: 0.59024 +/- 0.056393 SEM

Test against a mean of 0

Basolateral amygdalar nucleus-anterior: ttest pval: 0.0091088; tstat : 2.6121; df : 17; sd : 0.95868

Test against a Expected # of cells based on region size 0.17252

Basolateral amygdalar nucleus-anterior: ttest pval (scaled--expected based on size): 0.040991; tstat : 1.8486; df : 17; sd : 0.95868

--------------------------

Basolateral amygdalar nucleus-posterior: Mean: 0.26929 +/- 0.023909 SEM

Test against a mean of 0

Basolateral amygdalar nucleus-posterior: ttest pval: 0.0060123; tstat : 2.811; df : 17; sd : 0.40645

Test against a Expected # of cells based on region size 0.15594

Basolateral amygdalar nucleus-posterior: ttest pval (scaled--expected based on size): 0.1265; tstat : 1.1833; df : 17; sd : 0.40645

--------------------------

Basolateral amygdalar nucleus-ventral: Mean: 0.30243 +/- 0.0294 SEM

Test against a mean of 0

Basolateral amygdalar nucleus-ventral: ttest pval: 0.0099945; tstat : 2.5672; df : 17; sd : 0.4998

Test against a Expected # of cells based on region size 0.091039

Basolateral amygdalar nucleus-ventral: ttest pval (scaled--expected based on size): 0.045276; tstat : 1.7944; df : 17; sd : 0.4998

--------------------------

Basomedial amygdalar nucleus -anterior: Mean: 1.5901 +/- 0.12207 SEM

Test against a mean of 0

Basomedial amygdalar nucleus -anterior: ttest pval: 0.0023508; tstat : 3.251; df : 17; sd : 2.0751

Test against a Expected # of cells based on region size 0.16715

Basomedial amygdalar nucleus -anterior: ttest pval (scaled--expected based on size): 0.0048844; tstat : 2.9093; df : 17; sd : 2.0751

--------------------------

Basomedial amygdalar nucleus-posterior: Mean: 1.623 +/- 0.12082 SEM

Test against a mean of 0

Basomedial amygdalar nucleus-posterior: ttest pval: 0.0018883; tstat : 3.3525; df : 17; sd : 2.054

Test against a Expected # of cells based on region size 0.15629

Basomedial amygdalar nucleus-posterior: ttest pval (scaled--expected based on size): 0.0037805; tstat : 3.0296; df : 17; sd : 2.054

--------------------------

Posterior amygdalar nucleus: Mean: 3.7539 +/- 0.23612 SEM

Test against a mean of 0

Posterior amygdalar nucleus: ttest pval: 0.00049723; tstat : 3.9677; df : 17; sd : 4.014

Test against a Expected # of cells based on region size 0.21998

Posterior amygdalar nucleus: ttest pval (scaled--expected based on size): 0.00082354; tstat : 3.7352; df : 17; sd : 4.014

--------------------------
